# Exploratory analysis of the potential impact of violence on HIV among female sex workers in Mombasa, Kenya: a mathematical modelling study

**DOI:** 10.1186/s12916-024-03670-y

**Published:** 2024-10-15

**Authors:** Michael Pickles, Elisa Mountain, Parinita Bhattacharjee, Japheth Kioko, Janet Musimbi, Helgar Musyoki, Peter Gichangi, James Stannah, Mathieu Maheu-Giroux, Marissa Becker, Marie-Claude Boily

**Affiliations:** 1 Medical Research Council Centre for Global Infectious Disease Analysis, School of Public Health, Imperial College London, London, UK; 2 Institute for Global Public Health, University of Manitoba, Winnipeg, Canada; 3 Partners for Health and Development in Africa, Nairobi, Kenya; 4 National AIDS and STI Control Programme, Ministry of Health, Nairobi, Kenya; 5 Technical University of Mombasa, Mombasa, Kenya; 6 Department of Public Health and Primary Care, Faculty of Medicine and Health Sciences, Ghent University, Ghent, Belgium; 7 Department of Epidemiology and Biostatistics, School of Population and Global Health, McGill University, Montreal, Canada

**Keywords:** HIV, AIDS, Structural determinants, Structural interventions, Mathematical modelling, Causal pathways, Female, Sex work, Violence, Condom use, Antiretroviral therapy, Kenya

## Abstract

**Background:**

Understanding the frequency of violence experienced by female sex workers (FSWs) and how violence contributes to HIV transmission can help improve HIV programs.

**Methods:**

Using recent recommendations for modelling structural factors and associated causal pathways, we developed a HIV transmission dynamic model for FSWs and their clients in Mombasa, Kenya, mechanistically representing three types of violence (sexual violence, SV; physical violence, PV; police assault and arrest, PAA). Each type of violence affects HIV transmission through key mediators (condom non-use, HIV testing). We parameterized the model using data from a cross-sectional study of FSWs aged 15–24 recruited from a systematic geographical mapping sampling frame in Mombasa, Kenya (Cheuk E et al., Frontiers in Reproductive Health 2(7), 2020). Using this model, calibrated (and cross-validated) to HIV epidemiological and violence outcomes, we estimated the incidence of violence episodes, the contribution of violence to the HIV epidemic measured by the transmission population-attributable fraction, and the potential impact of possible violence interventions.

**Results:**

The median estimated incidence of PAA in 2023 among FSWs who had not previously experienced that type of violence was 0.20 (95% credible interval: 0.17–0.22) per person-year (ppy), about double the incidence of SV and PV (0.10 (0.09–0.11), 0.11 (0.09–0.12), respectively). The incidence of violence was higher among FSWs who had previously experienced violence: the incidence of recurrent PV was 2.65 (1.82–3.37) ppy, while the incidence of recurrent SV and PAA were 1.26 (0.80–1.67) and 1.37 (0.94–1.74 ppy, respectively. In this setting, we estimated that a median of 35.3% (3.4–55.8%) infections in FSWs and clients combined over the next 10 years may be due to all types of violence (and mediators), mainly through reduced condom use in FSWs who have ever experienced SV (34.6% (2.4–55.5%)). Interventions that prevent future violence without mitigating the effects of past violence may only prevent 8.8% (0.8–14.0%) infections over 10 years.

**Conclusions:**

FSWs in Mombasa experience violence frequently. In this population, we find that addressing sexual violence, including mitigating the effects of past violence, is potentially important in reducing HIV transmission in this population. However, the wide uncertainty range shows longitudinal studies are needed to strengthen the evidence of the influence of violence on HIV risk behavior. We find that the recommendations for modelling structural factors provide a useful framework for describing the model.

**Supplementary Information:**

The online version contains supplementary material available at 10.1186/s12916-024-03670-y.

## Background

Female sex workers (FSWs) experience many types of violence, including sexual violence (SV) and physical violence (PV) where perpetrators can include clients and intimate partners, as well as police assault and arrest (PAA) [1]. Violence from clients has been shown to be associated with reduced ability to access HIV prevention [2–5] and treatment [5, 6] services and negotiate condom use [2], while condom breakage is more likely during forced sex [7]. Intimate partner violence (IPV) may increase FSWs’ alcohol or drug use, further affecting their ability to practice safe sex during sex work [8] and may reduce engagement in HIV care among FSWs living with HIV [9, 10]. FSWs worldwide experience extremely high levels of violence, with 8–76% of FSWs reporting physical or sexual violence from clients and 4–64% reporting physical or sexual IPV [11] over a range of recall periods. Given their high prevalence and influence on HIV prevention and treatment, the different types of violence experienced by FSWs are important structural factors driving their high HIV burden.

As part of the United Nations Sustainable Development Goals, UNAIDS recently set targets to address structural factors, with a goal that less than 10% of key populations, including FSWs, experience physical or sexual violence by 2025 [12, 13]. Similarly, the World Health Organization recommended that HIV programs should work with key population-led organizations to prevent violence against key populations [14]. In Kenya, national HIV program guidelines for key populations have highlighted the need to include structural interventions since 2014. In 2017, a protocol was published to build capacity and guide local design and implementation of key population-driven violence prevention and response activities integrated with HIV prevention and treatment programs [15, 16], though there remains scope to improve implementation of services [17].

Mathematical modelling can provide insights into the dynamics of violence, its potential impact on HIV outcomes, and the likely impact of expanding coverage of services addressing violence on HIV transmission [6]. To do this, models need to dynamically represent the mechanisms through which violence affects HIV transmission, by modelling the experience of violence and their influence on associated mediators (i.e., intermediate variables such as condom use). Such models can firstly provide an understanding of the level and frequency of experience of violence, potentially helping in understanding how to reduce violence [18]. Secondly, these models can be useful to refine our understanding of what parts of the causal pathway contribute most to the HIV epidemic and thus what combinations of violence prevention and response activities (e.g., counselling) could best reduce HIV transmission [19].

However, modelling the impact of structural factors on infectious diseases is challenging. For modelling results to meaningfully inform decisions, the model needs to incorporate structural factors in an evidence-based manner, using the best data to inform the modelling of the structural factors. Stannah et al. recently reviewed existing HIV transmission dynamic models of structural factors and recommended that (i) exposure to the structural factor(s) should be explicitly defined and represented mechanistically in order to reflect variation in exposure over time and over the life course; (ii) the model should represent the key causal pathways by which the structural factors influence transmission through mediators; (iii) the strength of evidence of data sources informing the level of exposures and effect sizes of the structural factors on the mediators should be appraised; (iv) the model should be calibrated in a Bayesian framework (to reflect parameter uncertainty) to mediator- and structural factor-based outcomes as well as HIV outcomes, with additional cross-validation of these outcomes to other data where possible; (v) uncertainty and data gaps should be discussed; and finally, (vi) the specific modelling scenarios and counterfactuals used should be clearly defined [19].

The main aims of this study are twofold. Firstly, we provide a worked example of applying Stannah et al.’s recommendations [19], by using available data on HIV risk behavior and violence among FSWs from Mombasa, Kenya, to develop a transmission dynamic model of the effects of experience of violence on HIV transmission among FSWs and clients in Mombasa. As suggested by Stannah, the model represents the causal pathway for HIV risk through dynamically modelling experience of three types of violence (SV, PV, and PAA) and their associated effects on known mediators (e.g., condom use). We calibrate the model to available HIV, intervention, and violence outcomes and cross-validate predictions of the calibrated model against additional outcomes (e.g., prevalences of mediators).

Secondly, we use this calibrated model to provide insights on the dynamics of violence and its potential impact on HIV transmission among FSWs and their clients in Mombasa. Specifically, we use the model to estimate the incidence of first and recurrent occurrences of each type of violence among FSWs, to estimate the contributions of these structural factors to the HIV epidemic, and to assess the impact of potential violence-prevention interventions. Finally, we discuss the strengths and weaknesses of this analysis based on the recommendations in [19].

## Methods

### Transitions Study data

The data underlying the model parameterization comes from the Transitions Study, a cross-sectional study conducted among FSWs in Mombasa, Kenya, in 2015, described in [20]. In brief, the study recruited 408 young FSWs aged 15–24 from locations associated with sex work previously identified through systematic geographic mapping. This methodology generated a sample that was representative of young FSWs visiting sex-work hotspots, though it may have under-sampled FSWs who primarily found clients online and did not come to these spaces [20]. Participants were interviewed face-to-face to collect data on HIV risk and prevention behavior, while HIV serostatus was determined via dried blood spot testing using the Avioq HIV-1 Microelisa System (Avioq Inc., Research Triangle Park, NC, USA). Interviewees were asked about when a sex partner last hurt them (defining PV in this study), forced them to have sex when they were not willing (SV), and when they were last physically assaulted or arrested by law enforcement while working as a sex worker (PAA). Prevalences of recent and non-recent experiences of violence are derived from these data, using a 6-month period to define “recent” experience to align with other violence prevalence data from Mombasa used in cross-validation [21–23].

### Model structure

We developed a dynamic compartmental model describing HIV transmission, interventions, and experience of violence among an open, stable population of FSWs and their clients in Mombasa, Kenya. FSWs and clients are stratified by HIV infection stage and ART status, while FSWs are additionally stratified by age group (younger: < 25 years, older: 25 + years) and experience of the three types of violence (SV, PV, and PAA). New individuals enter the population as HIV-negative and sexually active, and FSWs have no previous experience of violence.

Following acute infection, untreated people living with HIV (PLHIV) progress sequentially through three stages of chronic infection (> 350, 200–350, < 200 CD4 cells/mm^3^) before AIDS-related death. HIV care is divided into three categories (ART-naïve, on ART, and dropped out, Additional File 1: Figure S1), with individuals who drop out allowed to re-initiate ART. ART is assumed to halt disease progression and reduce HIV-related mortality compared to untreated individuals in the same CD4 compartment. Modelled ART initiation rates reflect historical changes in Kenyan guidelines on eligibility criteria by CD4 [24], increases in ART initiation rates among eligible PLHIV over time for clients, and younger and older FSWs, and FSWs’ experience of violence (details below). The model also represents time trends in condom use and oral pre-exposure prophylaxis (PrEP) for FSWs.

The per-capita rate of HIV infection depends on the age-specific number of partners and number of sex acts per partnership, sexual mixing by age, the sex-specific per-act HIV transmission probability by HIV infection stage and ART status of the infected partner, condom use, and PrEP use (FSWs only) and their associated effectiveness. Sexual mixing by age is proportionate due to the need to balance sexual partnerships with one client age group. Additional details and equations are in Additional File 1: Text S3-4.

**Fig. 1:**
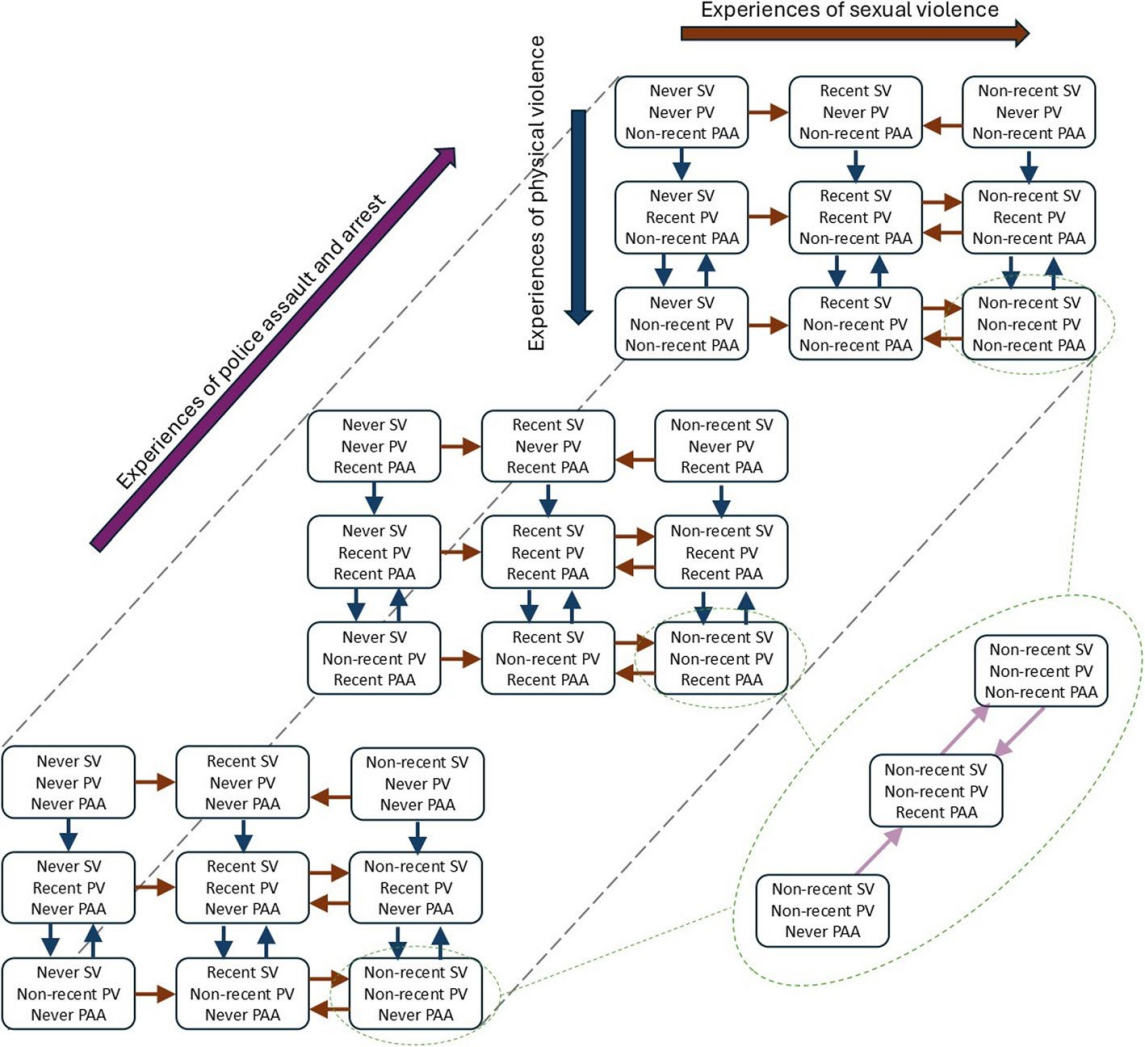
Structure of the violence component of the model. Movement horizontally corresponds to changes in the experience of sexual violence (SV), vertically corresponds to physical violence (PV), and the third dimension corresponds to police assault and arrest (PAA). The enlarged region on the right shows transitions between different PAA states for female sex workers (FSWs) who have experienced SV and PV non-recently as an example; the same pattern of transition holds for other SV and PV states. FSW can experience multiple types of violence

For FSWs, each type of violence (SV, PV, PAA) has three modelled states (Fig. 1): (i) never experienced; (ii) recent experience (within the last 6 months); and (iii) non-recent experience (more than 6 months ago). Transitions from the recent to non-recent violence state occur at a rate corresponding to an average time of 6 months; a repeat experience of violence corresponds to moving from the non-recent back to the recent violence state. The rate of experiencing a given type of violence is constant over time (Table 1), but can differ between new and recurrent experiences of violence. We assume the previous experience of one type of violence does not influence the rate of experiencing other types of violence (i.e., occur independently). The validity of this assumption is examined by cross-validating the predicted prevalence of experiencing multiple types of violence against survey data.

**Table 1:** Key parameter values and calibration data

Parameter/calibration data	Value/prior range	Source/comments
A) Violence dynamics		
Rate that FSWs experience first-time violence	Sexual violence,${\phi }_{1}$: 0.073–0.112 year^−1^ Physical violence,${\chi }_{1}$: 0.076–0.116 year^−1^ Police assault and arrest,${\psi }_{1}$: 0.146–0.217 year^−1^	Analysis of Transitions data in [25]. More details in Additional File1: Text S2
Rate that FSWs experience recurrent violence	Sexual violence,${\widetilde{\phi }}_{3}$: 0.687–1.768 year^−1^ Physical violence,${\widetilde{\chi }}_{3}$: 1.518–3.474 year^−1^ Police assault and arrest,${\widetilde{\psi }}_{3}$: 0.882–1.772 year^−1^	Analysis of Transitions data in [25]. More details in Additional File1: Text S2
Rate of transition from recent to non-recent experience of violence (for each type of violence),${\phi }_{2}={\chi }_{2}={\psi }_{2}$	2 year^−1^	Corresponds to 1/(6 months), to be consistent with time period of survey instruments used in model cross-validation [21–23]
B) HIV prevention and treatment coverage		
% of FSWs that have never experienced sexual violence who do not use condoms in partnerships (2005 onwards),${f}_{a}^{\text{no cond},\text{ now}}$	Younger FSWs: 15–20% Older FSWs: 14–25%	Younger FSWs based on Transitions data analysis [25] Older FSWs based on [21–23, 26–29]
Time when PrEP use is assumed to start,${t}_{0}^{\text{PrEP}}$	2016	[30]
PrEP use by FSWs from 2018 onwards,${f}_{a}^{\text{PrEP},\text{ now}}$	Younger FSW: 12% Older FSW: 7%	From [23] adjusting for overreporting [31]. Values consistent with those in [30]. Modelled PrEP uptake increases linearly from 2016 to 2018 (most recent data available), as shown in Additional File 1: Figure S2
Rate of HIV testing among FSWs who have never experienced violence,${\varepsilon }_{0}^{\text{FSW}}(t)$	2003–2007: 0.06 year^−1^ 2008–2012: 0.37–0.47 year^−1^ 2013 onwards: 0.80–0.92 year^−1^	2003 value from Kenya DHS female general population [32] 2008–2012 from Kenya DHS 2008–2009 report [33] and [34] 2013 onwards based on Transitions data analysis [25] and [35]; 2013 reflects the midpoint between the studies
Fraction of eligible FSWs initiating ART if diagnosed,${\pi }_{a}^{\text{FSW}}$	Younger FSWS: 10–70% Older FSWs: 50–90%	Younger FSWs from [23] and Transitions data analysis [25] Older FSWs from [35], [36], [23]
C) Influence of violence on mediators		
Risk ratio for the increase in condom non-use due to sexual violence (both recent and non-recent),${\text{RR}}_{\text{SV}}^{\text{no cond}}$	1.01–2.97	From the analysis of Transitions data [25]. Range reflects 95%CI of adjusted analysis (variables adjusted for are listed in Additional File1: Text S2)
Risk ratio for the decrease in HIV testing among FSWs due to violence (both recent and non-recent) by type of violence	Sexual violence.${\text{RR}}_{p>1}^{\text{test},\text{SV}}$: 0.82–1.01 Physical violence,${\text{RR}}_{q>1}^{\text{test},\text{PV}}$: 0.83–1.01 Police assault and arrest,${\text{RR}}_{r>1}^{\text{test},\text{PAA}}$: 0.89–1.08	From the analysis of Transitions data [25]. Range reflects 95%CI of adjusted analysis (variables adjusted for are listed in Additional File1: Text S2)
D) Calibration data		
HIV prevalence: Overall in 2005 Younger FSWs in 2015 Clients in 2005	29.2–40.0% 6.9–13.5% 5.4–24.3%	Range combined from [26, 37, 38] Transitions data [25] From [39]
ART coverage: Younger FSWs in 2015	6.8–34.5%	Transitions data [25]
Experience of SV in 2015: Younger FSWs ever experienced Younger FSWs recent experience	25.2–34.3% 8.9–15.6%	Transitions data [25]
Experience of PV in 2015: Younger FSWs ever experienced Younger FSWs recent experience	25.9–35.0% 13.6–21.2%	Transitions data [25]
Experience of PAA in 2015: Younger FSWs ever experienced Younger FSWs recent experience	40.4–50.3% 15.6–23.6%	Transitions data [25]

Parameter values and prior ranges related to (A) violence dynamics, (B) HIV prevention and treatment coverage, (C) influence of violence on mediators as well as (D) the data used in calibrating the model. Uncertainty ranges of parameters correspond to the 95% confidence intervals of empirical estimates

*FSW* female sex worker, *ART* antiretroviral therapy, *SV* sexual violence, *PV* physical violence, *PAA* police assault and arrest. Additional parameters are given in Additional File 1: Table S4

### Causal pathways assumptions

**Fig. 2:**
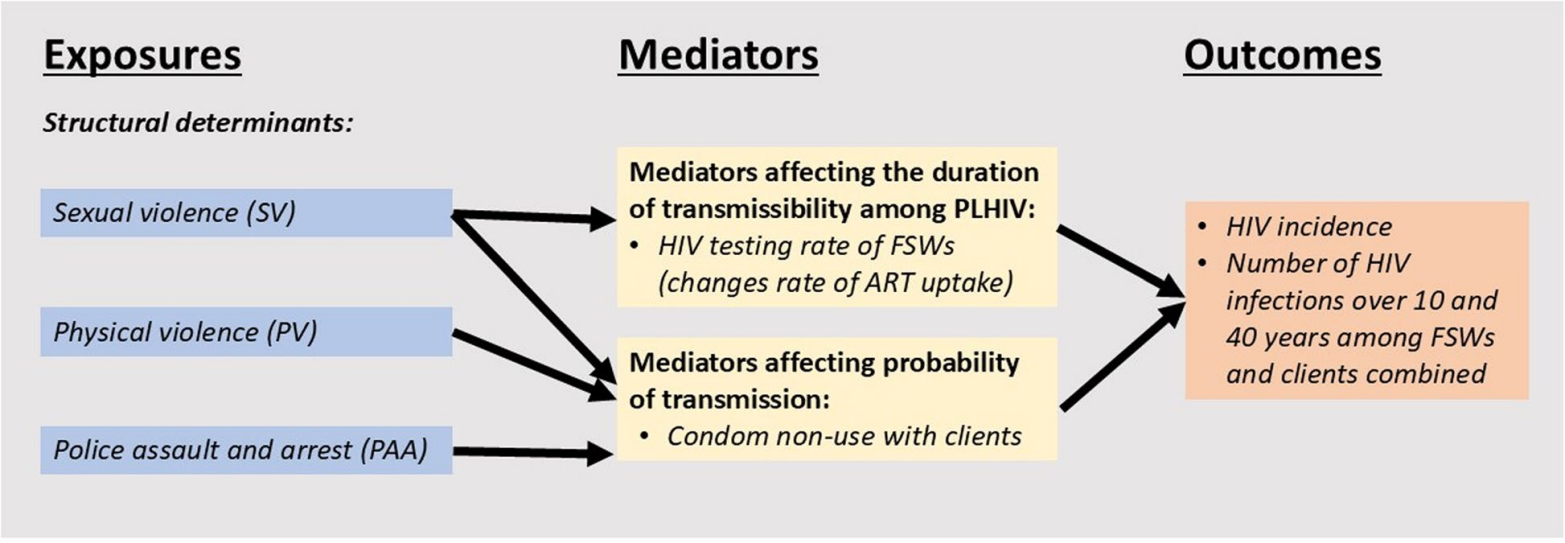
Causal pathway diagram showing how violence influences HIV outcomes via mediators. Causal pathway diagram showing how violence influences HIV outcomes via condom use and HIV testing/ART uptake in the model based on available data for FSWs in Mombasa [25]. The rate of ART uptake is shown in parentheses as a mediator because changes in HIV testing change ART uptake in the model

Figure 2 shows the assumed model causal pathways based on the analysis of cross-sectional data from the Transitions Study, which identified two mediators from eight potential mediators (listed in Additional File 1: Text S2) [25]. In this adjusted analysis, each type of lifetime (encompassing both recent and non-recent) experience of violence was negatively associated with having an HIV test in the past year, whereas only experience of lifetime sexual violence was positively associated with condom non-use. Thus, to reflect their influence on HIV in the model, we first multiply ART initiation rates (the product of HIV testing rate and the fraction of treatment-eligible FSWs following diagnosis), for FSWs living with HIV in both recent and non-recent violence compartments, by the empirical risk ratio estimates of HIV testing between FSWs experiencing and not experiencing the specific type of violence. Second, for FSWs who have ever experienced sexual violence in the model, we multiply the fraction of partnerships without condoms by the empirical risk ratio estimates [25]. Given the low PrEP uptake among FSWs in Kenya [23], and the lack of current site-specific quantitative evidence on the influence of violence on PrEP use among FSWs (see Additional File: Text S3 for further information), we ignore any effects of violence on PrEP use.

For FSWs experiencing more than one type of violence, we multiply their rate of ART initiation by the smallest multiplier (instead of assuming a multiplicative effect) across the different types of violence experienced. This corresponds to a conservative assumption about the maximum impact of structural interventions and is explored in the sensitivity analysis described later.

### Model parameterization and initialization

Table 1 shows key prior parameter ranges, calibration data, and data sources related to violence dynamics, HIV prevention and treatment coverage, and the influence of violence on the two mediators identified. Additional parameters are in Additional File 1: Table S4.

Given the lack of longitudinal studies among FSWs in Mombasa/Kenya, parameters and outcomes related to violence, sexual behavior, and HIV prevention and treatment are preferentially derived from the site-specific Transitions Study (additional details in Additional File1: Text S2). Given the lack of data for older FSWs, we assume the same prior rates as for young FSWs. The risk ratios for violence on condom use and ART initiation (due to reduced HIV testing) in the model are also derived from Transitions data, increasing the internal validity of estimates. Information on condom use and HIV testing in FSWs at other timepoints and for older FSWs [21–23, 26–29], and client behavior [24, 40], were sourced from additional published literature from Kenya. Key parameters are assigned prior uncertainty ranges corresponding to the 95%CIs of empirical estimates.

HIV was introduced in 1970 when violence was already at equilibrium. The model is written in the C programming language, using a fourth-order Runge–Kutta solver. All model output analysis is carried out in R version 4.3.2 [41] (details in Additional File 1: Text S6).

### Model fitting and validation

The model is fitted in a Bayesian framework [42]. Latin hypercube sampling is used to generate 20,000 parameter sets, sampled from uniform prior parameter ranges [43]. A parameter set is selected as a good fit if the model predictions all lie within the 95%CIs of the following empirical outcome estimates (see Table 1 for details): 2005 overall HIV prevalence in FSWs and in clients, 2015 HIV prevalence in younger FSWs, ART coverage in younger FSWs in 2015, and prevalences of both recent and lifetime experience of SV/PV/PAA in younger FSWs in 2015. The resulting posterior parameter set is used to simulate the baseline scenario.

Additional estimates at different timepoints or age groups from data sources not used in fitting are used to cross-validate model predictions. This includes HIV prevalence [26, 35, 44–48], recent (last 6 months) experience of SV and PAA in older FSWs (not used to fit, given the different interview method and sampling frame) [21–23], condom use in younger and older FSWs [21–23, 26, 29, 49], and ART coverage [23, 35] (Additional File 2: Tables S5-8).

Apart from the client ART coverage and client HIV prevalence which are based on national estimates from DHS surveys, KenPHIA, and UNAIDS (Additional File 2:Tables S5-6) [24, 50, 51], all other cross-validation data is specific to Mombasa. Finally, we cross-validate model prevalence estimates of different combinations of types of violence against data from the Transitions Study to validate our assumption that each type of violence is independent.

### Model plan of analysis

Using the baseline parameter set, we conduct three main analyses.

#### Incidence of violence among FSW

For each type of violence, we estimate the incidence of violence (in person-years) for women who have never experienced violence (“first violence”), and in only FSW who have experienced violence but not recently (“recurrent violence”). We also estimate the incidence rate ratio of recurrent compared to first violence in 2023.

#### Contribution of violence, mediated through ART and condom use, to new HIV Infections among FSWs and clients

We estimate the transmission population-attributable fraction (tPAF) [52, 53], the relative difference in the cumulative number of new infections over 10 and 40 years (from 2023), between the baseline scenario and a matched counterfactual scenario where the influence of a specific violence on certain mediators is removed. We derive tPAFs for eight scenarios (Table 2A).

**Table 2:** Counterfactual scenarios to estimate the transmission population-attributable fraction *(*tPAF*)*(A) and intervention impact (B)

Counterfactual scenario description	Scenario name (A) SF—mediator (B) SF—mediator; rate	Risk ratio for effect of violence on mediator^a^								Rate of future violence^b^					
		**Condom non-use**		**HIV testing**											
		***SV***		***SV***		***PV***		***PAA***		***SV***		***PV***		***PAA***	
		**Recent**	**Non-recent**	**Recent**	**Non-recent**	**Recent**	**Non-recent**	**Recent**	**Non-recent**	**New**	**Recurrent**	**New**	**Recurrent**	**New**	**Recurrent**
(A) tPAF scenarios															
tPAF from SV on condom use	SV**—**condom	×	×												
tPAF from violence on HIV testing	All violence**—**testing			×	×	×	×	×	×						
tPAF from recent violence	Recent violence**—**all	×		×		×		×							
tPAF from non-recent violence	Non-recent violence**—**all		×		×		×		×						
tPAF from any type of violence	All violence**—**all	×	×	×	×	×	×	×	×						
tPAF from SV	SV**—**all	×	×	×	×										
tPAF from PV	PV**—**all					×	×								
tPAF from PAA	PAA – all							×	×						
(B) intervention scenarios															
Prevent *X*% of future violence and mitigate *X*% of effects of past violence	IntX: all violence—all mediators; rates	×	×	×	×	×	×	×	×	×	×	×	×	×	×
Prevent *X*% of future violence	IntX: all violence—no mediators; rates									×	×	×	×	×	×
Prevent *X*% of future violence and mitigate *X*% of recently experienced violence	IntX: all violence—recent mediators; rates	×		×		×		×		×	×	×	×	×	×
Prevent *X*% of future violence and mitigate *X*% of non-recently experienced violence	IntX: all violence—non-recent mediators; rates		×		×		×		×	×	×	×	×	×	×
Prevent 100% of future sexual violence (SV) and mitigate 100% of the effects of past SV	Int100: SV—all mediators; SV rate	×	×	×	×					×	×				
Prevent 100% of future physical violence (PV) and mitigate 100% of the effects of past PV	Int100: PV—testing; PV rate					×	×					×	×		
Prevent 100% of future police assault and arrest (PAA) and mitigate 100% of the effects of past PAA	Int100: PAA—testing; PAA rate							×	×					×	×

The table shows the corresponding risk ratios and violence rates modified in each counterfactual scenario (shown in rows). A cross denotes that the corresponding risk ratio/rate is modified in the given counterfactual scenario; otherwise, it maintains the same value as in the matched baseline run

^a^When marked with an “X”, risk ratios are set to 1 from 2023 (for tPAF and *X* = 100% intervention scenarios) or (1+*R*)/2, where *R* was the original risk ratio (for *X* = 50% intervention scenarios)

^b^When marked with an “*X*”, rates are set to 1 from 2023 (for tPAF and *X* = 100% intervention scenarios) or halved (for *X* = 50% intervention scenarios)

#### Impact of potential violence interventions on cumulative new HIV infections

We model two main types of violence interventions, starting in 2023, addressing (i) future experience (reducing or stopping violence), through reducing violence rates, and (ii) additionally mitigating long-term effects of past violent experience (for example through counselling or peer support) by also modifying the corresponding risk ratios affecting the mediators. Table 2B shows the scenarios considered. We consider interventions that reduce rates or the effects on mediators by *X* = 50% and *X* = 100%. We assess the impact of interventions addressing all violence types or each type separately. For all violence, we also consider interventions addressing the influence of all past experiences, recent experiences only, and non-recent experience only, corresponding to interventions prioritizing different groups of FSWs. Impact is measured as the percentage of new infections averted over 10 and 40 years by the intervention, relative to the baseline scenario, in FSWs and clients combined.

For all outcomes, we present the median and 95% credible interval (95% CrI) across fitted runs.

### Sensitivity analysis

Using the posterior parameter set, we assess correlations between each varied parameter and (i) the 10-year tPAF for the “All violence—all” scenario and (ii) the 10-year impact of the violence intervention “Int100: all violence—all mediators; rates”. We show scatter plots for all parameters with correlation coefficient > 0.2.

We finally examine the sensitivity of the results to the assumption described in the “Causal pathways assumptions” section above, regarding how the risk ratios combine when FSWs experience multiple types of violence (see Additional File 2: Text S8 for details).

## Results

### Calibration and cross-validation

**Fig. 3:**
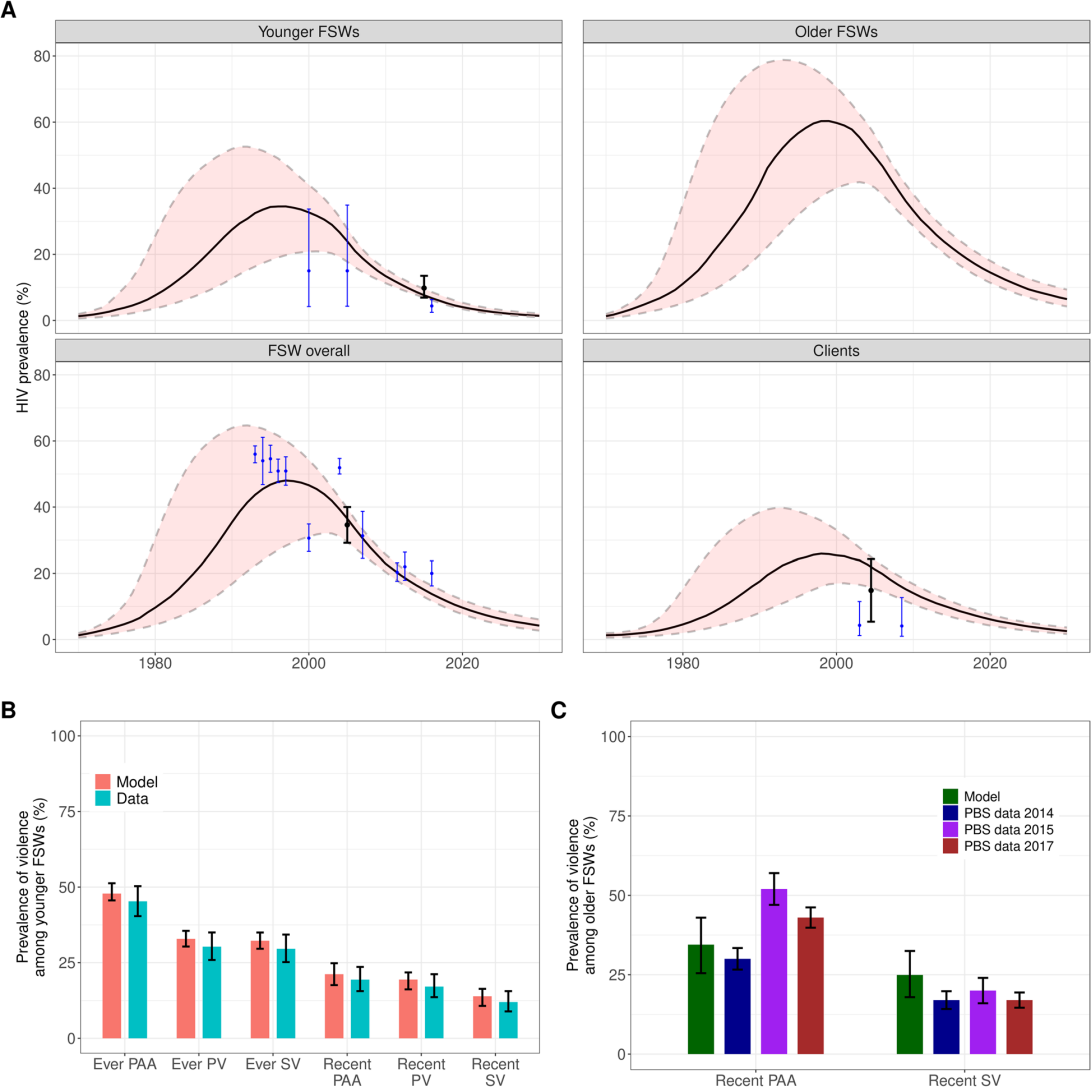
Model calibration results. Panel **A** shows predicted HIV prevalence trends by population group (line = median, shaded area = 95%CrI) compared to 95%CI of empirical estimates (black error bars show data used in calibration, blue error bars show cross-validation data). FSW overall combines younger and older FSW. Panel **B** shows predicted prevalence (median as bar and 95%CrI as error bars) of ever and recent experience of each type of violence in younger FSWs compared to empirical prevalence estimates from Transitions data in 2015 (error bars represent 95%CI) used in calibration. Panel **C** shows predicted prevalence (median as bar and 95%CrI as error bars) of recent experience of PAA and SV in 2015 compared to empirical prevalence estimates from Mombasa polling booth surveys (PBS) in 2014, 2015, and 2017 (error bars represent 95%CI) for cross-validation. FSW female sex worker, SV sexual violence, PV physical violence, PAA police assault and arrest, CrI credible interval, CI confidence interval

We identified 57 parameter sets that produced model runs which fitted the calibration data on HIV prevalence (Fig. 3A) and ART coverage (Additional File 2: Figure S3) among FSW and clients and the prevalence of experience of each type of violence in younger FSWs (Fig. 3B).

The calibrated model produced predictions of HIV prevalence, ART coverage, condom use, and prevalence of experience of violence consistent with the cross-validation data not used in calibration (Fig. 3A and C, Additional File 2: Figures S3-7). Importantly, the model reproduced well the cross-validation estimates of the prevalence of recent SV and recent PAA in older FSWs, which, given the lack of setting-specific data for older FSWs, were informed by the same violence-related parameters as younger FSWs (Fig. 3C).

### Incidence of violence among FSW

Among FSWs who have never previously experienced violence, the predicted incidence of first violence in 2023 was highest for PAA (median (95%CrI): 0.20 (0.17–0.22) per person-year, ppy), about twice that of SV and PV (0.10 (0.09–0.11), 0.11 (0.09–0.12) ppy, respectively) (Additional File 2:Figure S8).

The predicted recurrence of violence among FSWs was extremely common. Recurrent PV was most common (2.65 (1.82–3.37) ppy) followed by PAA (1.37 (0.94–1.74)) and SV (1.26 (0.80–1.67)). Recurrence of PV, SV, and PAA were 24.32 (16.07–34.46), 11.69 (7.95–17.43), and 6.79 (4.80–9.30) times more frequent for SV and PAA respectively than the corresponding incidence of first violence (results not shown).

### Contribution of violence to new HIV infections (tPAFs)

**Fig. 4:**
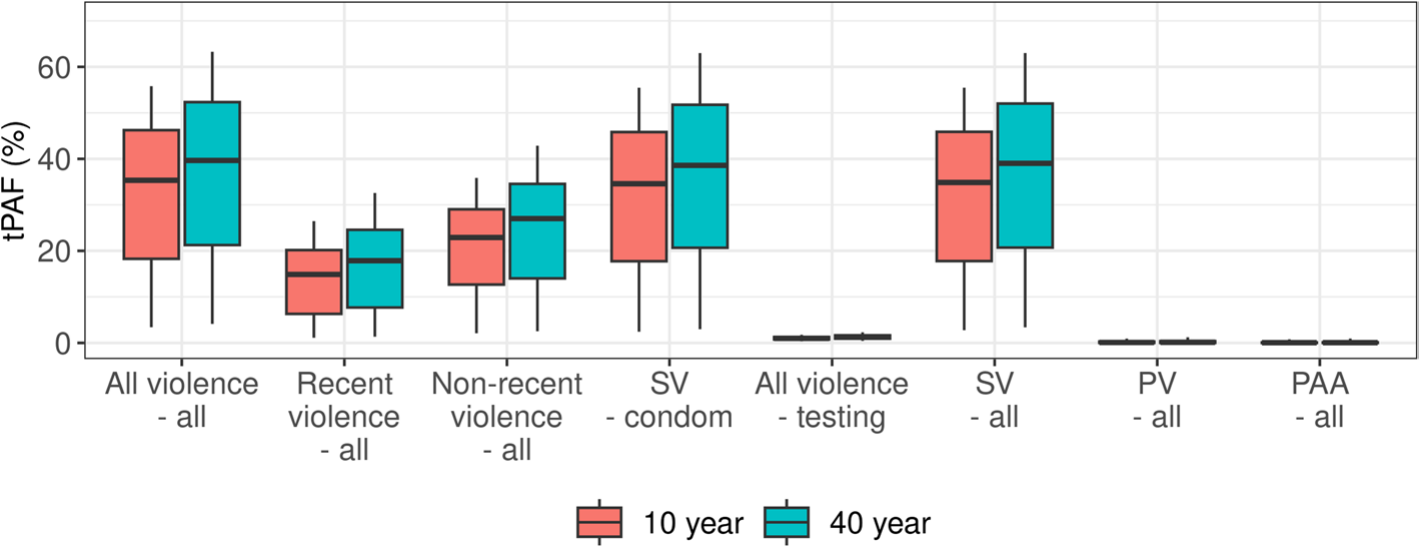
Contribution of violence to HIV transmission: transmission population-attributable fractions (tPAFs) in female sex workers (FSWs) and clients combined over 10 and 40 years from 2023. Central lines show median model estimates, boxes show interquartile range, and error bars show 95% credible intervals. tPAF scenario labels are described in Table 2

The median tPAF is 35.3% (95%CrI 3.4–55.8%) of new HIV infections in FSWs and clients combined over 10 years and 39.7% (4.1–63.3%) over 40 years, from all violence combined through their effects on both mediators (Fig. 4). The largest contribution is from the effects of SV on condom use (median tPAF = 34.6% (2.4–55.5%) over 10 years, increasing to 38.6% (3.0–63.0%) over 40 years). The tPAFs of PV and PAA are small (medians 0.9% and 0.4% respectively over 10 years). Although all types of violence reduced HIV testing through the modelled causal pathway, the influence of violence on HIV testing contributes little (median tPAF: 1.0% over 10 years). A larger fraction of infections is attributable to non-recent than recent experience of violence (median tPAFs: 22.9% and 14.9% over 10 years, respectively) given the larger fraction of FSWs who have non-recent experience of violence, particularly sexual violence (Fig. 3B and C) and the persistent negative effect on condom use. Among FSWs and clients respectively, 27.9% (2.1–47.0%) and 39.8% (4.1–59.0%) of all new HIV infections could be attributable to violence over 10 years (Additional File 2: Figures S12-13). The larger impact on clients is due to the larger increase in condom use in older FSWs, which have the higher HIV prevalence (Fig. 3A), than younger FSWs (Additional File 2: Figure S11).

The small overall tPAF from the effects of violence on HIV testing is due to the low percentage of FSWs living with HIV who are ART-naïve (median 15.2% in 2023, Additional File 2: Figure S9) and the small influence of violence on HIV testing/ART uptake, meaning that the increase in ART coverage is small (Additional File 2: Figure S10). The increase in condom use in the absence of effects from violence compared to the baseline scenario is conversely larger (Additional File 2: Figure S11). Additionally, increasing ART coverage among FSWs only directly benefits clients (also contributing to the larger tPAF in clients than FSWs, Additional File 2: Figures S12-13).

### Impact of violence interventions

**Fig. 5:**
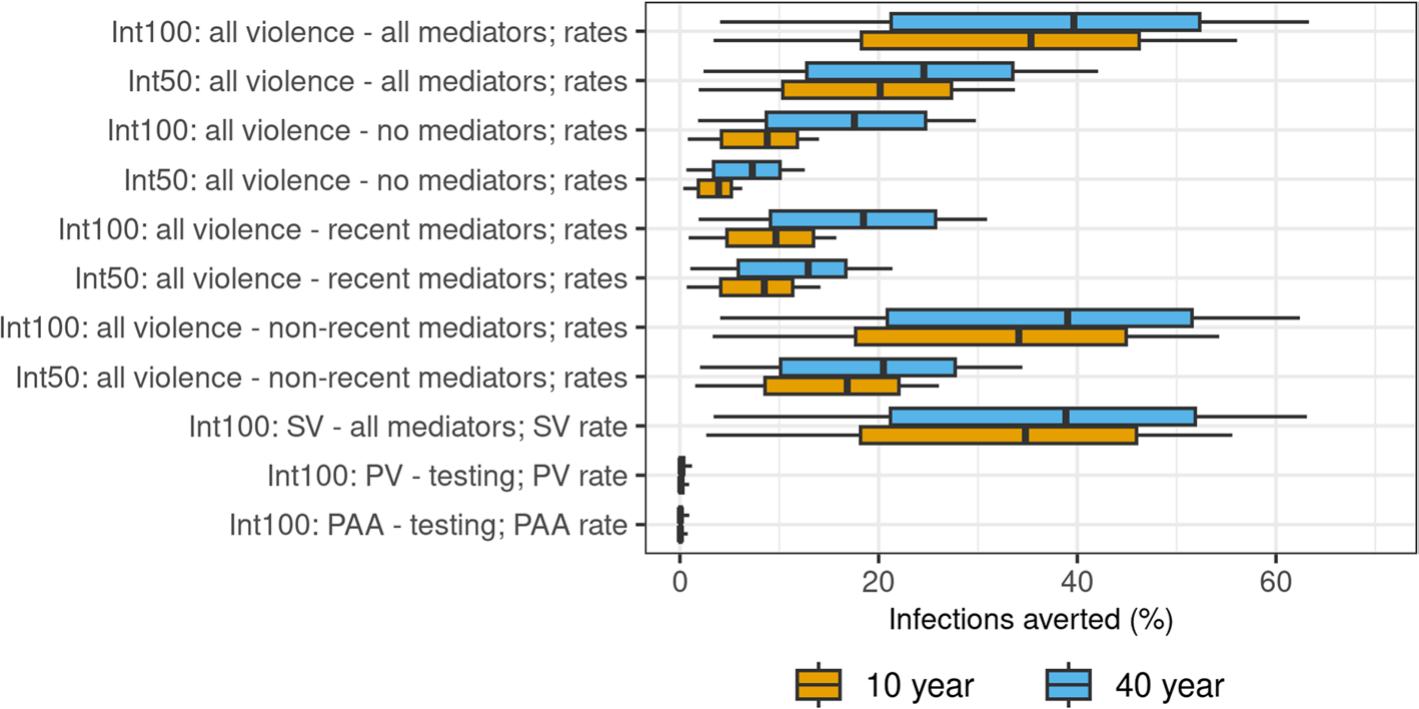
Impact of violence intervention. Percentage of infections averted in female sex workers (FSWs) and clients combined by different interventions compared to the baseline scenario over 10 and 40 years from 2023. Unless indicated, the experience of past violence includes all types of violence. Central lines show median model estimates, boxes show interquartile range, and error bars show 95% credible intervals. Intervention scenarios are described fully in Table 2

Interventions that prevent 50% and 100% of future experience of violence (without mitigating effects of past violence) may only avert a median of 3.9% (95%CrI: 0.3–6.3%) and 8.8% (0.8–14.0%) infections in FSWs and clients over the next 10 years, respectively, and 7.3% (0.7–12.6%) and 17.6% (1.8–29.8%) over 40 years, mainly due to the reduced rates of SV experience (Fig. 5).

However, interventions that also mitigate 50% or 100% of the effects of past violence on the mediators could be more impactful than those that only reduce future experience of violence, especially in the shorter term, averting 20.2% (1.9–33.2%) and 35.3% (95%CrI 3.4–55.8%) (same as the 10-year tPAF) over 10 years, respectively. This is because (as shown in the tPAF analysis) the negative impact on HIV is mainly due to the effects of non-recent violence, which are only slowly reduced over time when only preventing future experiences as it takes time for the prevalence of non-recent experience of violence to decline. For example, it takes about 11 years for the prevalence of non-recent SV to reduce by half (Additional File 2: Figure S14). Intervention impacts in FSWs and clients separately are shown in Additional File 2: Figs. 15–16.

As in the tPAF analysis, the impact of only addressing PV and PAA is predicted to be small in this setting. Finally, it is important to note that a 50% effective intervention (reducing future violence by 50% and reducing the effects of past violence by 50%) is not negligible, with an impact of around half that of the 100% intervention.

There is substantial uncertainty in both tPAF and intervention impact estimates (Figs. 4–5). Our sensitivity analysis suggests that most of the uncertainty in both estimates is due to the uncertainty in the risk ratio of condom use from SV experience and the effectiveness of condoms (Additional File 2: Figure S17-18). However, neither tPAF nor impact estimates are sensitive to the assumption regarding how the risk ratios combine when FSWs experience multiple forms of violence (Additional File 2: Figures S19-20).

## Discussion

In this study, we carried out and described a mathematical modelling analysis that examined the effects of violence against FSWs by clients, police, and other perpetrators on HIV transmission among FSWs in Mombasa, Kenya, following the recent recommendations of [19] on modelling structural factors for HIV. In this setting, violence is extremely common. Each year, an estimated 20% of FSWs experience police assault and arrest (PAA) for the first time, while 10% experience physical violence (PV) and sexual violence (SV). Moreover, once a FSW has experienced a type of violence, they are 7–24 times more likely to experience it again, on average once (SV and PAA) or over twice (PV) a year.

In this setting, lifetime experience of SV contributes the most to the HIV epidemic among FSWs and their clients, mainly through its impact on condom use. Over a third of new infections in FSWs and clients over the next 10 years may be due to lifetime SV, albeit with considerable uncertainty in estimates. The effects of PV and PAA are negligible in this setting (tPAF = 1% over 10 years).

Our analysis revealed that structural interventions that only tackle future experience of violence will likely have a moderate effect, at least in the short to medium term. Indeed, completely preventing future violence would only avert a tenth of future infections among FSWs and clients given the slow decline over time of the prevalence of lifetime violence. Our results highlight that to be fully and more rapidly impactful, interventions should also seek to mitigate the associated persistent negative effects resulting from experiencing violence in a lifetime. For example, in Kenya, the registration process for new FSWs enrolling in programs has been modified [54] to ask about past experiences of violence, with additional counselling and psycho-social support services [55] as well as more frequent follow-up for all FSWs with past experience of violence, whether recent or not.

A recent systematic review of gender-based violence among key populations found no empirical studies assessing the impact of FSW violence interventions on HIV prevalence or other biological endpoints [56], while there are only four studies using dynamical modelling to evaluating the impact of structural factors among FSWs [19]. For example, Decker et al. [57] estimated that reducing the prevalence of violence among FSWs in Kenya from 32 to 2% would reduce HIV incidence among FSWs by a quarter over 2011–2016. Wirtz et al. estimated that scaling up community empowerment interventions may reduce cumulative HIV infections among FSWs in Kenya by 12% over 2011–2016 [58]. Vassall et al. showed that, as part of a large-scale HIV intervention among FSWs in India, the community mobilization and empowerment component including violence reduction contributed 31–39% of the total estimated intervention impact during the period 2004–2011 [59]. Finally, Shannon et al., which were the only modelling study to dynamically simulate exposure to violence, estimated that eliminating sexual violence in Kenya, including negative effects of recent past experience, would avert 17% of HIV infections in FSWs and clients over 10 years [6].

Our results should be interpreted in light of certain considerations. First, modelling the impact of structural factors on infectious diseases such as HIV is challenging, partly due to data gaps [19, 60]. In this study, the estimates of the experience of violence and its influence on the mediators in the causal pathway were based on data from a cross-sectional study [20], which makes it difficult to disentangle temporality of the effects of violence on the mediators and provide weaker evidence than if based on longitudinal data [19]. Further, our tPAF and intervention impact estimates were very uncertain due to the wide confidence intervals of the estimates of the association between violence and the mediators, in particular the risk ratio for condom non-use from experiencing SV. This highlights the need for additional estimates of the magnitude of association from longitudinal data to improve validity and precision. We also assumed that interventions to mitigate the associated persistent negative effects resulting from experiencing past violence would immediately remove these effects entirely, though evidence from programs for this is currently lacking, and further research should aim to better understand the impact of such interventions among FSWs. Second, we were unable to derive different estimates of the effects of recent and non-recent violence on the mediators as this would result in even wider uncertainty bounds. It is plausible that the estimated magnitude of association between the long-term experience of violence and mediators would differ from those of recent violence. However, given the higher prevalence of non-recent experience compared to recent experience, it is unlikely to drastically change our conclusions. Third, in our causal pathway, we looked at how violence affects HIV testing rather than ART coverage itself. It is possible that ART uptake is influenced differently by violence than HIV testing, again emphasizing a data gap. There is also a lack of data on PrEP use among FSWs, and the effects of violence on PrEP more broadly, which we therefore were unable to model. While we were unable to distinguish perpetrators based on the survey questions, effects may differ by perpetrator [61]. Studies in other settings or focusing on specific FSW typologies, such as venue-based FSWs, are needed to provide external validity for the findings in the present study. Finally, the model only captured violence and HIV transmission, excluding other consequences such as reduced access to general health services and adverse mental health outcomes [62, 63].

Our modelling analysis also has several strengths which are consistent with recent recommendations (Table 3). We have mechanistically and dynamically represented exposure to more than one type of structural factor (i.e., violence) and the associated causal pathways for HIV transmission through mediators (condom use and HIV testing) [19]. While the underlying data was cross-sectional, the use of a local data source ensured that the same exposure definition was used to empirically estimate the prevalence of the structural factors and their adjusted effects on mediators, thereby increasing internal consistency. Importantly, we have successfully calibrated the model to not only HIV outcomes but also violence outcomes and cross-validated predictions of the calibrated model against multiple additional empirical estimates of outcomes not used at the fitting stage, including prevalences of the mediators. Finally, representing exposure to the structural factor more granularly (i.e., recent and non-recent experiences of violence) not only allowed estimation of the incidence of recurrent episodes of violence but also an exploration of a wider range of interventions with different mechanisms (e.g., exposure reduction versus post-exposure counselling).

**Table 3:** How recommendations for modelling structural factors in HIV are implemented in this analysis Table adapted from Fig. 3 in [19]

No	Recommendation	How considered in our analysis
1	Represent exposure to SFs granularly (e.g., frequency, duration intensity, multiple SFs)	Three SFs represented: SV, PV, and PAA, with recent and non-recent experience
2	Reflect the main causal pathways (include main mediators and relationships between SFs)	Two mediators represented, based on an analysis that explored additional potential mediators. Assumed independence of exposure to different SFs validated against empirical data
3	Informed by appropriate, well-estimated SF parameters (e.g., using setting- and population-specific data on prevalence, incidence, and effects of SFs, ideally based on the same exposure definitions and categories. Consider dose–response, effect modification, interactions)	SF and causal pathway parameters, adjusted for confounding, informed by the same population and site-specific data sources, using the same exposure definitions
4	Calibrated using relevant epidemiological and structural estimates (calibrated using relevant epidemiological data stratified by relevant exposure categories and data on structural factor prevalence; fitting method accounts for uncertainty in parameter assumptions)	Model fitted within a Bayesian framework accounting for uncertainty in HIV epidemiological, intervention, and SF parameter assumptions with ranges informed as described above and clearly specified in Table 1 and Additional File 1: Table S4. Fitting outcomes include HIV trends, levels of intervention, and SF prevalences
5	Use a calibrated model to predict the impact of SFs (predict impacts on future exposure, mediators, and HIV outcomes based on well-defined counterfactual scenarios, validated against empirical SF estimates)	Predictions from the calibrated model of HIV prevalence and ART coverage, as well as SF prevalences, and mediators cross-validated using data not used for fitting. Counterfactuals to derive the tPAF and intervention impact clearly defined (Table 2). Importantly the calibration allowed estimation of the incidence of first and recurrent episodes of the different types of violence over 6 months
6	Future data needs (data gaps identified)	Model assumptions based on population and site-specific cross-sectional data among young FSWs with a carefully documented history of experience of violence. However, sample size is too small to explore the influence of more refined exposure definitions on mediators than currently considered (e.g., dose–response relationships, recent vs non-recent experience). The cross-sectional nature of the data precludes making a strong causality statement about the association between violence and mediators. Sensitivity analysis identified the need for more data to improve the precision of the estimates of the causal pathways

*SF* structural factor, *SV* sexual violence, *PV* physical violence, *PAA* police assault and arrest, *tPAF* transmission population-attributable fraction, *ART* antiretroviral therapy

## Conclusions

This study shows the frequent experiences of violence of female sex workers in Mombasa, Kenya, and the particular contribution of sexual violence to the HIV epidemic in this setting, highlighting the work needed to meet the goals of reducing violence in key populations and its impact on HIV [12]. Our work also emphasizes that interventions that only reduce future experience of violence without mitigating the persistent negative effects of past experience of violence on FSWs will not have the greatest impact. However, given the wide uncertainty of model predictions, our analysis also highlights key data gaps, particularly around the importance of improving estimates of the effects of sexual violence on mediators. More generally, there is a need for longitudinal studies and more formal mediation causal pathway analysis to strengthen the evidence of the role of structural factors on HIV transmission. Modelling of structural factors on infectious diseases is in its infancy. However, we found that the recent set of recommendations for modelling structural factors are useful to help describe the model and to highlight strengths, weaknesses, and data gaps.

## Supplementary Information

Additional file 1: Text S1-S6; Figures S1-S2; Tables S1-S4. Text S1 – Study setting. Text S2 – Description of underlying statistical analysis. Text S3 – Model details. Text S4 – Model equations. Text S5 – Model parameters. Text S6 – Model outputs, description of sensitivity analyses and additional details. Figure S1 – Model natural history of HIV and care cascade. Figure S2 – Model PrEP uptake over time. Table S1 – Model indices. Table S2 – Model state variables. Table S3 – Modelled changes in ART eligibility. Table S4– Model parameters.

Additional file 2: Text S7-8; Figures S3-S20. Tables S5-S10.  Text S7 – Model calibration and cross-validation. Text S8 – Additional results. Figures S3 and S4 – Model calibration and cross-validation to ART coverage. Figure S5 – Model cross-validation to condom use. Figure S6 – Model cross-validation to violence exposure. Figure S7 – Modelled prevalence of violence. Figure S8 – Modelled incidence of violence. Figure S9 – modelled percentage of ART-naïve people living with HIV. Figure S10 – comparison of ART coverage between scenarios. Figure S11- comparison of condom use between scenarios. Figure S12 and S13 – tPAF in FSWs and clients. Figure S14 – Prevalence of violence over time with/without intervention. Figure S15 and S16– impact of violence intervention for FSWs and clients. Figure S17 and S18 – scatter plots showing correlation between 10 year tPAF/10 year intervention impact and corresponding parameter values. Figures S19 and S20 – Sensitivity analysis results. Table S5 – HIV prevalence cross-validation data. Table S6 – ART coverage cross-validation data. Table S7 – condom use cross-validation data. Table S8 – Older FSW violence cross-validation data.

## Data Availability

No research data was used outside the submitted manuscript file.

## Abbreviations

95%CI: 95% Confidence interval
95%CrI: 95% Credible interval
ART: Antiretroviral therapy
FSW: Female sex worker
PAA: Police assault and arrest
PBS: Polling booth survey
PrEP: Pre-exposure prophylaxis
PV: Physical violence
ppy: Per person-year
SF: Structural factor
SV: Sexual violence
tPAF: Transmission population-attributable fraction

## Data availability

No datasets were generated or analysed during the current study.

## References

[1] Deering KN, Amin A, Shoveller J, Nesbitt A, Garcia-Moreno C, Duff P, et al. A systematic review of the correlates of violence against sex workers. Am J Public Health. 2014;104(5):e42-54. 24625169 10.2105/AJPH.2014.301909 PMC3987574

[2] Shannon K, Csete J. Violence, condom negotiation, and HIV/STI risk among sex workers. JAMA. 2010;304(5):573–4. 20682941 10.1001/jama.2010.1090

[3] Leis M, McDermott M, Koziarz A, Szadkowski L, Kariri A, Beattie TS, et al. Intimate partner and client-perpetrated violence are associated with reduced HIV pre-exposure prophylaxis (PrEP) uptake, depression and generalized anxiety in a cross-sectional study of female sex workers from Nairobi, Kenya. J Int AIDS Soc. 2021;24(Suppl 2):e25711. 34164924 10.1002/jia2.25711 PMC8222843

[4] Beattie TS, Bhattacharjee P, Ramesh BM, Gurnani V, Anthony J, Isac S, et al. Violence against female sex workers in Karnataka state, south India: impact on health, and reductions in violence following an intervention program. BMC Public Health. 2010;10: 476. 20701791 10.1186/1471-2458-10-476 PMC2931467

[5] Leddy AM, Weiss E, Yam E, Pulerwitz J. Gender-based violence and engagement in biomedical HIV prevention, care and treatment: a scoping review. BMC Public Health. 2019;19(1):897. 31286914 10.1186/s12889-019-7192-4 PMC6615289

[6] Shannon K, Strathdee SA, Goldenberg SM, Duff P, Mwangi P, Rusakova M, et al. Global epidemiology of HIV among female sex workers: influence of structural determinants. Lancet. 2015;385(9962):55–71. 25059947 10.1016/S0140-6736(14)60931-4 PMC4297548

[7] Tounkara FK, Diabaté S, Guédou FA, Ahoussinou C, Kintin F, Zannou DM, et al. Violence, condom breakage, and HIV infection among female sex workers in Benin. West Africa Sex Transm Dis. 2014;41(5):312–8. 24722385 10.1097/OLQ.0000000000000114 PMC4000255

[8] El-Bassel N, Mukherjee TI, Stoicescu C, Starbird LE, Stockman JK, Frye V, Gilbert L. Intertwined epidemics: progress, gaps, and opportunities to address intimate partner violence and HIV among key populations of women. Lancet HIV. 2022;9(3):e202–13. 35151376 10.1016/S2352-3018(21)00325-8 PMC10009883

[9] Mendoza C, Barrington C, Donastorg Y, Perez M, Fleming PJ, Decker MR, Kerrigan D. Violence from a sexual partner is significantly associated with poor HIV care and treatment outcomes among female sex workers in the Dominican Republic. JAIDS Journal of Acquired Immune Deficiency Syndromes. 2017;74(3):273–8. 27861234 10.1097/QAI.0000000000001250

[10] Oldenburg CE, Ortblad KF, Chanda MM, Mwale M, Chongo S, Kanchele C, et al. Brief report: intimate partner violence and antiretroviral therapy initiation among female sex workers newly diagnosed with HIV in Zambia: a prospective study. J Acquir Immune Defic Syndr. 2018;79(4):435–9. 30142141 10.1097/QAI.0000000000001841 PMC6203637

[11] Decker MR, Crago AL, Chu SK, Sherman SG, Seshu MS, Buthelezi K, et al. Human rights violations against sex workers: burden and effect on HIV. Lancet. 2015;385(9963):186–99. 25059943 10.1016/S0140-6736(14)60800-X PMC4454473

[12] UNAIDS. 2025 UNAIDS targets. Available from: https://aidstargets2025.unaids.org/.

[13] UNAIDS. AIDS and the sustainable development goals.

[14] World Health Organization. Consolidated guidelines on HIV prevention, diagnosis, treatment and care for key populations. Geneva: World Health Organization; 2014. 25996019

[15] National AIDS & STI Control Programme MoH. National guidelines for HIV/STI programming with key populations. 2014.

[16] National AIDS & STI Control Programme MoH. National violence prevention and response protocol: a means to enhancing HIV prevention amongst key populations. 2017.

[17] Hendriks S, Woensdregt L. Sex Work & Violence in Kenya: A participatory research. AIDS Fonds. 2020:48. 10.13140/RG.2.2.36024.37128.

[18] Walker J, Elmes J, Grenfell P, Eastham J, Hill K, Stuart R, et al. The impact of policing and homelessness on violence experienced by women who sell sex in London: a modelling study. Scientific Reports (accepted). 10.1038/s41598-023-44663-w PMC11002010 38589373

[19] Stannah J, Flores Anato JL, Pickles M, et al. From conceptualising to modelling structural determinants and interventions in HIV transmission dynamics models: a scoping review and methodological framework for evidence-based analyses. BMC Med. 2024;22:404. 10.1186/s12916-024-03580-z. 10.1186/s12916-024-03580-z PMC11414142 39300441

[20] Cheuk E, Mishra S, Balakireva O, Musyoki H, Isac S, Pavlova D, et al. Transitions: novel study methods to understand early HIV risk among adolescent girls and young women in Mombasa, Kenya, and Dnipro, Ukraine. Front Reprod Health. 2020;2(7). 10.3389/frph.2020.00007 PMC9580775 36304700

[21] National STI/AIDS Control Programme MoH, Kenya. National behavioral assessment of key populations in Kenya polling booth survey report. Nairobi, Kenya: NASCOP; 2014.

[22] National AIDS & STI Control Programme MoH, Kenya. Second national behavioural assessment of key populations in Kenya: polling booth survey report. Nairobi: NASCOP; 2016.

[23] National AIDS & STI Control Programme MoH. Third national behavioural assessment of key populations in Kenya: polling booth survey report. Nairobi: NASCOP; 2018.

[24] National AIDS & STI Control Programme MoH. Kenya HIV estimates. Nairobi: NASCOP; 2018.

[25] Mountain E. HIV risk and prevention among sex workers: a focus on structural determinants and interventions [PhD thesis]. Imperial College London; 2017. http://hdl.handle.net/10044/1/78478. Accessed 16 Jan 2024.

[26] Luchters S, Chersich MF, Rinyiru A, Barasa MS, King’ola N, Mandaliya K, et al. Impact of five years of peer-mediated interventions on sexual behavior and sexually transmitted infections among female sex workers in Mombasa, Kenya. BMC Public Health. 2008;8:143. 18445258 10.1186/1471-2458-8-143 PMC2397398

[27] Luchters S, Richter ML, Bosire W, Nelson G, Kingola N, Zhang XD, et al. The contribution of emotional partners to sexual risk taking and violence among female sex workers in Mombasa, Kenya: a cohort study. PLoS One. 2013;8(8):e68855. 23950879 10.1371/journal.pone.0068855 PMC3737234

[28] Parcesepe AM, L’Engle KL, Martin SL, Green S, Suchindran C, Mwarogo P. Early sex work initiation and violence against female sex workers in Mombasa. Kenya J Urban Health. 2016;93(6):1010–26. 27714491 10.1007/s11524-016-0073-6 PMC5126017

[29] Tegang SP, Abdallah S, Emukule G, Luchters S, Kingola N, Baras M, et al. Concurrent sexual and substance-use risk behaviours among female sex workers in Kenya’s Coast Province: findings from a behavioural monitoring survey. SAHARA J. 2010;7(4):10–6. 21409306 10.1080/17290376.2010.9724972

[30] Were D, Musau A, Mutegi J, Ongwen P, Manguro G, Kamau M, et al. Using a HIV prevention cascade for identifying missed opportunities in PrEP delivery in Kenya: results from a programmatic surveillance study. J Int AIDS Soc. 2020;23(Suppl 3):e25537. 32602658 10.1002/jia2.25537 PMC7325512

[31] Bhattacharjee P, McClarty L, Isac S, Kimani J, Emmanuel F, Kabuti R, Kinyua A, Kombo BK, Owek C, Musyoki H, Kiplagat A, Arimi P, Shaw SY, Gandhi M, Malone S, Blanchard J, Garnett G, Becker ML. Applying the Effective Programme Coverage framework to assess gaps in HIV prevention programmes for female sex workers and men who have sex with men in Nairobi, Kenya: findings from an expanded Polling Booth Survey. J Int AIDS Soc. 2024;27 Suppl 2(Suppl 2):e26240. 10.1002/jia2.26240 PMC11233849 38982888

[32] Central Bureau of Statistics (CBS) [Kenya] MoHMK, and ORC Macro. Kenya demographic and health survey 2003. Calverton: CBS, MOH, and ORC Macro; 2004.

[33] Kenya National Bureau of Statistics (KNBS) and ICF Macro. Kenya Demographic and health survey 2008–09. Calverton: KNBS and ICF Macro; 2010.

[34] Vandenhoudt HM, Langat L, Menten J, Odongo F, Oswago S, Luttah G, et al. Prevalence of HIV and other sexually transmitted infections among female sex workers in Kisumu, Western Kenya, 1997 and 2008. PLoS One. 2013;8(1): e54953. 23372801 10.1371/journal.pone.0054953 PMC3553007

[35] Lafort Y, Greener R, Roy A, Greener L, Ombidi W, Lessitala F, et al. HIV prevention and care-seeking behaviour among female sex workers in four cities in India, Kenya, Mozambique and South Africa. Trop Med Int Health. 2016;21(10):1293–303. 27479236 10.1111/tmi.12761

[36] National AIDS and STI Control Programme (NASCOP). Kenya HIV estimates 2015. 2016.

[37] Luchters SM, Vanden Broeck D, Chersich MF, Nel A, Delva W, Mandaliya K, et al. Association of HIV infection with distribution and viral load of HPV types in Kenya: a survey with 820 female sex workers. BMC Infect Dis. 2010;10:18. 20102630 10.1186/1471-2334-10-18 PMC2845133

[38] Chersich MF, Luchters SM, Malonza IM, Mwarogo P, King’ola N, Temmerman M. Heavy episodic drinking among Kenyan female sex workers is associated with unsafe sex, sexual violence and sexually transmitted infections. Int J STD AIDS. 2007;18(11):764–9. 18005511 10.1258/095646207782212342

[39] Mishra S. Using mathematical models to characterize HIV epidemics for the design of HIV prevention strategies. Imperial College London; 2014. http://hdl.handle.net/10044/1/24913. Accessed 17 Oct 2023.

[40] Voeten HA, Egesah OB, Ondiege MY, Varkevisser CM, Habbema JD. Clients of female sex workers in Nyanza province, Kenya: a core group in STD/HIV transmission. Sex Transm Dis. 2002;29(8):444–52. 12172528 10.1097/00007435-200208000-00003

[41] R Core Team. R: a language and environment for statistical computing. Vienna, Austria: R Foundation for Statistical Computing; 2021.

[42] Boily MC, Lowndes CM, Vickerman P, Kumaranayake L, Blanchard J, Moses S, et al. Evaluating large-scale HIV prevention interventions: study design for an integrated mathematical modelling approach. Sex Transm Infect. 2007;83(7):582–9. 17942574 10.1136/sti.2007.027516 PMC2598645

[43] McKay MD, Beckman RJ, Conover WJ. A comparison of three methods for selecting values of input variables in the analysis of output from a computer code. Technometrics. 1979;21(2):239–45.

[44] Baeten JM, Richardson BA, Martin HL Jr, Nyange PM, Lavreys L, Ngugi EN, et al. Trends in HIV-1 incidence in a cohort of prostitutes in Kenya: implications for HIV-1 vaccine efficacy trials. J Acquir Immune Defic Syndr. 2000;24(5):458–64. 11035617 10.1097/00126334-200008150-00011

[45] Kavanaugh BE, Odem-Davis K, Jaoko W, Estambale B, Kiarie JN, Masese LN, et al. Prevalence and correlates of genital warts in Kenyan female sex workers. Sex Transm Dis. 2012;39(11):902–5. 23060082 10.1097/OLQ.0b013e318275ec7f PMC3506016

[46] van der Elst EM, Okuku HS, Nakamya P, Muhaari A, Davies A, McClelland RS, et al. Is audio computer-assisted self-interview (ACASI) useful in risk behaviour assessment of female and male sex workers, Mombasa, Kenya? PLoS One. 2009;4(5): e5340. 19412535 10.1371/journal.pone.0005340 PMC2671594

[47] Bengtson AM, L’Engle K, Mwarogo P, King’ola N. Levels of alcohol use and history of HIV testing among female sex workers in Mombasa. Kenya AIDS Care. 2014;26(12):1619–24. 25040114 10.1080/09540121.2014.938013 PMC4320941

[48] Manguro GO, Gichuki C, Ampt FH, Agius PA, Lim MS, Jaoko WG, et al. HIV infections among female sex workers in Mombasa, Kenya: current prevalence and trends over 25 years. Int J STD AIDS. 2020;31(14):1389–97. 33103582 10.1177/0956462420950571

[49] Parcesepe AM, L’Engle KL, Martin SL, Green S, Suchindran C, Mwarogo P. Early sex work initiation and condom use among alcohol-using female sex workers in Mombasa, Kenya: a cross-sectional analysis. Sex Transm Infect. 2016;92(8):593–8. 27217378 10.1136/sextrans-2016-052549 PMC5215884

[50] UNAIDS. UNAIDS data 2022. Geneva: UNAIDS; 2022.

[51] National AIDS and STI Control Programme (NASCOP). Kenya Population-based HIV Impact Assessment (KENPHIA) 2018: Final Report. Nairobi: NASCOP; 2022.

[52] Mishra S, Pickles M, Blanchard JF, Moses S, Boily MC. Distinguishing sources of HIV transmission from the distribution of newly acquired HIV infections: why is it important for HIV prevention planning? Sex Transm Infect. 2014;90(1):19–25. 24056777 10.1136/sextrans-2013-051250

[53] Mukandavire C, Walker J, Schwartz S, Boily MC, Danon L, Lyons C, et al. Estimating the contribution of key populations towards the spread of HIV in Dakar, Senegal. J Int AIDS Soc. 2018;21 Suppl 5(Suppl Suppl 5):e25126. 30033604 10.1002/jia2.25126 PMC6055131

[54] National AIDS and STI Control Programme (NASCOP). Key populations programme data collection tools. Revised reference manual - 2019. Nairobi: NASCOP; 2019.

[55] Richter M, Buthelezi K. Stigma, denial of health services, and other human rights violations faced by sex workers in Africa: “my eyes were full of tears throughout walking towards the clinic that i was referred to.” In: Goldenberg SM, Morgan Thomas R, Forbes A, Baral S, editors. Sex work, health, and human rights: global inequities, challenges, and opportunities for action. Cham: Springer International Publishing; 2021. p. 141–52. 36315792

[56] Decker MR, Lyons C, Guan K, Mosenge V, Fouda G, Levitt D, et al. A systematic review of gender-based violence prevention and response interventions for HIV key populations: female sex workers, men who have sex with men, and people who inject drugs. Trauma Violence Abuse. 2022;23(2):676–94. 35144502 10.1177/15248380211029405

[57] Decker MR, Wirtz AL, Pretorius C, Sherman SG, Sweat MD, Baral SD, et al. Estimating the impact of reducing violence against female sex workers on HIV epidemics in Kenya and Ukraine: a policy modeling exercise. Am J Reprod Immunol. 2013;69(Suppl 1):122–32. 23387931 10.1111/aji.12063

[58] Wirtz AL, Pretorius C, Beyrer C, Baral S, Decker MR, Sherman SG, et al. Epidemic impacts of a community empowerment intervention for HIV prevention among female sex workers in generalized and concentrated epidemics. PLoS One. 2014;9(2):e88047. 24516580 10.1371/journal.pone.0088047 PMC3916392

[59] Vassall A, Chandrashekar S, Pickles M, Beattie TS, Shetty G, Bhattacharjee P, et al. Community mobilisation and empowerment interventions as part of HIV prevention for female sex workers in Southern India: a cost-effectiveness analysis. PLoS One. 2014;9(10):e110562. 25333501 10.1371/journal.pone.0110562 PMC4204894

[60] Buckee C, Noor A, Sattenspiel L. Thinking clearly about social aspects of infectious disease transmission. Nature. 2021;595(7866):205–13. 34194045 10.1038/s41586-021-03694-x

[61] Decker MR, Pearson E, Illangasekare SL, Clark E, Sherman SG. Violence against women in sex work and HIV risk implications differ qualitatively by perpetrator. BMC Public Health. 2013;13(1):876. 24060235 10.1186/1471-2458-13-876 PMC3852292

[62] Lyons CE, Grosso A, Drame FM, Ketende S, Diouf D, Ba I, et al. Physical and sexual violence affecting female sex workers in Abidjan, Côte d’Ivoire: prevalence, and the relationship with the work environment, HIV, and access to health services. J Acquir Immune Defic Syndr. 2017;75(1):9–17. 28169873 10.1097/QAI.0000000000001310 PMC5870837

[63] Jewkes R, Milovanovic M, Otwombe K, Chirwa E, Hlongwane K, Hill N, et al. Intersections of sex work, mental ill-health, IPV and other violence experienced by female sex workers: findings from a cross-sectional community-centric national study in South Africa. Int J Environ Res Public Health. 2021;18(22):11971. 34831727 10.3390/ijerph182211971 PMC8620578

