# Supplementary material for: Exploratory analysis of the potential impact of violence on HIV among female sex workers in Mombasa, Kenya: a mathematical modelling study
Source: BMC Med. 2024 Oct 15;22:468. doi: 10.1186/s12916-024-03670-y (PMC11475892; doi:10.1186/s12916-024-03670-y)
Supplement: Supplementary file 1 — Additional file 1: Text S1-S6; Figures S1-S2; Tables S1-S4. Text S1 – Study setting. Text S2 – Description of underlying statistical analysis. Text S3 – Model details. Text S4 – Model equations. Text S5 – Model parameters. Text S6 – Model outputs, description of sensitivity analyses and additional details. Figure S1 – Model natural history of HIV and care cascade. Figure S2 – Model PrEP uptake over time. Table S1 – Model indices. Table S2 – Model state variables. Table S3 – Modelled changes in ART eligibility. Table S4– Model parameters. [file 12916_2024_3670_MOESM1_ESM.docx]

**Additional file 1: Description of study setting, statistical analysis used to generate model parameters, and model description**

1. Study setting:

Mombasa County, situated in the south-east of Kenya, has an estimated 8,187 female sex workers (FSWs), 4.2% of the national total of FSWs [1]. In Kenya, while sex work is criminalised [2], FSW programmes have been scaled up over the last decade, led by the National AIDS and STIs Control Programme (NASCOP) under the Kenyan Ministry of Health [3, 4]. Thus HIV testing rates and condom use with clients have both increased over time [4, 5]. Oral pre-exposure prophylaxis (PrEP) was introduced for FSWs in Kenya in 2016, although coverage remains low [6, 7].

1. Description of underlying statistical analysis for risk ratios and rates of experience of violence

In the present analysis we use parameters estimated from the Transitions Study dataset in [8]. Here we summarise the key points for the methods used in that analysis.

***Analysis to determine risk ratios for mediators:***

In the present analysis we use adjusted prevalence ratios (PRs) obtained from [8]. In that analysis, unadjusted and adjusted PRs with 95% confidence intervals were estimated using bivariate and multivariate Poisson regression models with robust error variance. Multivariate models for each violence exposure variable and each HIV prevention related outcome were adjusted for age (a priori), and were also adjusted for sociodemographic and sex work variables associated with the violence exposure variable in bivariate analysis (chi-square tests) at p<0.1. The variables adjusted for were: age and regular income (lifetime sexual violence and lifetime physical violence); age, ever married and duration in sex work (lifetime physical assault and arrest).

That analysis only considered the effects of lifetime violence, and did not consider different estimates for the effects of recent and non-recent violence on the mediators due to the insufficient sample size for that analysis.

The following potential mediators were considered:

- Condom non-use with clients
- HIV tested in the past year
- Ever contacted by peers or staff from HIV prevention programmes
- Registered with a HIV prevention programme
- Ever seen a condom demonstration
- Ever used a HIV prevention programme clinic or drop-in centre
- Ever STI tested
- STI tested in the past year

Of the above, the only associations were for condom non-use with SV and HIV testing with all three types of violence respectively.

***Estimating the prior parameter ranges for the rates that FSWs experience first time/recurrent violence:***

To estimate an approximate range for the rates that FSWs experience first-time and recurrent violence for a given type of violence, a simplified mathematical model (of violence alone) with only three states (never experienced that type of violence, recent experience, non-recent experience) and examined younger FSWs only (so ignoring HIV entirely, and assuming that the population size is constant). This simplified model was then solved at equilibrium for each type of violence separately (in other words assuming that the prevalence of both recent and non-recent violence is not changing over time), using the Transitions data prevalences of recent and non-recent violence (and the values of other parameters listed in Table S4). These give the rates in Main Text Table 1.

Since this analysis relies upon assuming no HIV transmission, we calibrate the full model to the prevalence of violence in younger FSWs (Main Text Figure 3B) and cross-validate prevalence of recent SV and PAA in older FSWs (Main Text Figure 3C) to ensure that the parameter ranges do reproduce the estimates of prevalence of recent and ever experienced violence. Additional File 2: Figure S7 shows that the estimated prevalence of each type of violence in the full model with HIV transmission remains fairly stable, suggesting that the prevalence of violence is not substantially modified by the HIV epidemic.

1. Model details

***Notation used***

Throughout, Tables S1 and S2 show the indices we use for parameters and state variables respectively throughout this supplementary text. We also show the definitions of parameters used in the differential equations, as well as the definitions of all other symbols used in this supplementary text.

**Table S1:** Table of indices used.

| **Index** | **Definition** | **Values** |
| --- | --- | --- |
| a | age group (FSWs only) | a=1 for younger FSWs  a=2 for older FSWs |
| j | stage of chronic HIV infection (acute infection and susceptible are different state variables) | j=1 CD4>350 cells/mm^3^  j=2 CD4 200-350 cells/mm^3^  j=3 CD4<200 cells/mm^3^ |
| k | HIV care cascade stage | k=1 ART-naïve  k=2 on ART  k=3 dropped out |
| p | experience of sexual violence | p=1 for no previous experience  p=2 for recent experience  p=3 for non-recent experience |
| q | experience of physical violence | q=1 for no previous experience  q=2 for recent experience  q=3 for non-recent experience |
| r | experience of police assault and arrest | r=1 for no previous experience  r=2 for recent experience  r=3 for non-recent experience |

We define the state variables as follows:

**Table S2:** Definitions of state variables used in the model.

| **State variable** | **Definition** |
| --- | --- |
| $X_{a,p,q,r}^{FSW}(t)$ | Susceptible FSWs |
| $A_{a,p,q,r}^{FSW}(t)$ | FSWs in acute HIV infection stage |
| $Y_{a,p,q,r,j,k}^{FSW}(t)$ | FSWs in chronic HIV infection stage |
| $X^{MCL}(t)$ | Susceptible clients |
| $A^{MCL}(t)$ | Clients in acute HIV infection stage |
| $Y_{j,k}^{MCL}(t)$ | Clients in chronic HIV infection stage |
| **Total population sizes:** | |
| $N_{a,p,q,r}^{FSW}(t)$ | Total number of FSW in age group a, sexual violence compartment p, physical violence compartment q, and police assault and arrest compartment r (i.e. summing over HIV disease and HIV care cascade stages only). These varies over time since the proportion of younger/older FSWs changes as described in section 3.4.1. |
| $N^{MCL}$ | Total number of clients. Note that this is kept constant by assumption. |

### ***Rate parameters used in the differential equations:***

The symbols corresponding to rate parameters used in the differential equations (Additional File 1: Text S4) are:

- $\gamma^{acute}$ – rate of HIV disease progression from acute to CD4>350 (j=1) stage;
- $\gamma_{j,k}$ – rate of chronic HIV disease progression from stage j to j+1 when in HIV care cascade stage k ($\gamma_{j,2}=0 \forall j$ since we assume no HIV progression when on ART; also $\gamma_{3,k}=0$ as no further HIV progression);
- $\alpha_{j}$ – rate of AIDS-related death in CD4 stage j when not on ART (k=1 or k=3);
- $\eta$ – relative rate of AIDS-related death when on ART (k=2) compared to not being on ART (k=1 or k=3);
- $\delta_{a,j,p,q,r}^{FSW}(t)$ – rate of initiation of FSWs onto ART from ART-naïve compartment, described in equations ( *3* )-( *6* );
- $\delta_{j}^{MCL}(t)$ – rate of initiation of clients onto ART from ART-naïve compartment, described in equation ( 7 );
- $\kappa^{FSW}$ – rate of FSWs stopping ART once started (not time-dependent);
- $\kappa^{MCL}$ – rate of clients stopping ART once started (not time-dependent);
- $\theta$ – rate of restarting ART from dropout (moving from k=3 to k=2 stage of HIV care cascade);
- $\mu_{a}^{FSW}$ – rate of FSWs aged $a$ stopping sex work (including death from non-HIV related causes);
- $\mu^{MCL}$ – rate of clients stopping buying sex (including death from non-HIV related causes);
- $\nu_{a}$ – rate of FSWs ageing from age group $a$ to $a+1$ (note that $\nu_{2}=0);$
- $\phi_{p}$ is the rate of progression from sexual violence compartment p to p+1. $\phi_{3}$ is zero;
- $\chi_{q}$ is the rate of progression from physical violence compartment q to q+1. $\chi_{3}$ is zero;
- $\psi_{r}$ is the rate of progression from police assault and arrest compartment r to r+1. $\psi_{3}$ is zero;
- $\tilde{\phi}_{3}$ is the rate of recurrence of sexual violence once experienced. $\tilde{\phi}_{1}$and $\tilde{\phi}_{2}$ are zero;
- $\tilde{\chi}_{3}$ is the rate of recurrence of physical violence once experienced. $\tilde{\chi}_{1}$and $\tilde{\chi}_{2}$ are zero;
- $\tilde{\psi}_{3}$ is the rate of recurrence of police assault and arrest once experienced. $\psi_{1}$and $\tilde{\psi}_{2}$ are zero;
- $B_{a=1,p=1,q=1,r=1}^{FSW}(t)$ and $B^{MCL}(t)$are the rates at which new FSWs (assumed to be in the younger age group, HIV-negative and with no prior experience of any form of violence) and clients (assumed HIV-negative) enter the population defined in equations (1-2);
- $\lambda_{a,p,q,r}^{FSW}(t)$ and $\lambda^{MCL}(t)$ are the force of infection terms defined in equations (11-12).

### ***Other symbols:***

- t denotes time (in years);
- $\omega_{j}(t)$ – the ART eligibility parameter at time t (representing how ART eligibility guidelines change over time);
- $\varepsilon_{a,p,q,r}^{FSW}(t)$ – the per-capita HIV testing rate for FSWs at time t (depends on age and experience of violence);
- ${RR}_{p,q,r}^{test}$ – the risk ratio for HIV testing due to being in sexual violence compartment p, physical violence compartment q and police assault and arrest compartment r, compared to a FSW who has never experienced any form of violence;
- $\pi_{a}^{FSW}$ – the age-dependent fraction of treatment-eligible FSWs starting ART following diagnosis;
- $\zeta^{MCL}(t)$ – rate of ART initiation among eligible clients at time t;
- $c_{a}^{FSW}$ is the number of clients seen by FSW per unit time;
- $\hat{c}^{MCL}(t)$ is the number of FSWs seen by a client per unit time at time t (this is a time-varying quantity, to ensure balancing of sexual partnerships as described below);
- $c_{init}^{MCL}$ is the number of FSWs seen by a client per unit time at the start of the simulation $t=t_{0}$ (so that $\hat{c}^{MCL}(t_{0}=c_{init}^{MCL})$);
- $n$ – the number of sexual acts per partnership;
- $f_{a}^{PrEP}\left( t \right)$ – the fraction of sex acts per partnership for which PrEP is used by FSWs in age group a at time t;
- $f_{a}^{PrEP, now}$– the fraction of sex acts per partnership for which PrEP is used by FSWs in age group a from 2018 onwards;
- $f_{a,p}^{cond}\left( t \right)$ – the fraction of sex acts per partnership for which a condom is used by FSWs in age group a and sexual violence compartment p, at time t;
- $f_{a,no viol}^{no cond}\left( t \right)$ – the fraction of sex acts per partnership for which a condom is not used by FSWs in age group a who have never experienced violence, at time t;
- $f_{a}^{no cond, now}$ – the fraction of sex acts per partnership for which a condom is not used by FSWs in age group a who have never experienced violence from 2005 onwards;
- ${RR}_{SV}^{no cond}$ – the risk ratio for not using a condom if ever experienced sexual violence, compared to never having experienced sexual violence;
- eff^cond^, eff^PrEP^ and eff^ART^ are the effectivenesses of condoms, PrEP and ART respectively.

## *New entrants:*

As stated earlier the populations of FSWs and clients are both constant over time, through time-varying rates of new entrants to each population. New entrants are represented by the terms $B_{a,p,q,r}^{FSW}$ and $B^{MCL}$ for FSWs and clients respectively.

$B_{a,p,q,r}^{FSW}=0$ except when a=p=q=r=1 (in other words FSWs enter as younger FSWs with no prior experience of any form of violence) when it takes the following value:

|  | $B_{a,p,q,r}^{FSW}(t)=\sum_{a,p,q,r} \mu_{a}^{FSW}\left( X_{a,p,q,r}^{FSW}+A_{a,p,q,r}^{FSW}+\sum_{j,k} Y_{a,p,q,r,j,k}^{FSW} \right)+\sum_{a,p,q,r,j} \alpha_{j}\left( Y_{a,p,q,r,j,k=1}^{FSW}+Y_{a,p,q,r,j,k=3}^{FSW}+\eta Y_{a,p,q,r,j,k=2}^{FSW} \right)$ | ( 1 ) |
| --- | --- | --- |

$B^{MCL}$ is given by:

|  | $B^{MCL}(t)=\mu^{MCL}\left( X^{MCL}+A^{MCL}+\sum_{j,k} Y_{j,k}^{MCL} \right)+\sum_{j} \alpha_{j}\left( Y_{j,k=1}^{MCL}+Y_{j,k=3}^{MCL}+\eta Y_{j,k=2}^{MCL} \right)$ | ( 2 ) |
| --- | --- | --- |

## *Natural history of HIV, HIV care and antiretroviral therapy:*

**Figure S1: Model states and transitions related to natural history of HIV and HIV care cascade in the model.**


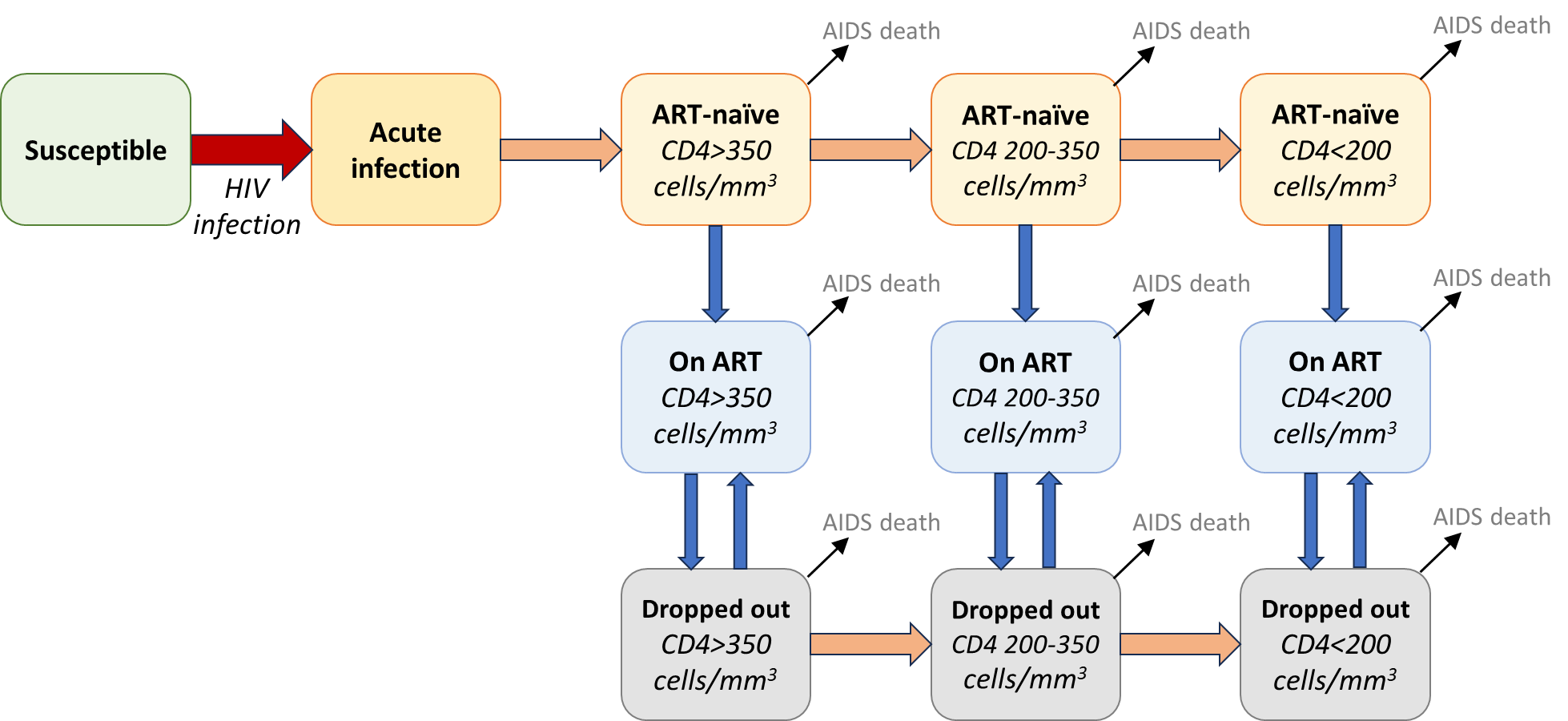


Figure S1 shows the natural history of HIV and HIV care cascade as represented in the model. Upon infection, individuals move from susceptible to acute infection, before moving to the CD4>350 cells/mm^3^ compartment. In the absence of antiretroviral therapy (ART) they progress sequentially to lower CD4 compartments with a small competing hazard of AIDS-related death in each chronic compartment.

Within the model there are three states related to HIV care: ART-naïve (never started antiretroviral therapy, ART), on ART, and dropped out from ART (Figure S1). Following HIV infection, FSWs are in the ART-naïve state. After initiating ART, individuals enter the “on ART” compartment. They then can drop out of ART (stopping ART), at rates $\kappa^{FSW}$ and $\kappa^{MCL}$ for FSW and clients respectively, entering the “dropped out” compartment. They can then restart ART at a given rate $\theta$ (given in Table S4). Individuals on ART no longer progress and have a reduced rate of AIDS-related death. Individuals who drop out from ART restart progression from the same CD4 compartment.

*ART eligibility:*

ART initiation only occurs in chronic infection, while ART eligibility guideline thresholds for initiation in Kenya have changed over time. Within the model these changes in guidelines are represented by the eligibility parameter $\omega_{j}(t)$, where $\omega_{j}(t)=0$ means that nobody in CD4 compartment j is eligible at time t, and $\omega_{j}(t)=1$ means that everybody in compartment j is eligible at time t. The <250 and <500 thresholds do not correspond to compartments in the model, so $\omega_{j}(t)$ is allowed to take values between 0 and 1 to account for this. Between 2007-2010, individuals in the model in the CD4 200-350 cells/mm^3^ compartment could start ART with $\omega_{2}(t)=1/3$, as approximately 1/3 of those in the 200-350 compartment would be eligible. Between 2014-16, $\omega_{1}(t)=1/2$, as approximately half of those in the CD4>350 compartment would meet the eligibility threshold [9].

Table S3 below shows the guideline changes and the corresponding values of $\omega_{j}(t)$.

**Table S3:** ART guideline thresholds for initiation in Kenya over time, from [10]. Final three columns show the corresponding values of the eligibility parameter $\omega_{j}(t)$.

| **Time period** | **ART eligibility criterion** | $\boldsymbol{\omega}_{\boldsymbol{1}}$  **(CD4>350 compartment)** | $\boldsymbol{\omega}_{\boldsymbol{2}}$  **(CD4 200-350)** | $\boldsymbol{\omega}_{\boldsymbol{3}}$  **(CD4<200)** |
| --- | --- | --- | --- | --- |
| Before 2003 | Not available | 0 | 0 | 0 |
| 2003-2007 | <200 cells/mm^3^ | 0 | 0 | 1 |
| 2007-2010 | <250 cells/mm^3^ | 0 | 1/3 | 1 |
| 2010-2014 | <350 cells/mm^3^ | 0 | 1 | 1 |
| 2014-2016 | <500 cells/mm^3^ | 1/2 | 1 | 1 |
| 2016 onwards | Any CD4 | 1 | 1 | 1 |

*HIV testing, ART initiation, and experiences of violence:*

For FSWs the per-capita ART initiation rate ($\delta_{a,j,p,q,r}^{FSW}(t)$) is the product of the per-capita HIV testing rate $\varepsilon_{a,p,q,r}^{FSW}(t)$, the age-dependent fraction of treatment-eligible FSWs starting ART following diagnosis ($\pi_{a}^{FSW}$), and the eligibility parameter $\omega_{j}(t)$.

|  | $\delta_{a,j,p,q,r}^{FSW}(t) = \varepsilon_{a,p,q,r}^{FSW}(t) \pi_{a}^{FSW}\omega_{j}(t)$ | ( 3 ) |
| --- | --- | --- |

The time-varying HIV testing rate for FSWs, $\varepsilon_{a,p,q,r}^{FSW}(t)$, depends on experience of violence, as described in the Main Text, through a decreased HIV testing rate among FSWs who have experienced sexual violence (index p>1), physical violence (q>1), or police assault and arrest (r>1). This in turn induces a dependency for the ART initiation rate on experience of violence. The rate of HIV testing for FSWs in the absence of violence (denoted $\varepsilon_{0}^{FSW}(t)$) is shown in main text Table 1. We denote ${RR}_{p,q,r}^{test}$ the risk ratio for HIV testing due to being in sexual violence compartment p, physical violence compartment q and police assault and arrest compartment r. Then the rate of HIV testing for FSWs at time t is:

|  | $\varepsilon_{a,p,q,r}^{FSW}\left( t \right)= {{RR}_{p,q,r}^{test} \varepsilon}_{0}^{FSW}\left( t \right)$ | ( 4 ) |
| --- | --- | --- |

where the value of ${RR}_{p,q,r}^{test}$ depends on the assumptions about how the effects of experiencing more than one form of violence affect HIV testing. In the main analysis, for FSWs experiencing more than one form of violence, we multiply their rate of HIV testing (and hence ART initiation) by the smallest multiplier:

|  | ${RR}_{p,q,r}^{test} = min({RR}_{p}^{test,SV},{RR}_{q}^{test,PV},{RR}_{r}^{test,PAA})$ | ( 5 ) |
| --- | --- | --- |

and for example ${RR}_{p}^{test,SV}$ takes the value 1 if the individual has never experienced sexual violence (p=1) and ${RR}^{test, SV}$ if they have (p>1):

|  | ${RR}_{p}^{test,SV}= \left\{ \begin{aligned} 1 \\ {RR}^{test, SV} \end{aligned} \right.\begin{matrix} if p=1 \\ if p>1 \end{matrix}$ | ( 6 ) |
| --- | --- | --- |

In the sensitivity analysis described in the main text and for which the results are shown in Additional File 2: Figure S19, we alternatively take the risk ratios to combine multiplicatively

$${RR}_{p,q,r}^{test,sensitivity analysis} = {RR}_{p}^{test,SV} \times{RR}_{q}^{test,PV}\times{RR}_{r}^{test,PAA}$$

Clients similarly can initiate ART at a time-dependent rate that follows ART eligibility guidelines $\delta_{j}^{MCL}(t)$. For simplicity we model this as simply a time-dependent rate of ART initiation among eligible clients $\zeta^{MCL}(t)$ multiplied by the eligibility parameter $\omega_{j}(t)$.

|  | $\delta_{j}^{MCL}(t) = \zeta^{MCL}(t) \omega_{j}(t)$ | ( 7 ) |
| --- | --- | --- |

As described in the Main Text, the model is fitted to ART coverage in 15-24 year old FSWs in 2015. In Additional File 2: Text S7 we show how the modelled ART coverage compares against additional data not used in parameterisation or fitting.

***Sexual partnerships***

HIV transmission occurs between FSWs and clients. As stated in the Main Text, sexual mixing for clients with younger/older FSWs is assumed to be proportionate to the number of partnerships offered by each group. However, we need to ensure balancing of sexual partnerships, in other words that the total numbers of partnerships of FSWs and of clients balance at all times.

*Balancing sexual partnerships:*

Although the total FSW population size is kept constant, the relative proportions of younger and older FSWs changes slightly in response to AIDS-related mortality. Since younger and older FSWs have slightly different numbers of clients seen per month, we adjust the number of FSWs that clients see per month dynamically during the simulation to ensure that FSW-client partner numbers balance.

**Balancing at the beginning of the simulation:** The number of clients seen by FSWs needs to equal the number of FSWs seen by clients at all times. At the start of the simulation we use the input parameter $c_{init}^{MCL}$ to set the total number of male clients $N^{MCL}$ (which then remains constant throughout the simulation).

**Balancing during the simulation:** Within the model the total number of FSW and clients are both kept constant over time through the time-varying entry rate of new FSWs and clients (see section 3.2 above). As stated in section 3.2, we assume that new FSWs enter the younger FSW compartment. However, since the number of FSWs exiting the model (which occurs either due to ceasing involvement with sex work, or AIDS-related mortality) varies over time as HIV prevalence and ART coverage changes, the number of new FSW entering the population also changes. This drives changes in the relative proportion of younger/older FSWs changes over time. Since younger and older FSWs see different numbers of clients per year, this means that there is a need to define what changes during the simulation to maintain the balance of sexual partnerships. We do this by allowing the number of FSW partners a client has per year, $\hat{c}^{MCL}(t)$, to be time-varying as follows:

|  | $\hat{c}^{MCL}(t) = \sum_{p,q,r,a} \frac{c_{a}^{FSW}N_{a,p,q,r}^{FSW}(t)}{N^{MCL}}$ | ( 8 ) |
| --- | --- | --- |

$N_{a,p,q,r}^{FSW}(t)$ is the total number of FSW (summed over susceptible and infected states, and HIV care states); as explained in the previous paragraph, this is time-varying. $N^{MCL}$ is the total number of clients (summed over susceptible and infected states, and HIV care states) as defined above. $c_{a}^{FSW}$ is the number of clients a FSW in age group *a* sees per year.

To check that this is not leading to large changes in the number of FSWs seen per month by clients in the simulation, for each calibrated run we calculate the maximum value divided by the minimum value across the simulation (i.e. across time). Across all calibrated runs, the largest change in the number of FSWs seen per month is 3.2% (and median is 1.5%). This is a small change, below 10% which is often used as a threshold in perturbation analysis, so we conclude that there is very little variation in the number of FSWs seen by clients per month within each run due to AIDS-related mortality.

***Condom use***

Condom use is modelled by assuming that, for each FSW compartment, a fraction of partnerships are always protected by condoms, and that in the remaining fraction of partnerships condoms are never used (the per-partnership transmission rates are described in section 3.7.2 below).

The fraction of partnerships protected by condoms is determined the following two steps:

**1. For FSWs who have never experienced sexual violence (*p*=0), calculate the fraction of partnerships for which a condom is not used for FSWs in in a given age group *a*,** $\boldsymbol{f}_{\boldsymbol{a,no viol}}^{\boldsymbol{no cond}}\left( \boldsymbol{t} \right)$:

$f_{a,no viol}^{no cond}\left( t \right)$ is a time-dependent, piecewise-linear function:

$$f_{a,no viol}^{no cond}\left( t \right) = \left\{ \begin{matrix} 1 if t<1990 \\ 1 - (1-f_{a}^{no cond, now})*\frac{(t-1990)}{(2005-1990)} if 1990\leq t<2005 \\ f_{a}^{no cond, now} if t\geq2005 \end{matrix} \right.$$

$f_{a}^{no cond, now}$ is the parameter “% of FSWs that have never experienced sexual violence who do not use condoms in partnerships (2005 onwards)” (by age group *a*) sampled from the range given in Main Text Table 1 (a=1 for younger FSWs, a=2 for older FSWs).

**2. Calculating the fraction of partnerships for which a condom is used by FSWs in a given compartment (depends on age group *a* and experience of sexual violence compartment *p*):**

|  | $f_{a,p}^{cond}\left( t \right)= \left\{ \begin{aligned} \left( 1-f_{a,no viol}^{no cond}\left( t \right) \right) \\ \left( 1-{RR}_{SV}^{no cond}f_{a,no viol}^{no cond}\left( t \right) \right) \end{aligned}\begin{matrix} if p=1 \\ if p>1 \end{matrix} \right.$ | ( 9 ) |
| --- | --- | --- |

where $f_{a,p}^{cond}\left( t \right)$ is the fraction of partnerships for which a condom is always used by FSWs in age-group compartment *a* at time *t* who are in the sexual violence experience compartment p (p=1 is no previous experience; p>1 is had previous experience, either recent or non-recent). ${RR}_{SV}^{no cond}$ is the risk ratio for not using a condom when having experienced sexual violence (compared to never experiencing), which acts here as a multiplier for increasing the fraction of sex acts in a partnership when condoms are not used amongst those who have experienced sexual violence. It is the parameter ${RR}_{SV}^{no cond}$ from Main Text Table 1. Note that we assume, as shown in the causal pathway diagram in the Main Text Figure 2, that sexual violence is the only modelled structural factor that affects condom use in this setting.

We show time trends from calibrated model runs, and how these compare to cross-validation data later in section 4.3.

***Pre-exposure prophylaxis (PrEP)***

*Data sources for PrEP:*

PrEP was first introduced into Kenya in 2016 [6]. The data underlying FSW PrEP coverage in the model comes primarily from polling booth survey carried out between May-September 2017 in Mombasa. In that survey 26% of older FSWs and 46% of younger FSWs reported being on PrEP [7]. However, recent comparisons between polling booth surveys and biomarker data suggests that there may be substantial overreporting in polling booth surveys when asking about PrEP, with a four-fold overreporting compared to estimates based on biomarkers in the same population [11]. Adjusting the self-reported polling booth estimates by this factor gives 12% among younger FSWs and 7% in older FSWs. While programmatic data can under- or over-estimate coverage [12], it can nonetheless provide a consistency check for this estimate. Were et al. [13] presented FSW programme data from counties including Mombasa on the number of FSWs attending clinics for PrEP. Combined with the FSW population size estimates for these counties from [1] as a denominator, this suggests that 6% of the estimated FSW population across those counties attended the month 1 follow-up for PrEP, roughly consistent with the adjusted polling booth estimate above (and much lower than the unadjusted polling booth survey estimate).

*Modelling PrEP:*

In the model, PrEP reduces the per-partnership probability of infection as described in section 3.7.2 below. We assume that the proportion of acts in a partnership for which a FSW in a given age group uses PrEP at any given time is a piecewise-linear curve that is zero until $t_{0}^{PrEP}=2016$ and plateaus in 2018, at a value $f_{a}^{PrEP, now}$ (12% for younger FSWs and 7% for older FSWs) as shown in equation ( *10*). These are also shown graphically in Figure S2 below.

|  | $f_{a}^{PrEP}\left( t \right) = \left\{ \begin{matrix} 0 if t<t_{0}^{PrEP} \\ f_{a}^{PrEP, now}\frac{(t-t_{0}^{PrEP})}{(2018-t_{0}^{PrEP})} if t_{0}^{PrEP}\leq t<2018 \\ f_{a}^{PrEP, now} if t\geq2018 \end{matrix} \right.$ | ( 10 ) |
| --- | --- | --- |

**PrEP and violence:** We assume no influence of any form of violence on PrEP use in the model, as there is currently limited and contradictory data on how violence affects the use of PrEP among FSWs (hence PrEP is not included in the causal pathway diagram in the Main Text Figure 2). Among clinic attendees in Nairobi, experience of physical violence was associated with higher PrEP use [14], hypothesised to be due to individuals who had experienced physical violence feeling at higher risk of HIV. A study in Uganda found no association between experiences of violence and either PrEP acceptability or PrEP initiation [15]. In Tanzania, a study found no evidence of association between rape (perpetrator not specified) and using PrEP in the previous 12 months [16]. Conversely, a qualitative study of FSWs in Tanzania and the Dominican Republic identified violence as a barrier to oral PrEP [17].

**Figure S2: Percentage of HIV-negative FSWs using oral pre-exposure prophylaxis (PrEP) by age group over time.** As PrEP uptake parameters are constant across all runs, there is no uncertainty in modelled uptake.


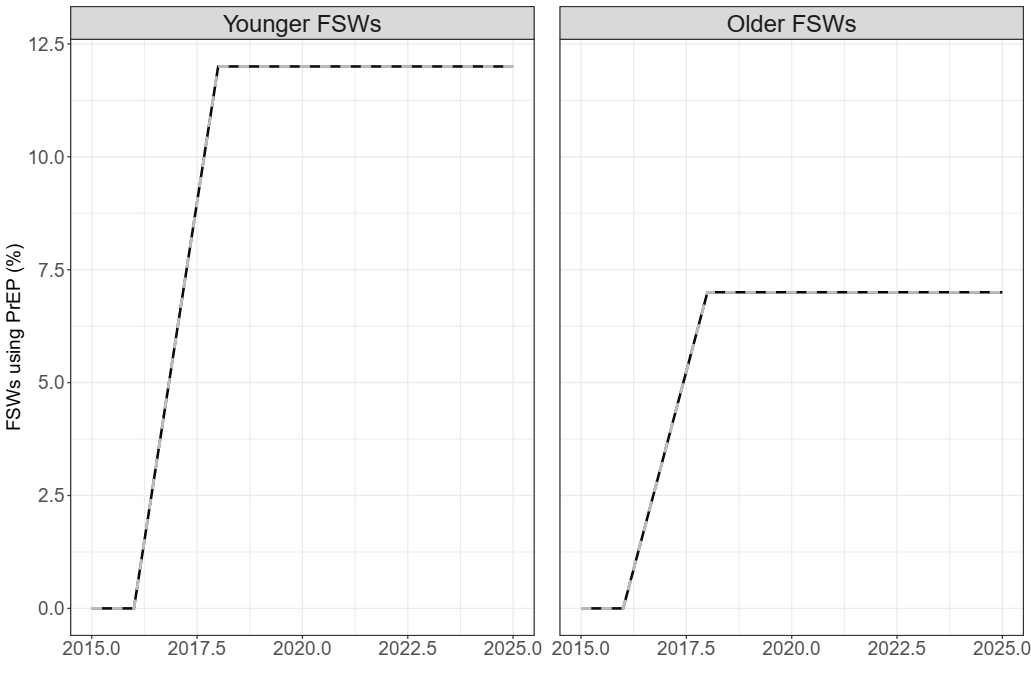


***HIV transmission***

We calculate per-partnership transmission probabilities based on per-act transmission probabilities.

*Per-act transmission probabilities:*

**Definitions:**

- $\beta_{0}$ is the baseline per-act transmission probability (the probability of F->M transmission per act when asymptomatic, i.e. CD4>200, with no condom, PrEP or ART use);
- $\rho^{M->F}$ is the relative risk of male-to-female transmission compared to that from female-to-male;
- $\rho^{acute}$ is the relative risk of HIV transmission in acute phase compared to CD4>200 chronic stages;
- $\rho_{j}^{chronic}$ is the relative risk of HIV transmission in chronic stage *j* compared to the CD4>200 chronic stages (so that $\rho_{1}^{chronic}=\rho_{2}^{chronic}=1$ by definition);
- ${eff}^{ART}$ is the effectiveness of ART.

***Per-act client-to-FSW transmission probabilities:***

For a client in the acute phase, the per-act transmission probability $p_{acute}^{FSW}$, is the product of the *baseline per-act transmission probability* $\beta_{0}$ multiplied by $\rho^{M->F}$ and$\rho^{acute}$ :

$$p_{acute}^{FSW}=\rho^{M->F}\rho^{acute}\beta_{0}$$

Similarly for chronic HIV infection, the per-act transmission probability from a client in stage j of chronic HIV infection and HIV care cascade stage k to a FSW is:

$$p_{j,k}^{FSW}=\left\{ \begin{aligned} \rho^{M->F}\rho_{j}^{chronic}\beta_{0} \\ (1-{eff}^{ART})\rho^{M->F}\rho_{j}^{chronic}\beta_{0} \end{aligned} \right. \begin{matrix} if k=1,3 \\ if k=2 \end{matrix}$$

***Per-act FSW-to-client transmission probabilities:***

We define the per-act transmission probability from a FSW in the acute phase to a client, $p_{acute}^{MCL}$, as the product of $\beta_{0}$ multiplied by $\rho^{acute}$:

$$p_{acute}^{MCL}=\rho^{acute}\beta_{0}$$

For chronic HIV infection, the per-act transmission probability from a client in stage j of chronic HIV infection and HIV care cascade stage k to a FSW, $p_{j,k}^{MCL}$, is given by:

$$p_{j,k}^{MCL}=\left\{ \begin{aligned} \rho_{j}^{chronic}\beta_{0} \\ (1-{eff}^{ART})\rho_{j}^{chronic}\beta_{0} \end{aligned} \right. \begin{matrix} if k=1,3 \\ if k=2 \end{matrix}$$

*Per-partnership transmission probability:*

The per-partnership transmission probability depends on the sex of the susceptible individual, the stage of HIV infection and HIV care cascade stage of the infected individual, and whether condoms and/or PrEP were used in the partnership. We calculate separate per-partnership transmission probabilities for each combination, using the relevant per-act transmission probability and the total number of sex acts per partnership (n).

**Client-to-FSW per-partnership HIV transmission:**

We calculate per-partnership transmission probabilities split into whether the client is in the acute or chronic stage of infection, and whether PrEP and/or condoms were used throughout the partnership.

*Acute client-to-FSW per-partnership transmission, no PrEP or condoms used in the partnership:*

$$\beta_{acute,a,p}^{FSW,noPrEP,nocond} = 1-\left( 1-p_{acute}^{FSW} \right)^{n}$$

*Acute client-to-FSW transmission, using PrEP but not condoms in the partnership:*

$$\beta_{acute,a,p}^{FSW,PrEP,nocond} = 1-\left( 1-\left( 1-{eff}^{PrEP} \right)p_{acute}^{FSW} \right)^{n}$$

*Acute client-to-FSW transmission, using condoms but not PrEP in the partnership:*

$$\beta_{acute,a,p}^{FSW,noPrEP,cond} = 1-\left( 1-\left( 1-{eff}^{cond} \right)p_{acute}^{FSW} \right)^{n}$$

*Acute client-to-FSW transmission, using both PrEP and condoms in the partnership:*

$$\beta_{acute,a,p}^{FSW,PrEP,cond} = 1-\left( 1-\left( 1-{eff}^{PrEP} \right)\left( 1-{eff}^{cond} \right)p_{acute}^{FSW} \right)^{n}$$

*Chronic client-to-FSW per-partnership transmission, no PrEP or condoms used in the partnership:*

$$\beta_{a,p,j,k}^{FSW,noPrEP,nocond} =1-\left( 1-p_{j,k}^{FSW} \right)^{n}$$

*Chronic client-to-FSW per-partnership transmission, using PrEP but not condoms in the partnership:*

$$\beta_{a,p,j,k}^{FSW,PrEP,nocond} =1-\left( 1-\left( 1-{eff}^{PrEP} \right)p_{j,k}^{FSW} \right)^{n}$$

*Chronic client-to-FSW per-partnership transmission, using condoms but not PrEP in the partnership:*

$$\beta_{a,p,j,k}^{FSW,noPrEP,cond} =1-\left( 1-\left( 1-{eff}^{cond} \right)p_{j,k}^{FSW} \right)^{n}$$

*Chronic client-to-FSW per-partnership transmission, using both PrEP and condoms in the partnership:*

$$\beta_{a,p,j,k}^{FSW,noPrEP,nocond} =1-\left( 1-\left( 1-{eff}^{PrEP} \right)\left( 1-{eff}^{cond} \right)p_{j,k}^{FSW} \right)^{n}$$

**FSW-to-Client HIV transmission:**

For clients, as clients do not use PrEP, we split partnerships into partnerships in which condoms are not used, and where they are used, and whether the FSW is in the acute of chronic phase. Note that since different FSWs use condoms more or less, the fraction of partnerships when condoms are used is dependent on the age of the FSW (a) and their sexual violence compartment (p) – this is therefore dealt with later when calculating the force of infection.

*Acute FSW-to-client per-partnership transmission, no condoms used in the partnership:*

$$\beta_{acute}^{MCL,nocond} = 1-\left( 1-p_{acute}^{MCL} \right)^{n}$$

*Acute FSW-to-client per-partnership transmission, using condoms in the partnership:*

$$\beta_{acute}^{MCL,cond} = 1-\left( 1-\left( 1-{eff}^{cond} \right)p_{acute}^{MCL} \right)^{n}$$

*Chronic FSW-to-client per-partnership transmission, no condoms used in the partnership:*

$$\beta_{j,k}^{MCL,nocond} = 1-\left( 1-p_{j,k}^{MCL} \right)^{n}$$

*Chronic client-to-FSW per-partnership transmission, using condoms in the partnership:*

$$\beta_{j,k}^{MCL,cond}=1-\left( 1-\left( 1-{eff}^{cond} \right)p_{j,k}^{MCL} \right)^{n}$$

*Force of infection:*

We first calculate the average transmission probabilities at time t (the expected per-partnership transmission probability averaged over condom and PrEP use in the specified age and sexual violence group and HIV infection and care cascade stages at time t):

$$\beta_{acute,a,p}^{FSW,average}(t)=\beta_{acute,a,p}^{FSW,noPrEP,nocond}(1-f_{a}^{PrEP}\left( t \right))(1-f_{a,p}^{cond}\left( t \right))+\beta_{acute,a,p}^{FSW,PrEP,nocond}f_{a}^{PrEP}\left( t \right)(1-f_{a,p}^{cond}\left( t \right))+\beta_{acute,a,p}^{FSW,noPrEP,cond}(1-f_{a}^{PrEP}\left( t \right))f_{a,p}^{cond}\left( t \right)+\beta_{acute,a,p}^{FSW,PrEP,cond}f_{a}^{PrEP}\left( t \right)f_{a,p}^{cond}\left( t \right)$$

$$\beta_{a,p,j,k}^{FSW,average}\left( t \right)=\beta_{a,p,j,k}^{FSW,noPrEP,nocond}\left( 1-f_{a}^{PrEP}\left( t \right) \right)\left( 1-f_{a,p}^{cond}\left( t \right) \right) +\beta_{a,p,j,k}^{FSW,PrEP,nocond}f_{a}^{PrEP}\left( t \right)(1-f_{a,p}^{cond}\left( t \right))+\beta_{a,p,j,k}^{FSW,noPrEP,cond}(1-f_{a}^{PrEP}\left( t \right))f_{a,p}^{cond}\left( t \right)+\beta_{a,p,j,k}^{FSW,PrEP,cond}f_{a}^{PrEP}\left( t \right)f_{a,p}^{cond}\left( t \right)$$

For clients, we calculate the per-partnership transmission probability with a FSW in age group a and sexual violence compartment p (we then sum over a and p in the force-of-infection equation):

$$\beta_{acute,a,p}^{MCL,average}(t)=\beta_{acute}^{MCL,nocond}(1-f_{a,p}^{cond}\left( t \right))+\beta_{acute}^{MCL,cond}f_{a,p}^{cond}\left( t \right)$$

$$\beta_{a,p,j,k}^{MCL,average}(t)=\beta_{j,k}^{MCL,nocond}(1-f_{a,p}^{cond}\left( t \right))+\beta_{j,k}^{MCL,cond}f_{a,p}^{cond}\left( t \right)$$

**Age-mixing:** Note that there is no age-mixing term for FSWs (since there is only one client age group). Age-mixing for clients is included directly in the equation below for $\lambda^{MCL}$.

The force of infection terms $\lambda_{a,p,q,r}^{FSW}$ and $\lambda^{MCL}$ are then given by the following equations:

|  | $\lambda_{a,p,q,r}^{FSW} = \frac{c_{a}^{FSW}\left( \beta_{acute,a,p}^{FSW,average}(t)A^{MCL}+\sum_{j,k} \beta_{a,p,j,k}^{FSW,average}(t)Y_{j,k}^{MCL} \right)}{N^{MCL}}$ | ( 11 ) |
| --- | --- | --- |

|  | $\lambda^{MCL}=\frac{\hat{c}^{MCL}(t)\sum_{a,p,q,r} c_{a}^{FSW}\left( \beta_{acute,a,p}^{MCL,average}(t)A_{a,p,q,r}^{FSW}+\sum_{j} \left( {\beta_{a,p,j,k}^{MCL,average}(t)Y}_{a,p,q,r,j,k}^{FSW} \right) \right)}{\sum_{a',p',q',r'} c_{a'}^{FSW}N_{a',p',q',r'}^{FSW}}$ | ( 12 ) |
| --- | --- | --- |

1. Model differential equations

**Susceptibles:**

$$\frac{dX_{a,p,q,r}^{FSW}}{dt} = B_{a,p,q,r}^{FSW}(t) -\left( \lambda_{a,p,q,r}^{FSW}(t)+\nu_{a}+\phi_{p}+\chi_{q}+\psi_{r}+\tilde{\phi}_{p}+\tilde{\chi}_{q}+\tilde{\psi}_{r}+\mu_{a}^{FSW} \right)X_{a,p,q,r}^{FSW}+\nu_{a-1}X_{a-1,p,q,r}^{FSW}+\phi_{p-1}X_{a,p-1,q,r}^{FSW} +\tilde{\phi}_{p+1}X_{a,p+1,q,r}^{FSW}+ \chi_{q-1}X_{a,p,q-1,r}^{FSW}+ \tilde{\chi}_{q+1}X_{a,p,q+1,r}^{FSW}+{\psi_{r-1}X}_{a,p,q,r-1}^{FSW}+\tilde{\psi}_{r+1}X_{a,p,q,r+1}^{FSW}$$

$$\frac{dX^{MCL}}{dt} = B^{MCL}(t) -\left( \lambda^{MCL}(t)+\mu^{MCL} \right)X^{MCL}$$

**Acute HIV infection:**

$$\frac{dA_{a,p,q,r}^{FSW}}{dt}=\lambda_{a,p,q,r}^{FSW}\left( t \right)X_{a,p,q,r}^{FSW}-\left( \gamma^{acute}+\nu_{a}+\phi_{p}+\chi_{q}+\psi_{r}+\tilde{\phi}_{p}+\tilde{\chi}_{q}+\tilde{\psi}_{r}+\mu_{a}^{FSW} \right)A_{a,p,q,r}^{FSW} \boldsymbol{+}\nu_{a-1}A_{a-1,p,q,r}^{FSW}+\phi_{p-1}A_{a,p-1,q,r}^{FSW} +\tilde{\phi}_{p+1}A_{a,p+1,q,r}^{FSW}+ \chi_{q-1}A_{a,p,q-1,r}^{FSW}+ \tilde{\chi}_{q+1}A_{a,p,q+1,r}^{FSW}+{\psi_{r-1}A}_{a,p,q,r-1}^{FSW}+\tilde{\psi}_{r+1}A_{a,p,q,r+1}^{FSW}$$

$$\frac{dA^{MCL}}{dt} = \lambda^{MCL}(t)X^{MCL} -\left( \gamma^{acute}+\mu^{MCL} \right)A^{MCL}$$

**Chronic HIV infection (by HIV care stage):**

*ART-naïve (k=1):*

$$\frac{dY_{a,p,q,r,j,k=1}^{FSW}}{dt}=\left[ j=1 \right]\gamma^{acute}A_{a,p,q,r}^{FSW}+\gamma_{j-1}Y_{a,p,q,r,j-1,k=1}^{FSW}-\left( \gamma_{j}+\delta_{a,j,p,q,r}^{FSW}(t)+\nu_{a}+\phi_{p}+\chi_{q}+\psi_{r}+\tilde{\phi}_{p}+\tilde{\chi}_{q}+\tilde{\psi}_{r}+\mu_{a}^{FSW}+ \alpha_{j} \right)Y_{a,p,q,r,j,k=1}^{FSW} \boldsymbol{+}\nu_{a-1}Y_{a-1,p,q,r,j,k=1}^{FSW}+\phi_{p-1}Y_{a,p-1,q,r,j,k=1}^{FSW} +\tilde{\phi}_{p+1}Y_{a,p+1,q,r,j,k=1}^{FSW}+ \chi_{q-1}Y_{a,p,q-1,r,j,k=1}^{FSW}+ \tilde{\chi}_{q+1}Y_{a,p,q+1,r,j,k=1}^{FSW}+{\psi_{r-1}Y}_{a,p,q,r-1,j,k=1}^{FSW}+\tilde{\psi}_{r+1}Y_{a,p,q,r+1,j,k=1}^{FSW}$$

$$\frac{dY_{j,k=1}^{MCL}}{dt}=\left[ j=1 \right]\gamma^{acute}A^{MCL}+\gamma_{j-1}Y_{j-1,k=1}^{MCL}-\left( \gamma_{j}+\delta_{j}^{MCL}(t)+\mu^{MCL}+ \alpha_{j} \right)Y_{j,k=1}^{MCL}$$

*On ART (k=2):*

$$\frac{dY_{a,p,q,r,j,k=2}^{FSW}}{dt}= \delta_{a,j,p,q,r}^{FSW}(t)Y_{a,p,q,r,j,k=1}^{FSW}+ \theta Y_{a,p,q,r,j,k=3}^{FSW}-\left( \kappa^{FSW}+\nu_{a}+\phi_{p}+\chi_{q}+\psi_{r}+\tilde{\phi}_{p}+\tilde{\chi}_{q}+\tilde{\psi}_{r}+\mu_{a}^{FSW}+ \eta\alpha_{j} \right)Y_{a,p,q,r,j,k=2}^{FSW} \boldsymbol{+}\nu_{a-1}Y_{a-1,p,q,r,j,k=2}^{FSW}+\phi_{p-1}Y_{a,p-1,q,r,j,k=2}^{FSW} +\tilde{\phi}_{p+1}Y_{a,p+1,q,r,j,k=2}^{FSW}+ \chi_{q-1}Y_{a,p,q-1,r,j,k=2}^{FSW}+ \tilde{\chi}_{q+1}Y_{a,p,q+1,r,j,k=2}^{FSW}+{\psi_{r-1}Y}_{a,p,q,r-1,j,k=2}^{FSW}+\tilde{\psi}_{r+1}Y_{a,p,q,r+1,j,k=2}^{FSW}$$

$$\frac{dY_{j,k=2}^{MCL}}{dt}=\delta_{j}^{MCL}(t)Y_{j,k=1}^{MCL}+ \theta Y_{j,k=3}^{MCL}-\left( \kappa^{MCL}+\mu^{MCL}+ \eta\alpha_{j} \right)Y_{j,k=2}^{MCL}$$

*Dropped out from ART (k=3):*

$$\frac{dY_{a,p,q,r,j,k=3}^{FSW}}{dt}=\gamma_{j-1}Y_{a,p,q,r,j-1,k=3}^{FSW}+ \kappa^{FSW}Y_{a,p,q,r,j,k=2}^{FSW}-\left( \gamma_{j}+\theta+\nu_{a}+\phi_{p}+\chi_{q}+\psi_{r}+\tilde{\phi}_{p}+\tilde{\chi}_{q}+\tilde{\psi}_{r}+\mu_{a}^{FSW}+ \alpha_{j} \right)Y_{a,p,q,r,j,k=3}^{FSW} \boldsymbol{+}\nu_{a-1}Y_{a-1,p,q,r,j,k=3}^{FSW}+\phi_{p-1}Y_{a,p-1,q,r,j,k=3}^{FSW} +\tilde{\phi}_{p+1}Y_{a,p+1,q,r,j,k=3}^{FSW}+ \chi_{q-1}Y_{a,p,q-1,r,j,k=3}^{FSW}+ \tilde{\chi}_{q+1}Y_{a,p,q+1,r,j,k=3}^{FSW}+{\psi_{r-1}Y}_{a,p,q,r-1,j,k=3}^{FSW}+\tilde{\psi}_{r+1}Y_{a,p,q,r+1,j,k=3}^{FSW}$$

$$\frac{dY_{j,k=3}^{MCL}}{dt}=\gamma_{j-1}Y_{j-1,k=3}^{MCL}+ \kappa^{MCL}Y_{j,k=2}^{MCL}-\left( \gamma_{j}+\theta+\mu^{MCL}+ \alpha_{j} \right)Y_{j,k=3}^{MCL}$$

For notational brevity, in the equations above we allow terms which have indices outside the ranges defined above (e.g. the term $\nu_{0}X_{0,p,q,r}^{FSW}$ in the first equation); however these terms should be treated as being equal to zero. [j=1] is the Iverson bracket that takes the value 1 if j=1, and 0 otherwise.

For clarity we explicitly show the time dependence for the time-dependent rate parameters. However, time-dependence is implicit for the state variables X, A, Y.

1. Model parameters

Table 1 in the Main Text shows the key model parameters, especially those related to the causal pathway by which violence affects HIV testing and condom use. Table S4 below shows the remaining model parameters.

**Table S4: Additional model parameters.**

| **Parameter** | **Value or prior range** | **Notes** |
| --- | --- | --- |
| **Biological parameters:** |  |  |
| Probability of HIV male-to-female  transmission per sex act in asymptomatic stage $\beta_{0}$ (CD4>200) | 0.0006-0.0011 | **From**  [18] |
| Risk ratio for per-act HIV transmission male-to-female, $\rho^{M->F}$, (compared to female-to-male) | 0.5-2.0 | **From**  [18] |
| Risk ratio for per-act HIV transmission (compared to CD4>200) | Acute ($\rho^{acute}$): 4.5-18.8  CD4 <200 ($\rho_{j=3}^{chronic}$): 4.5-11.9 | **From**  [18] |
| Duration in each HIV stage in the absence of ART (years) | Acute (${1/\gamma}^{acute}$): 0.1-0.5  CD4>350 (1/$\gamma_{1}$): 4.8-5.7  CD4 200-350 (1/$\gamma_{2}$): 4.7-6.5  CD4<200 (${1/\gamma}_{3}$): 1.4-2.8 | Acute: from [10].  CD4 200-350, >350 from [9].  CD4<200 from [19-21]. |
| HIV-related mortality rate per year when not on treatment when CD4 <200 | CD4>350 ($\alpha_{1}$): 0.010-0.012  CD4 200-350 ($\alpha_{2}$): 0.022-0.038  CD4<200: *value is* ${1/\gamma}_{3}$ *as defined above* | **From** [22]. Individuals die after the CD4<200 stage in the absence of treatment, so mortality rate with CD4<200 is the same as rate of leaving CD4<200 stage. |
| Relative rate of AIDS-related death when on ART compared to not being on ART, $\eta$ | 0.90 | **[20, 23-25]** |
| **Sexual behaviour:** |  |  |
| Rate of leaving sex work, $\mu_{a}^{FSW}$ | *Younger FSWs*: 0.065-0.092 yr^-1^  *Older FSWs:* 0.087-0.125 yr^-1^ | Younger FSWs from [8].  Older FSWs based on [5, 26-28] as analysed in [8]. |
| Rate of moving from younger to older FSW group, $\nu_{1}$ | 1/7.4 yr^-1^ | Based on the mean time between entering sex work and becoming 25 years old. Mean age at entry into sex work in Transitions was 17.6 years old [8]. |
| Rate at which clients stop paying for sex, $\mu^{MCL}$ | 1/14.0 yr^-1^ | From [29] |
| Number of clients seen by FSWs  per week ($=c_{a}^{FSW}/52)$ | *Younger FSWs*: 5.0-6.4  *Older FSWs:* 4.0-7.4 | From [8]  From [26] and [30] |
| Number of FSWs visited per month by clients at the start of the simulation (=$c_{init}^{MCL}$/12) (note that the number of FSWs visited per month changes during the simulation to ensure balancing of sexual partnerships as described in section 3.4.1) | 3.0-8.0 | From [29] |
| Number of sex acts per FSW-client partnership, n | 1-2 | From [26] |
| **HIV treatment and prevention:** |  |  |
| Eligibility criteria for initiating ART (CD4 cells/mm^3^) | Before 2007: <200  2007-2010: <250  2010-2014: <350  2014-2016: <500  2016 onwards: Any CD4 count | From [10] |
| Rate of ART uptake per year among ART-eligible clients | 2003: 0.03 yr^-1^  2006 onwards: 0.55-0.65 yr^-1^ | NASCOP 2018 Kenya HIV estimates report [10]. Note that ART uptake for FSWs is modelled differently (as the product of the HIV testing rate and the fraction of diagnosed FSWs starting ART) since violence affects HIV testing. |
| Rate of stopping ART among FSWs, $\kappa^{FSW}$ | 0.02-0.11 yr^-1^ | From [31] |
| Rate of stopping ART among clients, $\kappa^{MCL}$ | 0.09-0.16 yr^-1^ | From [32] |
| Rate of restarting ART if stopped/failed previously, $\theta$ | 0.1-0.5 yr^-1^ | Assumption that 10-50% of those who have dropped out restart ART per year. |
| Year when condom use is assumed to start | 1990 | As used in [33] |
| Year when condom use is assumed to plateau | 2005 | [5] found 29% of FSWs reported consistent condom use in 2000, but 70% reported in 2005. The latter is comparable to estimates from 2014-2017 [7, 34, 35], suggesting condom use is stable from 2005 onwards. |
| eff^PrEP^, Per-act PrEP efficacy against HIV transmission | 84% | [36] |
| eff^cond^, Per-act condom efficacy against HIV transmission | 78-95% | [37-39] |
| eff^ART^, Per-act effectiveness of ART against HIV transmission | 79-96% | Reviewed by [40], and consistent with estimates of ART adherence in [3]. |
| **Initial conditions:** |  |  |
| FSW population size in Mombasa in 2015 | 6016-10357 | From [1] |
| % of FSWs and clients infected with HIV in 1970 | 0.5-2% | Assumption |

1. Model outputs, sensitivity analyses and technical model details

##

***Estimating incidence of violence in the model***

For both younger and older FSWs we have additional state variables (not listed in the model equations) that record cumulative incident new/recurrent experiences of violence for each type of violence. Using this (and converting from cumulative episodes to episodes per year), the annual incident new/recurrent experiences of violence can be estimated. “New” incidence is estimated among FSWs who had not previously experienced that type of violence, while “recurrent” incidence is estimated among FSWs who had experienced that type of violence but not recently (i.e. not in the last six months).

Owing to the structure of the model we cannot estimate the number of episodes of violence among FSWs who have recently experienced that form of violence (since they must first leave the “recent violence” compartment), and hence the measure of recurrent incidence of violence is incidence among FSWs who have previously experienced that type of violence but not recently.

***Model sensitivity to the assumption about how risk ratios combine when FSWs experience multiple forms of violence***

As part of the sensitivity analysis, we examine the sensitivity of the results to the assumption described in the Causal Pathways Assumptions section of the Main Text, regarding how the risk ratios combine when FSWs experience multiple types of violence. Here, we modify the model so that risk ratios combine multiplicatively, but using the same posterior parameter set without refitting. We first compare the new outputs against the correlation data to see whether these have been substantially changed. We then compare the new estimates of incidence of violence in 2023, the 10-year impact of the intervention preventing future violence, and the effect of past violence experience from 2023 onwards, with those of the main analysis. The results are presented in Additional File 2: Figures S19 and S20.

***Model initialisation and software used***

The model is run for 50 years before HIV is introduced, to reach equilibrium levels of violence in the absence of HIV. HIV is introduced in 1970, with a randomly sampled fraction of FSWs and clients seeded in the CD4>350 compartment. The model is written in the C programming language and uses a fourth-order Runge-Kutta solver, with a timestep of 0.02 years. All model output analysis is carried out in R version 4.3.2 [41].

# References

1. National AIDS and STI Control Programme (NASCOP), *Key Population Size Estimates in Kenya, 2020: Final Report*, NASCOP, Editor. 2020: Nairobi, Kenya.

2. International Centre for Reproductive Health, *Report on the Female Sex Workers Program 2018 to 2021*, UNFPA, Editor. 2022.

3. Musyoki, H., et al., *A decade and beyond: learnings from HIV programming with underserved and marginalized key populations in Kenya.* J Int AIDS Soc, 2021. **24 Suppl 3**(Suppl 3): p. e25729.

4. Musyoki, H., et al., *Changes in HIV prevention programme outcomes among key populations in Kenya: Data from periodic surveys.* PLoS One, 2018. **13**(9): p. e0203784.

5. Luchters, S., et al., *Impact of five years of peer-mediated interventions on sexual behavior and sexually transmitted infections among female sex workers in Mombasa, Kenya.* BMC Public Health, 2008. **8**: p. 143.

6. Kamau, M., et al., *Unmet Need for Contraception Among Female Sex Workers Initiating Oral Pre-Exposure Prophylaxis for HIV Prevention During Kenya's National Scale-Up: Results From a Programmatic Surveillance Study.* Front Glob Womens Health, 2021. **2**: p. 747784.

7. National AIDS & STI Control Programme, M.o.H., *Third National Behavioural Assessment of Key Populations in Kenya: Polling Booth Survey Report.* 2018, NASCOP: Nairobi, Kenya.

8. Mountain, E., *HIV risk and prevention among sex workers: a focus on structural determinants and interventions*, in *School of Public Health*. 2017, Imperial College London.

9. Cori, A., et al., *CD4+ cell dynamics in untreated HIV-1 infection: overall rates, and effects of age, viral load, sex and calendar time.* AIDS, 2015. **29**(18): p. 2435-46.

10. National AIDS and STI Control Programme (NASCOP), *Kenya HIV Estimates 2018*. 2018.

11. Bhattacharjee, P., *Personal Communication*. 2023.

12. Stevens, O., et al., *Triangulation of Routine Antenatal HIV Prevalence Data and Adjusted HIV Estimates in Mozambique.* JAIDS Journal of Acquired Immune Deficiency Syndromes, 2024. **95**(1S): p. e70-e80.

13. Were, D., et al., *Using a HIV prevention cascade for identifying missed opportunities in PrEP delivery in Kenya: results from a programmatic surveillance study.* J Int AIDS Soc, 2020. **23 Suppl 3**(Suppl 3): p. e25537.

14. Leis, M., et al., *Intimate partner and client-perpetrated violence are associated with reduced HIV pre-exposure prophylaxis (PrEP) uptake, depression and generalized anxiety in a cross-sectional study of female sex workers from Nairobi, Kenya.* J Int AIDS Soc, 2021. **24 Suppl 2**(Suppl 2): p. e25711.

15. Witte, S.S., et al., *PrEP acceptability and initiation among women engaged in sex work in Uganda: Implications for HIV prevention.* EClinicalMedicine, 2022. **44**: p. 101278.

16. Faini, D., et al., *Awareness, Willingness and Use of HIV Pre-Exposure Prophylaxis Among Female Sex Workers Living in Dar-es-Salaam, Tanzania.* AIDS Behav, 2023. **27**(1): p. 335-343.

17. Mantsios, A., et al., *"She is the one who knows": A qualitative exploration of oral and injectable PrEP as part of a community empowerment approach to HIV prevention among female sex workers in the Dominican Republic and Tanzania.* PLOS Glob Public Health, 2022. **2**(9).

18. Boily, M.C., et al., *Heterosexual risk of HIV-1 infection per sexual act: systematic review and meta-analysis of observational studies.* Lancet Infect Dis, 2009. **9**(2): p. 118-29.

19. Morgan, D., et al., *Survival by AIDS defining condition in rural Uganda.* Sex Transm Infect, 2000. **76**(3): p. 193-7.

20. Kumarasamy, N., et al., *Natural history of human immunodeficiency virus disease in southern India.* Clin Infect Dis, 2003. **36**(1): p. 79-85.

21. Lodi, S., et al., *Time from human immunodeficiency virus seroconversion to reaching CD4+ cell count thresholds <200, <350, and <500 Cells/mm³: assessment of need following changes in treatment guidelines.* Clin Infect Dis, 2011. **53**(8): p. 817-25.

22. Lewden, C., et al., *CD4-specific mortality rates among HIV-infected adults with high CD4 counts and no antiretroviral treatment in West Africa.* J Acquir Immune Defic Syndr, 2012. **59**(2): p. 213-9.

23. Collaboration, H.-C., et al., *The effect of combined antiretroviral therapy on the overall mortality of HIV-infected individuals.* AIDS, 2010. **24**(1): p. 123-37.

24. Kitahata, M.M., et al., *Effect of early versus deferred antiretroviral therapy for HIV on survival.* N Engl J Med, 2009. **360**(18): p. 1815-26.

25. Ghate, M., et al., *Mortality in HIV infected individuals in Pune, India.* Indian J Med Res, 2011. **133**(4): p. 414-20.

26. Luchters, S., et al., *The contribution of emotional partners to sexual risk taking and violence among female sex workers in Mombasa, Kenya: a cohort study.* PLoS One, 2013. **8**(8): p. e68855.

27. Parcesepe, A.M., et al., *Early Sex Work Initiation and Violence against Female Sex Workers in Mombasa, Kenya.* J Urban Health, 2016. **93**(6): p. 1010-1026.

28. McClelland, R.S., et al., *A 15-year study of the impact of community antiretroviral therapy coverage on HIV incidence in Kenyan female sex workers.* AIDS, 2015. **29**(17): p. 2279-86.

29. Voeten, H.A., et al., *Clients of female sex workers in Nyanza province, Kenya: a core group in STD/HIV transmission.* Sex Transm Dis, 2002. **29**(8): p. 444-52.

30. Thomsen, S.C., et al., *A prospective study assessing the effects of introducing the female condom in a sex worker population in Mombasa, Kenya.* Sex Transm Infect, 2006. **82**(5): p. 397-402.

31. Geidelberg, L., et al., *Mathematical Model Impact Analysis of a Real-Life Pre-exposure Prophylaxis and Treatment-As-Prevention Study Among Female Sex Workers in Cotonou, Benin.* J Acquir Immune Defic Syndr, 2021. **86**(2): p. e28-e42.

32. NASCOP, *Annual Health Sector HIV Report 2009*. 2010.

33. Shannon, K., et al., *Global epidemiology of HIV among female sex workers: influence of structural determinants.* Lancet, 2015. **385**(9962): p. 55-71.

34. National STI/AIDS Control Programme, M.o.H., Kenya, *National Behavioral Assessment of Key Populations in Kenya Polling Booth Survey Report*. 2014, NASCOP: Nairobi, Kenya.

35. National AIDS & STI Control Programme, M.o.H., Kenya,, *Second National Behavioural Assessment of Key Populations in Kenya: Polling Booth Survey Report*. 2016, NASCOP: Nairobi, Kenya.

36. Stone, J., et al., *Impact and cost-effectiveness of the national scale-up of HIV pre-exposure prophylaxis among female sex workers in South Africa: a modelling analysis.* J Int AIDS Soc, 2023. **26**(2): p. e26063.

37. Weller, S. and K. Davis, *Condom effectiveness in reducing heterosexual HIV transmission.* Cochrane Database Syst Rev, 2002(1): p. CD003255.

38. Pinkerton, S.D. and P.R. Abramson, *Effectiveness of condoms in preventing HIV transmission.* Soc Sci Med, 1997. **44**(9): p. 1303-12.

39. Hughes, J.P., et al., *Determinants of per-coital-act HIV-1 infectivity among African HIV-1-serodiscordant couples.* J Infect Dis, 2012. **205**(3): p. 358-65.

40. Baggaley, R.F., et al., *Heterosexual HIV-1 infectiousness and antiretroviral use: systematic review of prospective studies of discordant couples.* Epidemiology, 2013. **24**(1): p. 110-21.

41. R Core Team, *R: A Language and Environment for Statistical Computing*. 2021, R Foundation for Statistical Computing: Vienna, Austria.
