# Supplementary material for: Exploratory analysis of the potential impact of violence on HIV among female sex workers in Mombasa, Kenya: a mathematical modelling study
Source: BMC Med. 2024 Oct 15;22:468. doi: 10.1186/s12916-024-03670-y (PMC11475892; doi:10.1186/s12916-024-03670-y)
Supplement: Supplementary file 2 — Additional file 2: Text S7-8; Figures S3-S20. Tables S5-S10. Text S7 – Model calibration and cross-validation. Text S8 – Additional results. Figures S3 and S4 – Model calibration and cross-validation to ART coverage. Figure S5 – Model cross-validation to condom use. Figure S6 – Model cross-validation to violence exposure. Figure S7 – Modelled prevalence of violence. Figure S8 – Modelled incidence of violence. Figure S9 – modelled percentage of ART-naïve people living with HIV. Figure S10 – comparison of ART coverage between scenarios. Figure S11- comparison of condom use between scenarios. Figure S12 and S13 – tPAF in FSWs and clients. Figure S14 – Prevalence of violence over time with/without intervention. Figure S15 and S16– impact of violence intervention for FSWs and clients. Figure S17 and S18 – scatter plots showing correlation between 10 year tPAF/10 year intervention impact and corresponding parameter values. Figures S19 and S20 – Sensitivity analysis results. Table S5 – HIV prevalence cross-validation data. Table S6 – ART coverage cross-validation data. Table S7 – condom use cross-validation data. Table S8 – Older FSW violence cross-validation data. [file 12916_2024_3670_MOESM2_ESM.docx]

**Additional file 2: Model fitting, cross-validation and additional model results**

1. Model fitting and cross-validation

In this section we first describe the data sources used in cross-validation. We then show how the model outputs compare to the calibration data, and then to the cross-validation data (some plots shown in Figure 3 in the Main Text).

***Model cross-validation data:***

Model cross-validation, through comparison of model estimates against empirical data not used in calibration, is an important step in modelling. When modelling structural factors, as described in [1], there is an additional need to cross-validate both the structural factors themselves (experiences of violence) and the proximate mediators (in this case ART coverage in FSWs and condom use). In this section, we present the data used in model cross-validation. We present the cross-validation plots against model outcomes in section 4.3 and in Main Text Figure 3.

The model calibration data is given in Main Text Table 1.

**Table S5: HIV prevalence cross-validation data.** Additional HIV prevalence data, by population group, used in cross-validating HIV prevalence model outputs.

| **Population group** | **Year** | **Value (95% CI)** | **Source/notes** |
| --- | --- | --- | --- |
| *FSW overall* | 1993 | 56.0 (53.4-58.5) | [2] |
|  | 1994 | 54.0 (46.8-61.1) | [2] |
|  | 1995 | 54.6 (50.5-58.7) | [2] |
|  | 1996 | 50.9 (47.4-54.5) | [2] |
|  | 1997 | 50.9 (46.6-55.2) | [2] |
|  | 2000 | 30.6 (26.6-34.9) | [3] |
|  | 2004 | 51.9 (50.0-54.7) | [4] |
|  | 2007 | 31.3 (24.5-38.7) | [5] |
|  | 2011.5 | 20.3 (17.6-23.2) | [6] |
|  | 2012.5 | 22.0 (17.9-26.4) | [7] |
|  | 2016 | 20.0 (16.2-23.8) | [8] |
| *Younger FSWs* | 2000 | 15.0 (4.2-33.7) | [3] |
|  | 2005 | 15.0 (4.3-34.9) | [3] |
|  | 2016 | 4.4 (2.5-6.3) | [8] |
| *Clients* | 2003 | 4.3 (1.2-11.5) | [9], for men reporting paying for sex in past 12 months |
|  | 2008.5 | 4.1 (1.0-12.7) | [10] , for men reporting paying for sex in past 12 months |

**Table S6: ART coverage cross-validation data.** Additional ART coverage data, by population group, used in cross-validating ART coverage model outputs. Due to the paucity of data on ART coverage, we additionally include data on ART coverage among the general population women and men, to provide information on the scale-up of ART over time in FSWs and clients respectively.

| **Population group** | **Year** | **Value (95% CI)** | **Source/notes** |
| --- | --- | --- | --- |
| FSW overall | 2012.5 | 39.3 (28.5-51.9) | [7] |
|  | 2017 | 73.0 | 2017 national polling booth survey [11]. |
| General population men [used as a proxy for trend in clients] | 2004 | 2.3 | Kenya HIV estimates report [12]. No CI was available, so only point estimate shown. |
|  | 2005 | 5.1 |  |
|  | 2006 | 11.5 |  |
|  | 2007 | 16.1 |  |
|  | 2008 | 21.9 |  |
|  | 2009 | 29.6 |  |
|  | 2010 | 36.2 |  |
|  | 2011 | 34.2 |  |
|  | 2012 | 37.5 |  |
|  | 2013 | 39.0 |  |
|  | 2014 | 42.7 |  |
|  | 2015 | 51.0 |  |
|  | 2016 | 55.9 |  |
|  | 2017 | 61.5 |  |
| General population men [used as a proxy for trend in clients] | 2018 | 62.0 (51.0-74.0) | UNAIDS [13] |
|  | 2019 | 59.0 (50.0-71.0) |  |
|  | 2020 | 65.0 (57.0-76.0) |  |
|  | 2021 | 77.0 (68.0-90.0) |  |
|  | 2022 | 74.0 (67.0-85.0) |  |
| General population men [used as a proxy for trend in clients] | 2019 | 68.6 (61.7-75.7) | KenPHIA [14] |
| General population women [used as a proxy for trend in FSW] | 2004 | 1.7 | Kenya HIV estimates report [12]. No CI was available. |
|  | 2005 | 3.9 |  |
|  | 2006 | 8.8 |  |
|  | 2007 | 12.3 |  |
|  | 2008 | 16.7 |  |
|  | 2009 | 22.6 |  |
|  | 2010 | 30.3 |  |
|  | 2011 | 42.8 |  |
|  | 2012 | 46.7 |  |
|  | 2013 | 50.2 |  |
|  | 2014 | 57.9 |  |
|  | 2015 | 67.7 |  |
|  | 2016 | 76.3 |  |
|  | 2017 | 82.5 |  |
| General population women [used as a proxy for trend in FSW] | 2018 | 83.0 (69.0-95.0) | UNAIDS [13] |
|  | 2019 | 75.0 (64.0-90.0) |  |
|  | 2020 | 80.0 (70.0-93.0) |  |
|  | 2021 | 91.0 (79.0-100.0) |  |
|  | 2022 | 82.0 (74.0-95.0) |  |
| General population women [used as a proxy for trend in FSW] | 2019 | 79.9 (76.0-83.9) | KenPHIA [14] |

**Table S7: Condom use cross-validation data.** List of condom use outcomes and values shown in cross-validation plot of condom use.

| **Outcome** | **Population group** | **Year** | **Value (95% CI)** | **Source** |
| --- | --- | --- | --- | --- |
| Consistent condom use with clients [no timescale given] | Older FSWs | 2000 | 28.8 (24.9-32.8) | Luchters 2008 [3] |
| Consistent condom use with clients [no timescale given] | Older FSWs | 2005 | 70.4 (66.4-74.3) | Luchters 2008 [3] |
| Consistent condom use with paying clients in the past 30 days | FSW overall (shown on both younger and older FSW plots) | 2007 | 65.0 (59.6-70.4) | Tegang 2010 [15] |
| Consistent condom use with paying partners [no timescale given] | Older FSWs | 2011 | 70.3 (67.1-73.4) | Parcasepe 2016 [16] |
| Always used a condom with paying clients in the past month | Older FSWs | 2014 | 62.0 (58.4-65.6) | 2014 Polling booth survey for Mombasa [17] |
| Always used a condom with paying clients in the past month | Older FSWs | 2015 | 72.0 (67.5-76.5) | 2015 Polling booth survey for Mombasa [18] |
| Consistent condom use with clients in the past week | Younger FSWs | 2015 | 77.5 (81.4-73.6) | Transitions 2015 [19] |
| Always used a condom with paying clients in the past month | Older FSWs | 2017 | 69.0 (66.0-72.0) | 2017 Polling booth survey for Mombasa [11] |
| Always used a condom with paying clients in the past month | Younger FSWs | 2017 | 69.0 (62.7-75.3) | 2017 Polling booth survey for Mombasa [11] |

**Table S8: Older FSW violence cross-validation data.** Additional data on the prevalence of recent experience (last 6 months) of police assault and arrest (PAA) and sexual violence (SV) in older FSWs, used in cross-validating violence model outputs.

| **Outcome** | **Year** | **% of older FSWs (95% CI)** | **Source** |
| --- | --- | --- | --- |
| Experienced PAA in the past six months | 2014 | 30.0 (26.6-33.4) | 2014 Polling booth survey for Mombasa [17] |
| Experienced PAA in the past six months | 2015 | 52.0 (47.0-57.0) | 2015 Polling booth survey for Mombasa [18] |
| Experienced PAA in the past six months | 2017 | 43.0 (39.8-46.2) | 2017 Polling booth survey for Mombasa [11] |
| Experienced SV in the past six months | 2014 | 17.0 (14.2-19.8) | 2014 Polling booth survey for Mombasa [17] |
| Experienced SV in the past six months | 2015 | 20.0 (16.0-24.0) | 2015 Polling booth survey for Mombasa [18] |
| Experienced SV in the past six months | 2017 | 17.0 (14.6-19.4) | 2017 Polling booth survey for Mombasa [11] |

***Plots of model calibration***

*Model calibration to ART coverage:*

**Figure S3: Model calibration to ART coverage**. Model time trends in ART coverage by population group (younger FSW, older FSW, overall FSW and clients. Points and error bars (black error bars show data used in calibration, blue error bars show cross-validation data, and brown error bars show data from the general population) show point estimate and 95% confidence interval of empirical data used in calibration. Solid black line shows median calibrated model estimate, and grey shaded region shows 95% credible interval of calibrated model estimates.


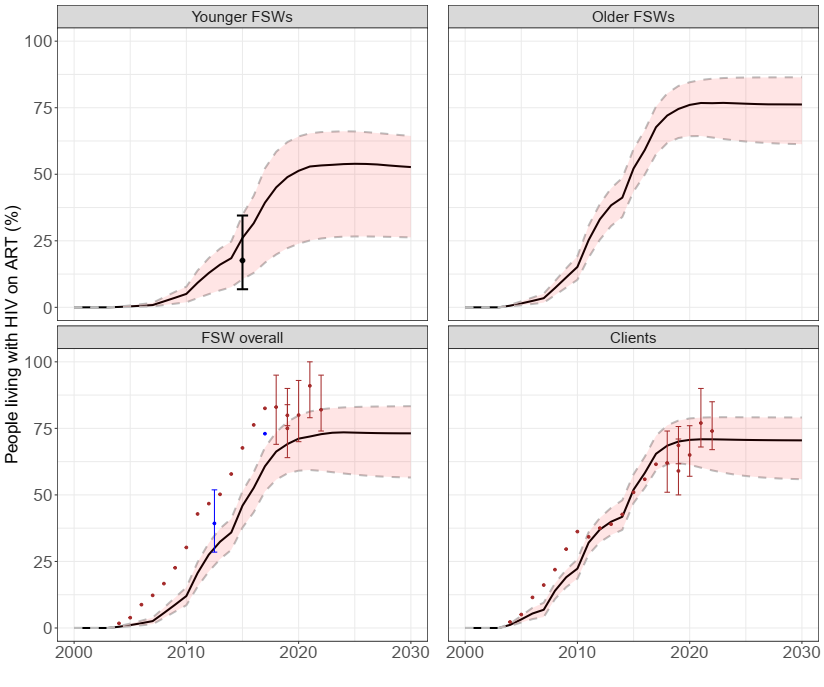


***Plots of model cross-validation***

*Model cross-validation to ART coverage:*

There is limited data on ART coverage in FSWs or clients, so we also present national estimates of ART coverage in general population women/men (the brown points/error bars in the “FSW overall” and “Clients” panels) consisting of national general population estimates 2004-2017 from [12], KenPHIA [14] and UNAIDS estimates 2017-2022 [13, 20-24] to provide a scale of magnitude for comparison, although it is likely that ART coverage in the general population will be higher than in FSWs [25].

**Figure S4: Model cross-validation to ART coverage.** Model time trends in ART coverage by population group among FSW overall (i.e. younger+older FSWs) and clients. Blue points and error bars show point estimate and 95% confidence intervals of available cross validation data specific to each risk group. Given the limited data on ART coverage in FSWs or clients, we also present national estimates of ART coverage in overall population of women and men (brown points/error bars on each panel) from [12], KenPHIA [14] for 2004-2017 and from UNAIDS for 2017-2022 [13, 20-24], to illustrate national scale up of ART over time in Kenya, which is likely higher than in FSWs [25]. Solid black line shows median calibrated model estimate, and grey shaded region shows 95% credibility intervals of calibrated model estimates. We do not show older FSWs as there is no cross-validation data for this group.


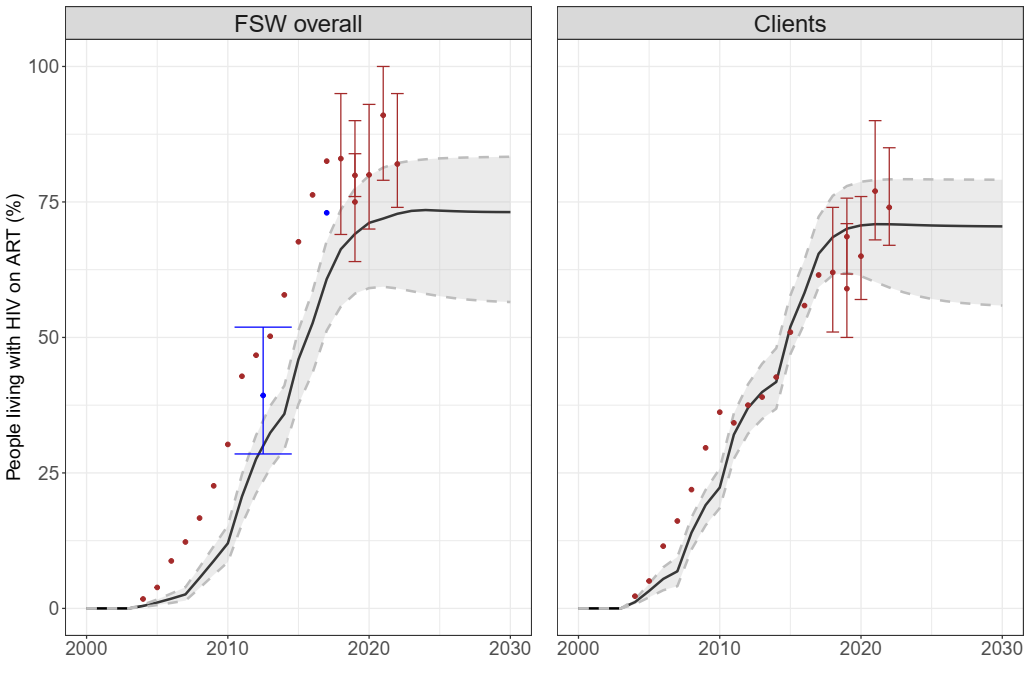


*Model cross-validation to condom use:*

As described in the Main Text, we show cross-validation to condom use, including against empirical data used to inform parameter choices. The reason for including this data is that the model input is a piecewise linear function of condom use in FSWs who have never experienced SV, so it is important to cross-validate the actual model output of condom use in FSWs (including experiences of SV) against the underlying data (listed in Table S7).

**Figure S5: Model cross-validation to condom use.** Overall proportion of FSWs using condoms consistently in calibrated model runs (grey shaded region shows 95% credible intervals across runs, solid black line shows median across runs). Blue points show survey data point estimates (error bars represent 95% confidence intervals) used to validate model outputs (the corresponding data sources are in Table S8).


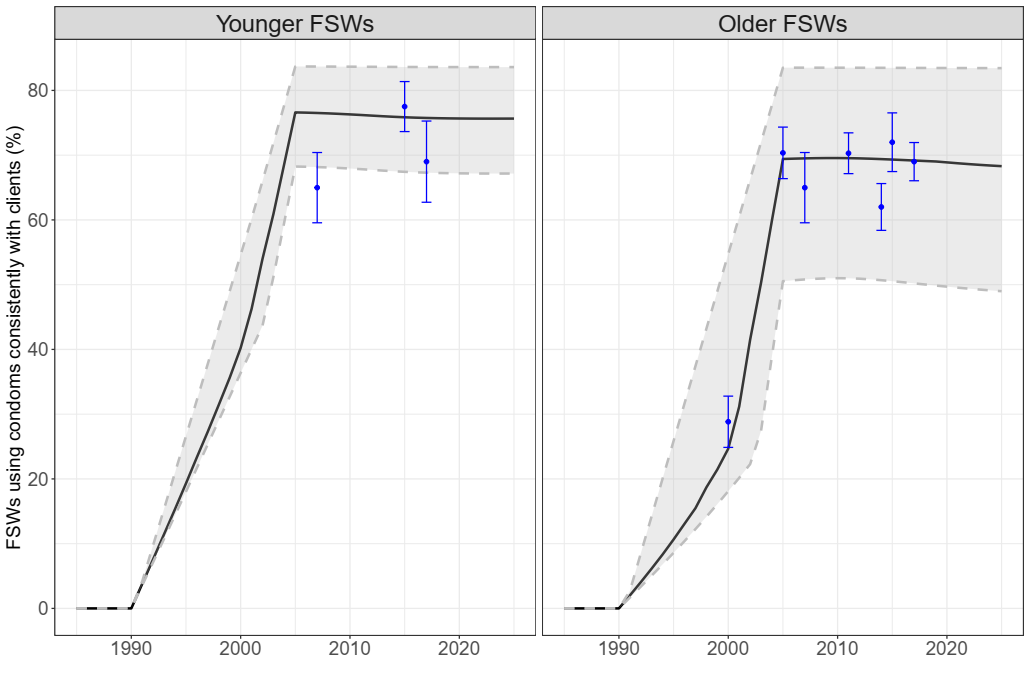


*Model cross-validation to experiencing multiple forms of violence:*

As described in the Main Text, in this model we do not assume that experience of one form of violence influences experience of others. To test this assumption of independence, we compare the model estimates of the percentage of FSWs who have experienced different combinations of violence against empirical data from the Transitions Study. These are shown below.

**Figure S6: Model cross-validation of experience of multiple forms of violence.** Cross-validation showing how the calibrated model run estimates of combinations of types of violence (red) compares to empirical data from the Transitions Study (blue). Error bars represent 95% credible intervals for calibrated model runs, and 95% confidence intervals for survey data. Recent violence=experienced that type of violence in the past six months. Ever violence=experienced either recent or non-recent violence of that type. SV=sexual violence; PV=physical violence; PAA=police assault or arrest.
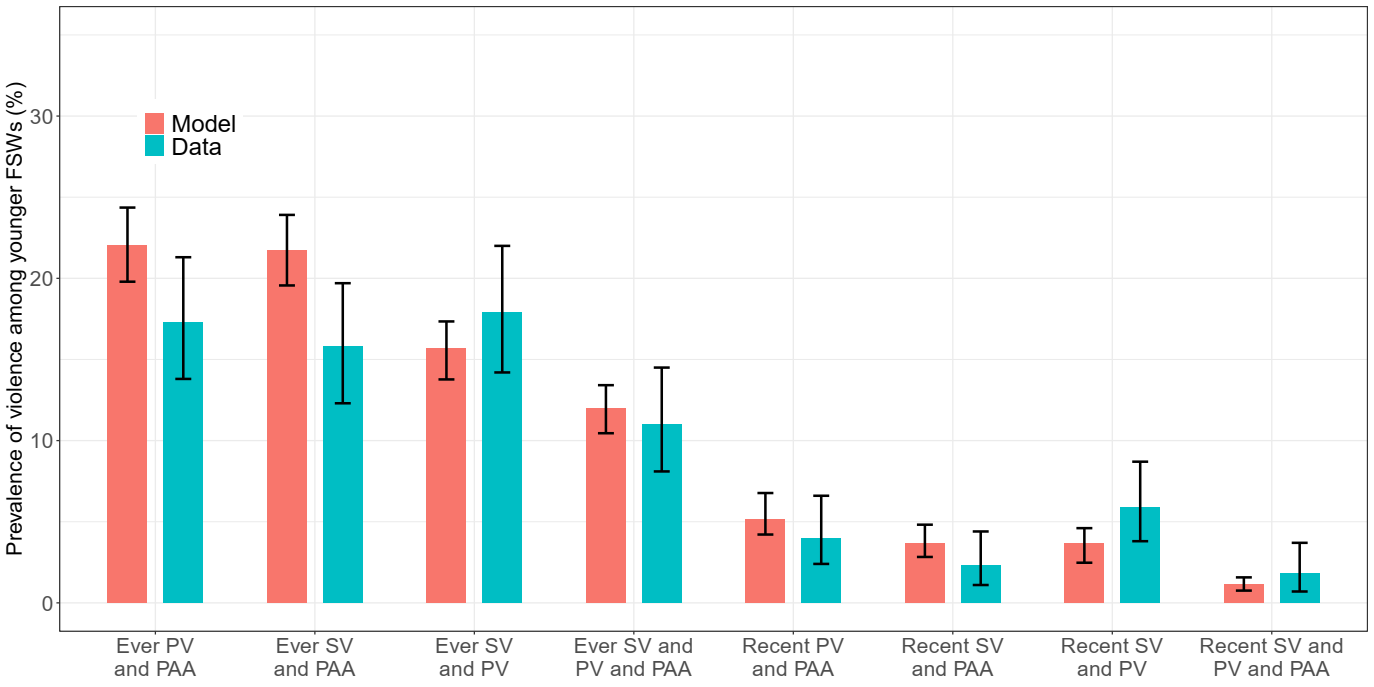


***Trends in violence experience over time***

Given the model, where the rate of violence experience is constant, it is unsurprising that there is little change over time in the percentage of FSWs (by age group) who have experienced recent violence or ever experienced violence. However, there is some impact from the HIV epidemic (and increased AIDS-related mortality) during the period 1980-2020 which causes a dip in this percentage, as FSWs who have experienced violence die at a higher rate from AIDS-related mortality.

**Figure S7: Model estimates of prevalence of violence.** Plot of calibrated model run prevalence estimates of recent and ever experienced violence (ever=recent+non-recent combined) over time among younger/older FSWs. Black line shows median and grey shaded region shows 95% credible intervals across calibrated runs. Points and error bars represent survey data mean/median and 95% confidence intervals; black points+error bars represent data used for calibration from [19], blue points+error bars represent validation survey data from [11, 17, 18]. SV=sexual violence; PV=physical violence; PAA=police assault or arrest.


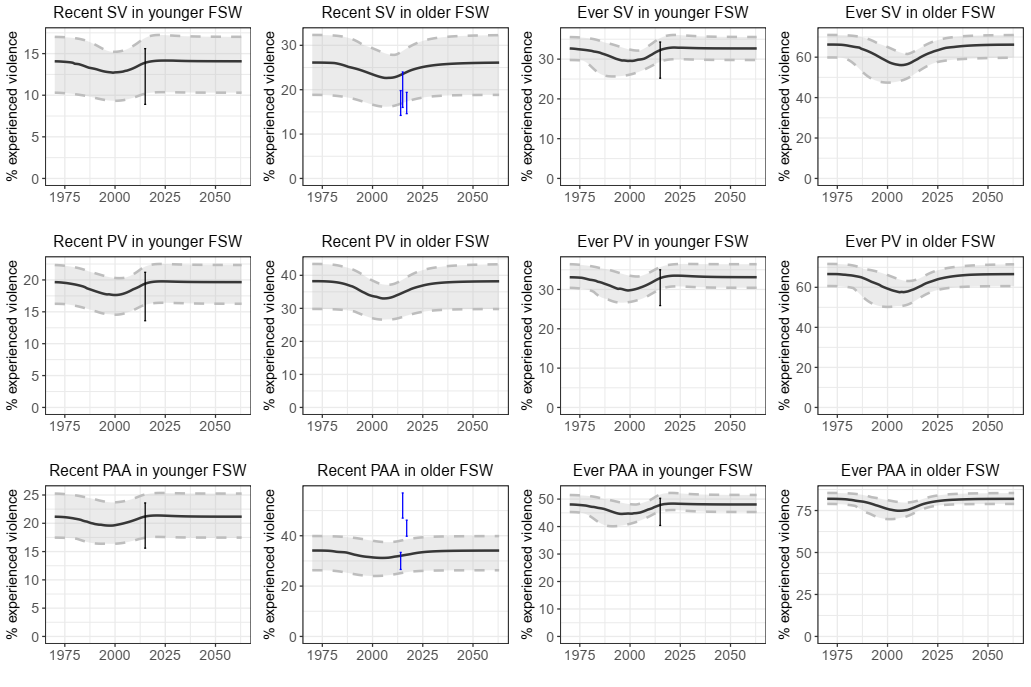


1. Additional results

***Model trends in incidence of violence***

**Figure S8: Model estimates of incidence of violence.** Trends in incidence of experiences of violence by whether first-time or recurrent (rows) and type of violence (columns). SV-sexual violence, PV=physical violence, PAA=police assault and arrest.


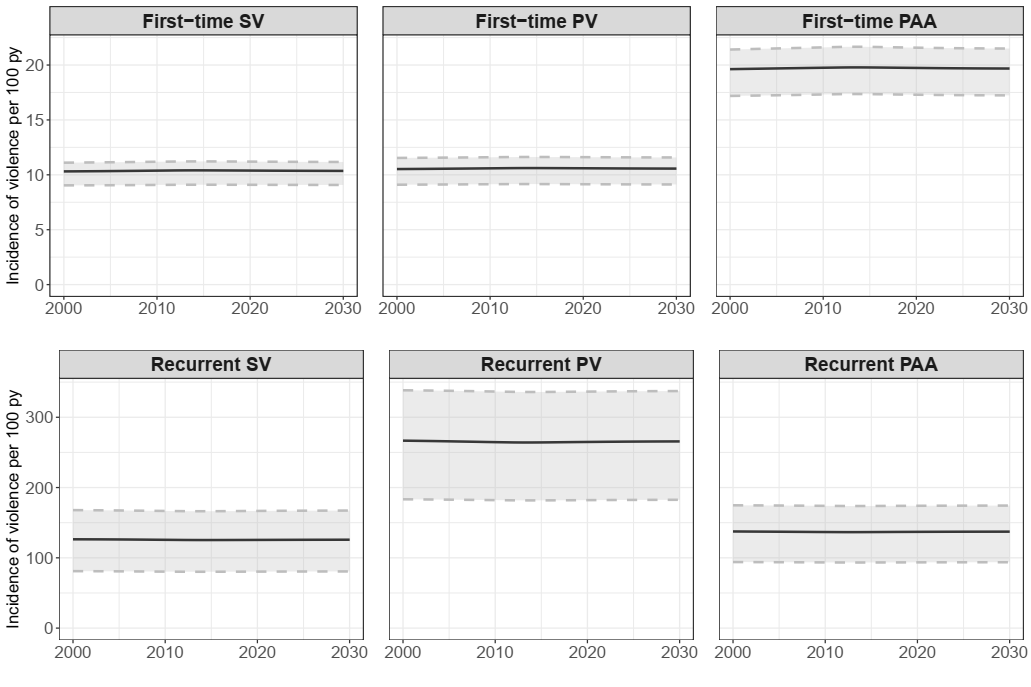


***Influence of violence on proximal mediators: ART uptake and condom use under “no effects of violence” tPAF analysis scenario compared to baseline scenario***

**Figure S9: Percentage of people living with HIV who are ART-naïve by population group.** Model time trends in ART coverage by population group (younger FSW, older FSW, overall FSW and clients. Solid black line shows median calibrated model estimate, and grey shaded region shows 95% credible interval of calibrated model estimates.


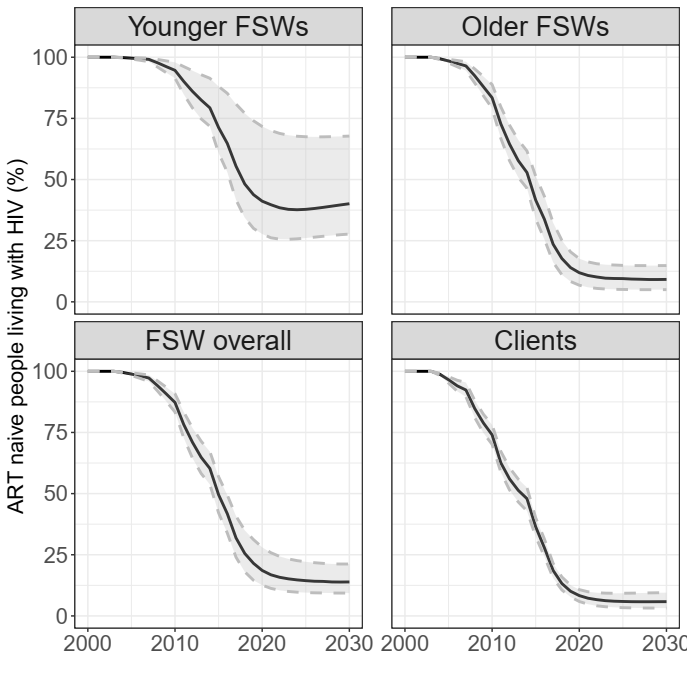


**Figure S10: Comparison of ART coverage for “no effects of violence” tPAF analysis scenario compared to baseline scenario.** ART coverage over time in all population groups when the effects of violence are switched off from 2023 onwards ("no effects from violence", shown in blue) against baseline plot (in red). Solid lines show median calibrated model value, and shaded region show 95% credible intervals. Since client testing/ART uptake behaviour is not influenced by violence, ART coverage is the same in both scenarios in this population group.


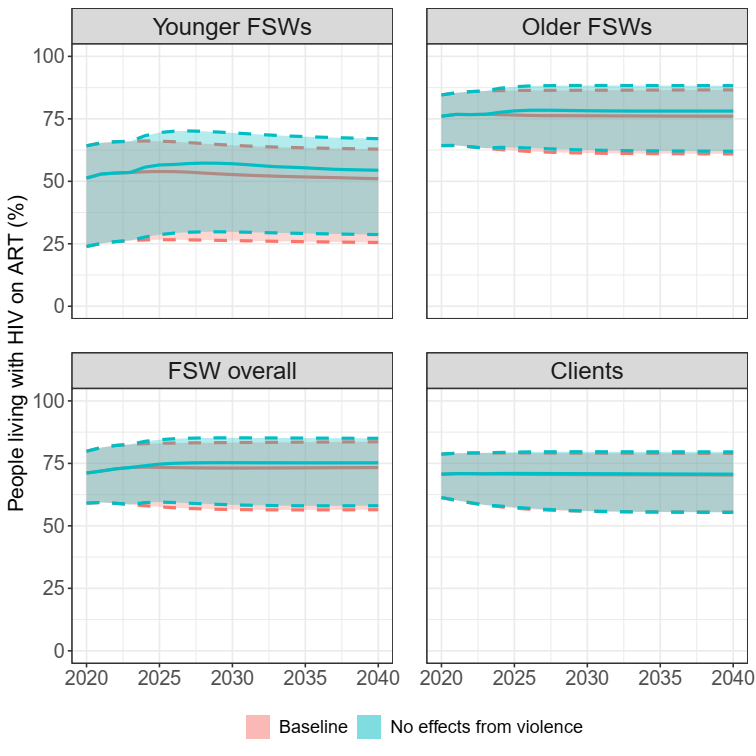


**Figure S11: Comparison of condom use trends over time with/without effects of violence.** Condom use trends over time in younger/older FSW when the effects of violence are switched off from 2023 onwards ("no effects from violence", shown in blue) against baseline plot (in red). Solid lines show median calibrated model value, and shaded region show 95% credible intervals.


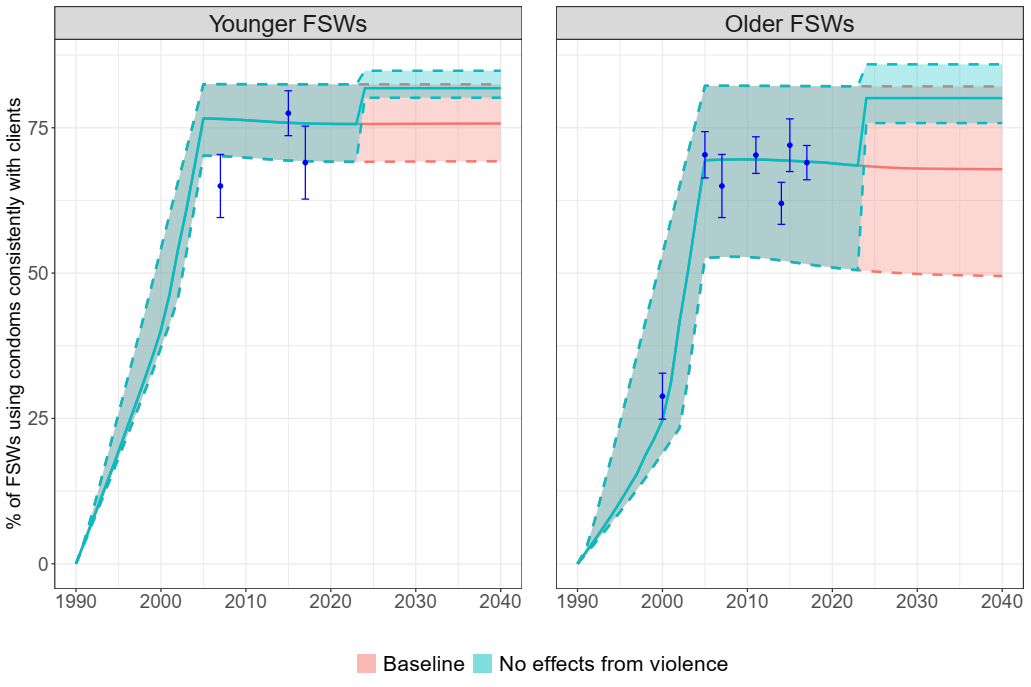


***tPAF in FSWs***

Here we show the tPAF results for FSWs only (the % of new infections in FSWs only).

**Figure 12: tPAF in FSWs.** Contribution of violence to HIV transmission: transmission population-attributable fractions (tPAFs) in FSWs over 10 and 40 years from 2023. Bars show median model estimates, and error bars show 95%CrIs. tPAF scenario labels are described in Main Text Table 2.


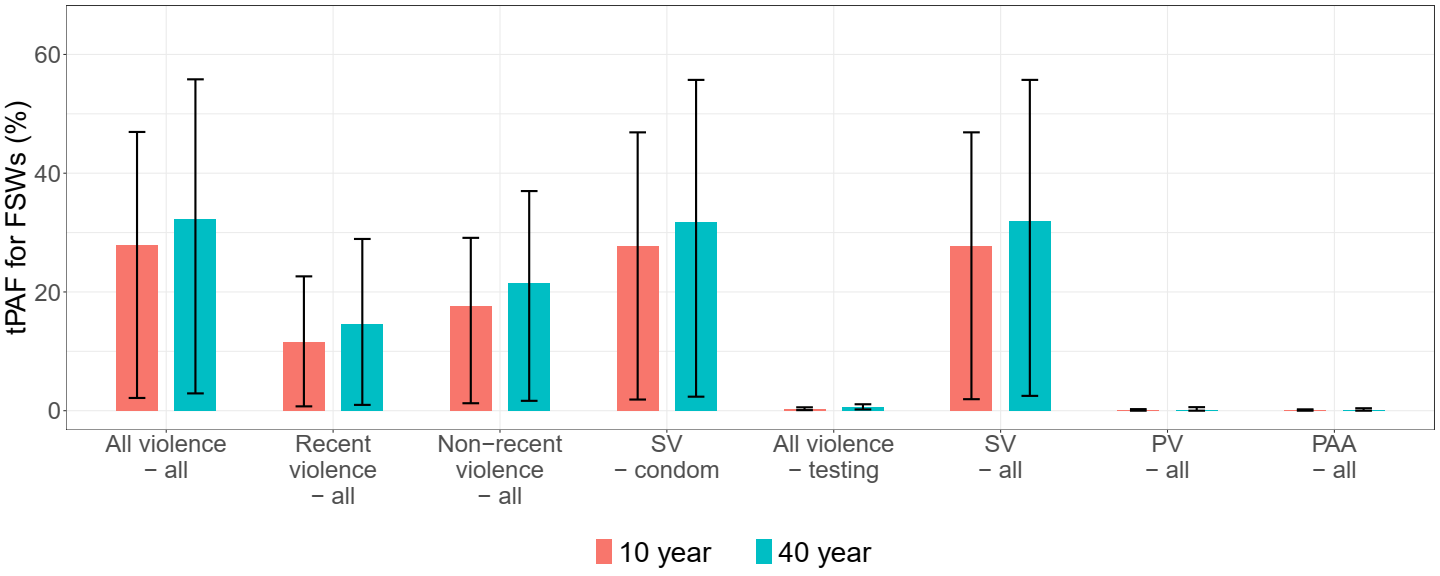


***tPAF in clients***

Here we show the tPAF results for clients only (the % of new infections in clients only).

**Figure S13: tPAF in clients.** Contribution of violence to HIV transmission: transmission population-attributable fractions (tPAFs) in clients over 10 and 40 years from 2023. Bars show median model estimates, and error bars show 95%CrIs. tPAF scenario labels are described in Main Text Table 2.


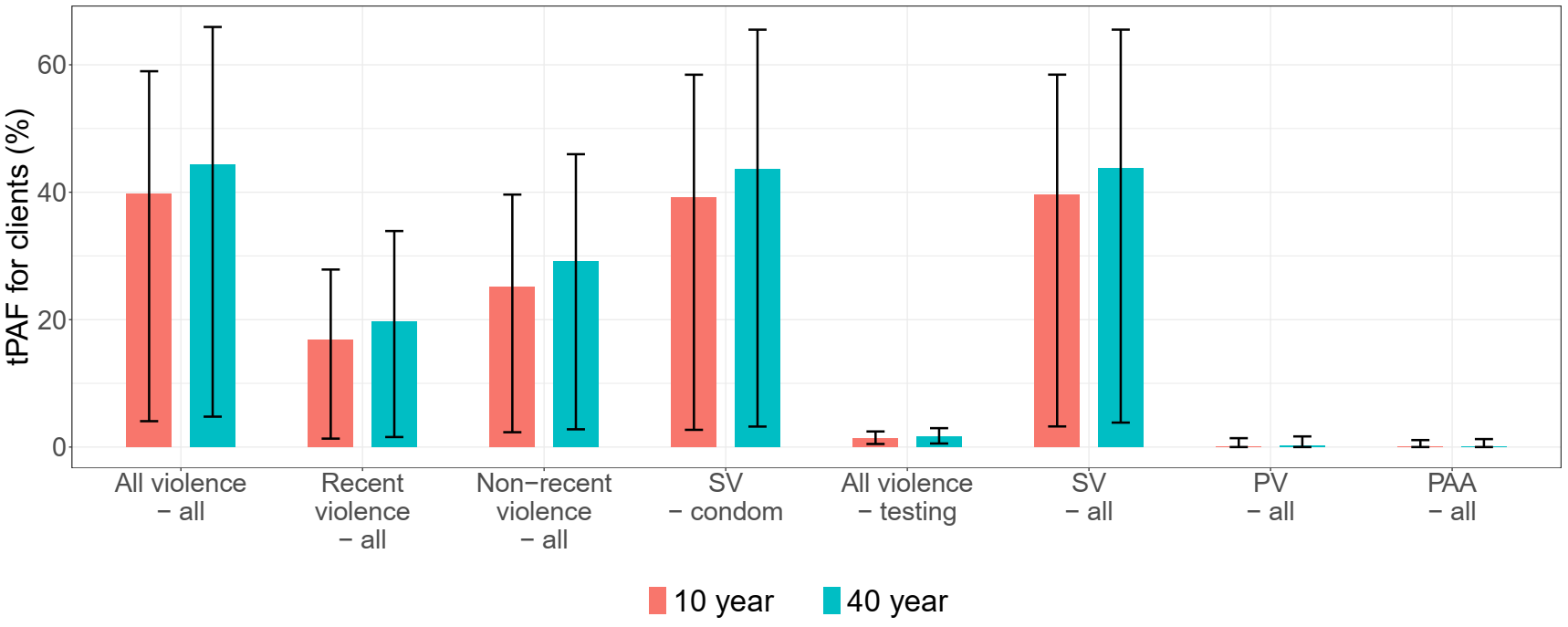


*Comparison of modelled trends in prevalence of recent/non-recent experienced violence for baseline against “prevent all future violence” scenario:*

**Figure S14: Prevalence of violence over time with/without violence prevention intervention.** Prevalence of female sex workers (FSWs) who have experienced violence recently (left two columns) and non-recently (right two columns) for each type of violence (rows). Red curve shows status quo; green lines show the scenario where all future violence is prevented (thus recent violence rapidly decreases to zero, although prevalence of ‘non-recent violence’ initially increases (due to ‘recent’ continuing to move to ‘non-recent’, without any movement from ‘non-recent’ to ‘recent’ owing to the prevention of further violence) and then declines more slowly as the decline is driven only by older FSWs leaving sex work, and being replaced by new FSWs who have not experienced violence).


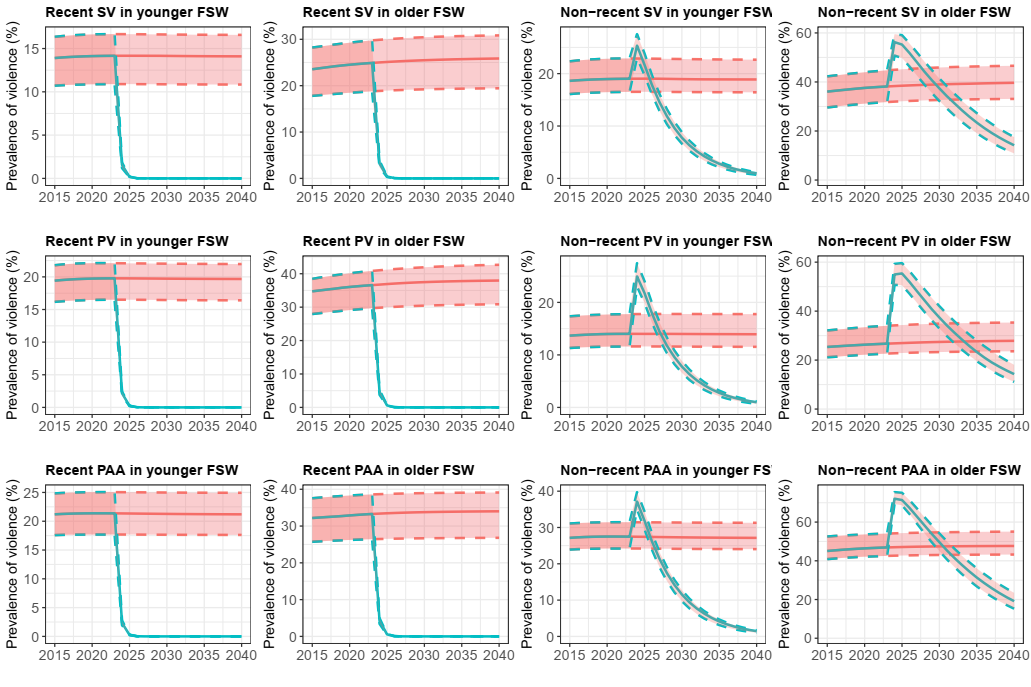


***Intervention impact in FSWs***

Here we show the intervention impact results for FSWs only (the % of infections prevented in FSWs only).

**Figure S15: Impact of violence intervention.** Percentage of infections averted in female sex workers (FSWs) only by different interventions compared to the baseline scenario over 10 and 40 years from 2023. Unless indicated, experience of past violence includes all types of violence. Intervention scenarios are described in Main Text Table 2.


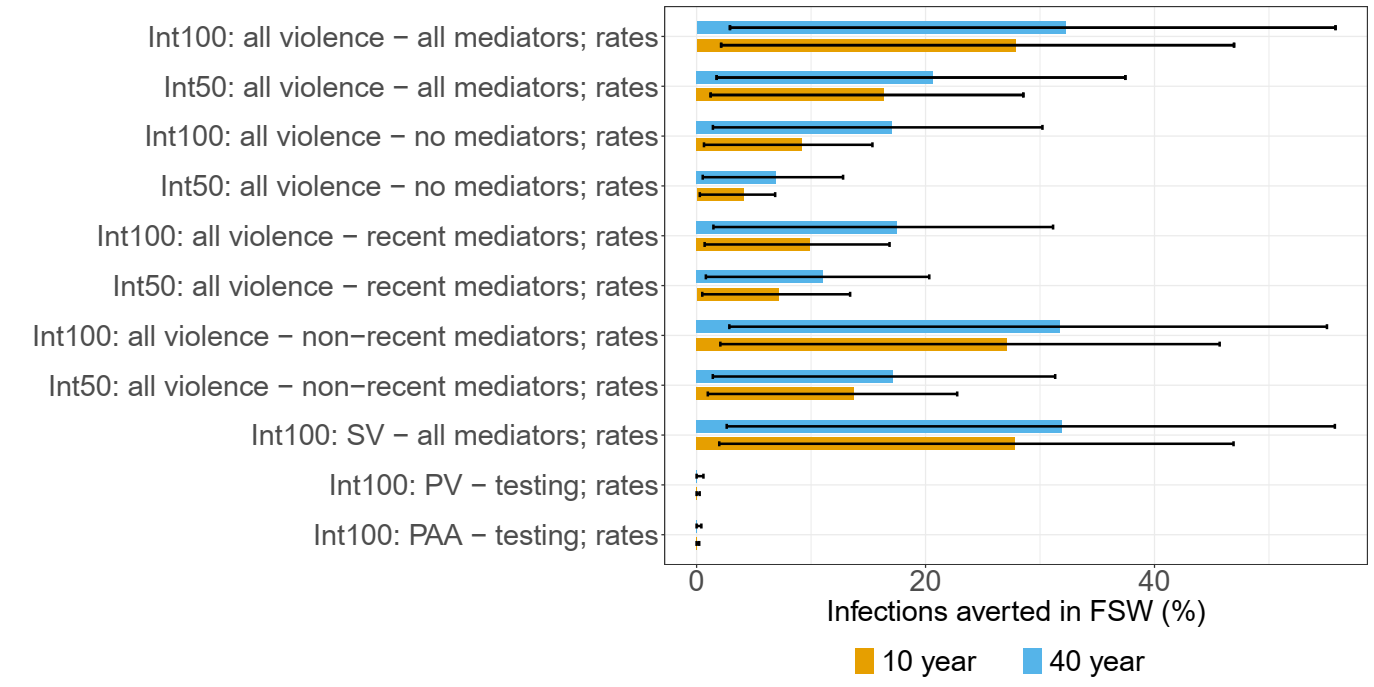


***Intervention impact in clients***

Here we show the intervention impact results for clients only (the % of infections prevented in clients only).

**Figure S16: Impact of violence intervention.** Percentage of infections averted in clients only by different interventions compared to the baseline scenario over 10 and 40 years from 2023. Unless indicated, experience of past violence includes all types of violence. Intervention scenarios are described in Main Text Table 2.


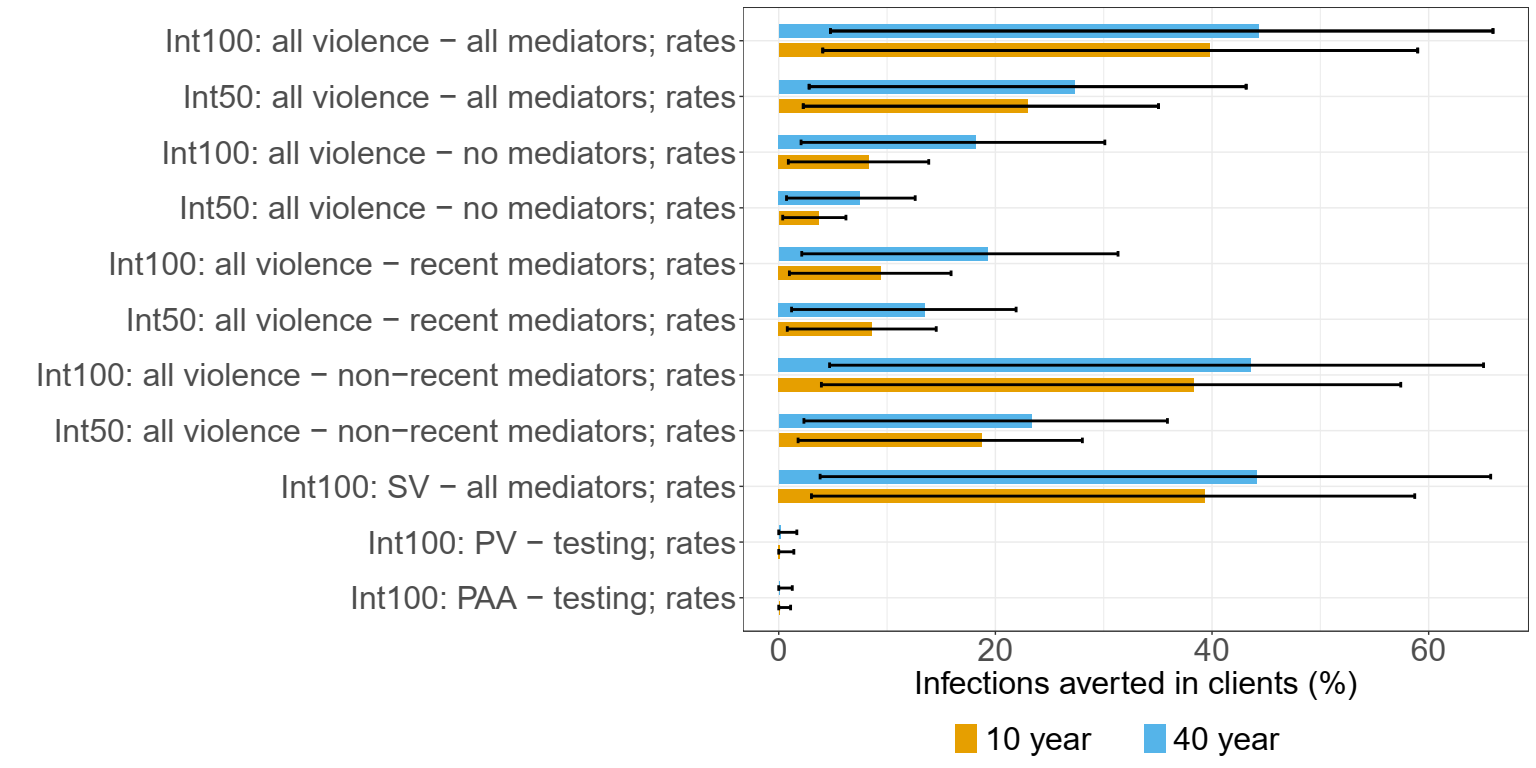


***Parametric sensitivity of tPAF***

Here we use scatter plots to look at correlations between each varied parameter and the 10 year tPAF for the “No effects from violence” scenario. We show scatter plots for all parameters with correlation coefficient >0.2.

Eight parameters have a correlation coefficient >0.2, though only two have a correlation coefficient above 0.6 (Effcond, 0.61; and RRcond_SV: 0.95). Thus, the tPAF depends very strongly on the magnitude of the relative risk of condom non-use in FSWs who have experienced sexual violence (compared to those who have not).

**Figure S17:** Scatter plots of 10 year tPAF (in FSW+clients combined) against the posterior parameter value for all eight parameters with a correlation coefficient>0.2. Correlation coefficient and trend line shown.


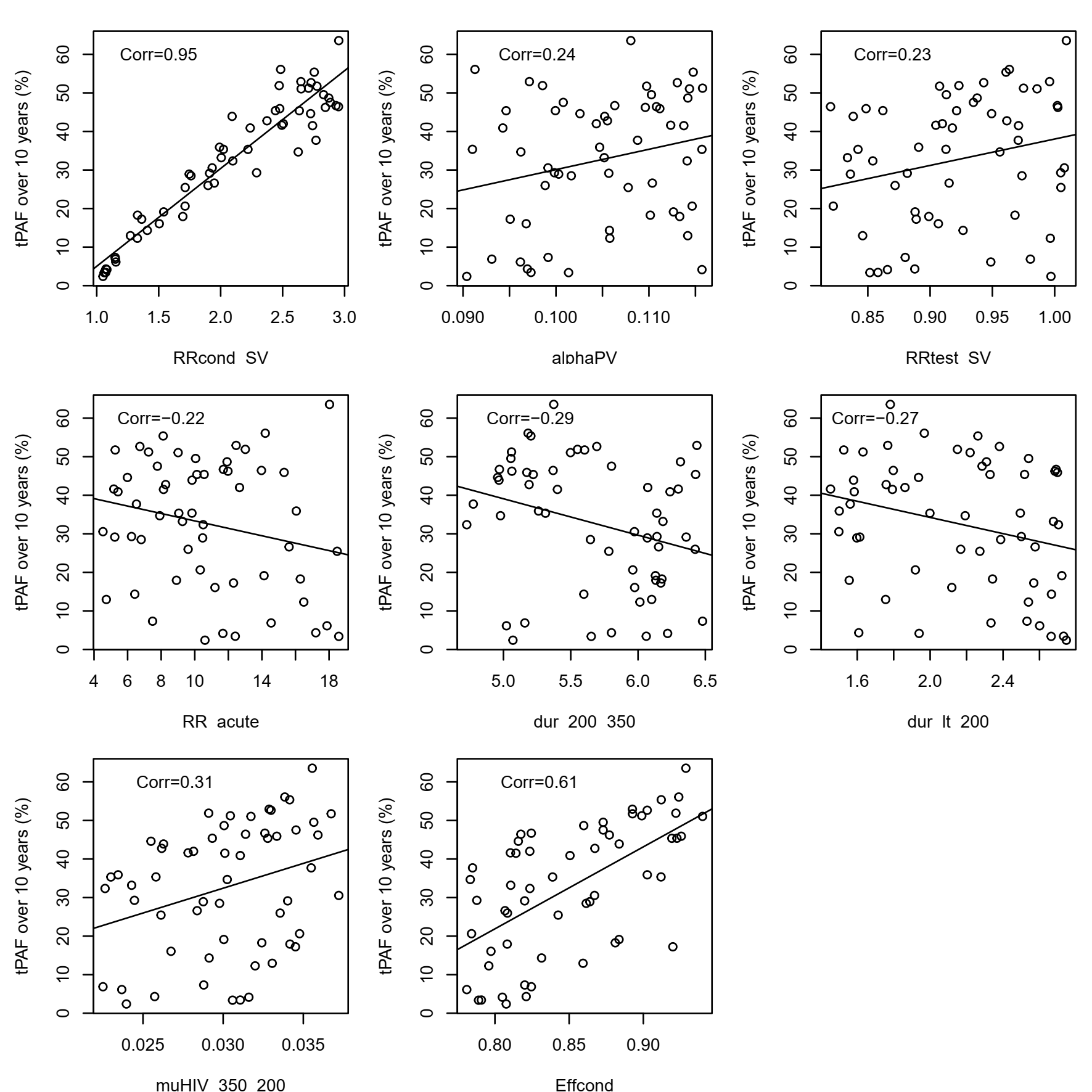


***Parametric sensitivity of intervention impact***

Here we use scatter plots to look at correlations between each varied parameter and the impact (over 10 years) of the violence intervention addressing all types of violence that prevents 100% of both future incident violence and the effects of past violence experience. We show scatter plots for all parameters with correlation coefficient >0.2.

Nine parameters have a correlation coefficient above 0.2, but, as for the earlier tPAF sensitivity analysis, the same two have a coefficient above 0.6 (Effcond, 0.62; and RRcond_SV: 0.94). Thus, both the tPAF and impact of intervention depend very strongly on the magnitude of the risk ratio for condom non-use in FSWs who have experienced sexual violence (compared to those who have not), and the effectiveness of condoms in preventing HIV transmission.

**Figure S18:** Scatter plots of 10 year intervention impact (in FSW+clients combined) for an intervention preventing future violence against the posterior parameter value for all nine parameters with a correlation coefficient>0.2. Correlation coefficient and trend line shown. FSW=female sex worker.


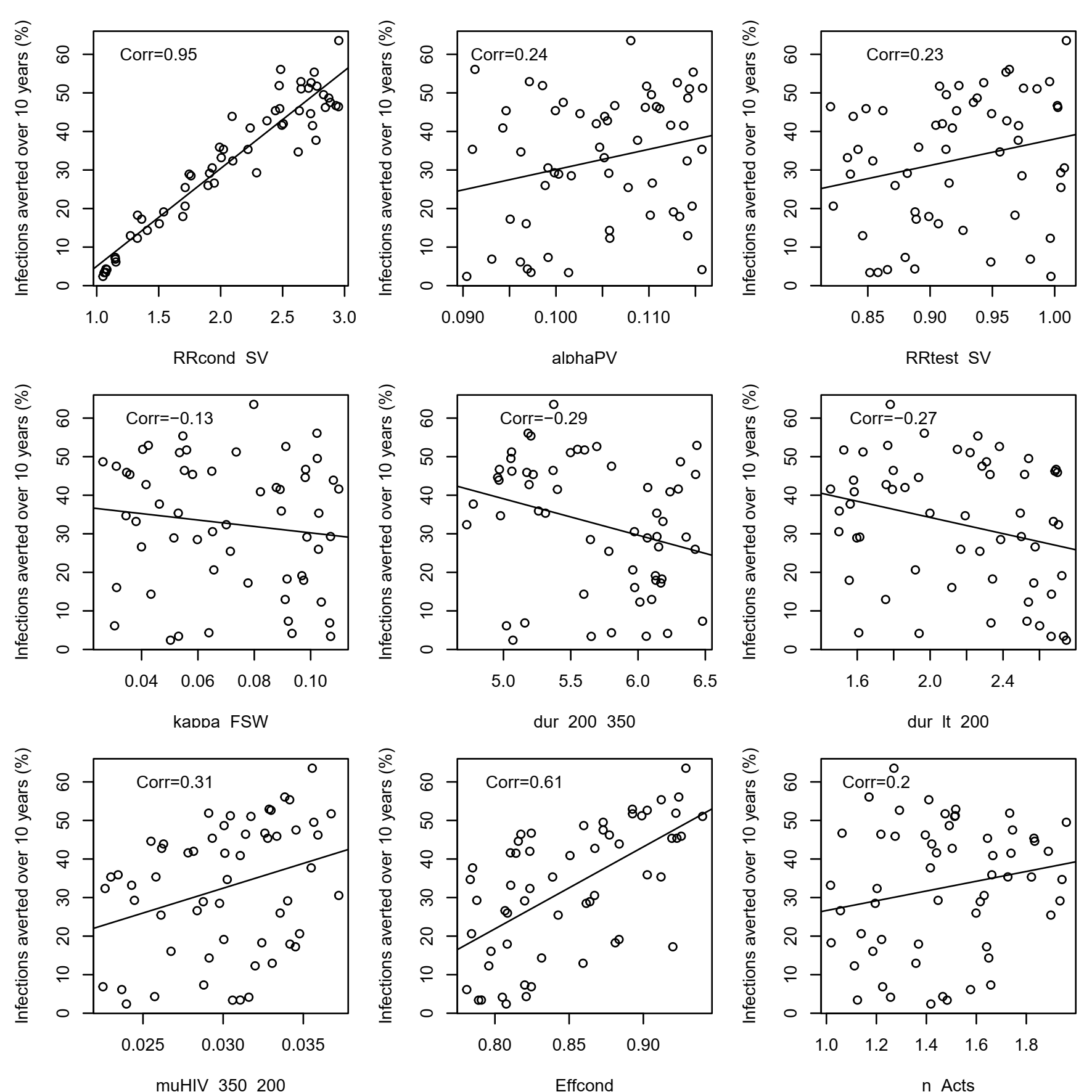


***Results of sensitivity analysis for the assumption about how risk ratios combine when FSWs experience multiple forms of violence***

As can be seen in Figure S19, trends in HIV prevalence and violence are almost indistinguishable from those from the main analysis. There is a very small difference, for example median HIV prevalence in FSWs overall in 2023 is 7.39% (95% CrI 5.59-9.68%) when the risk ratios combine multiplicatively compared to 7.40% (95% CrI 5.58-9.67%) when we take the maximum risk ratio of the experienced violences. Similarly for clients, HIV prevalence in 2023 is 4.78% (3.22-5.92%) compared to 4.76% (3.19-5.93%) in the main analysis. Note that since the only difference between this analysis and the main analysis is ART coverage in FSWs, and we use the same posterior parameter set for both, the two analyses will produce identical output until 2003 when ART starts in the model.

Thus it suffices for the purpose of this sensitivity analysis to use the same posterior parameter set as in the main analysis, rather than recalibrating the model.

**Figure S19: HIV prevalence and violence prevalence for sensitivity analysis.** Figure is a reproduction of Main Text Figure 3, using model outputs from sensitivity analysis where the risk ratios combine multiplicatively when FSWs experience multiple forms of violence (but using the same posterior parameter set as in the main analysis).


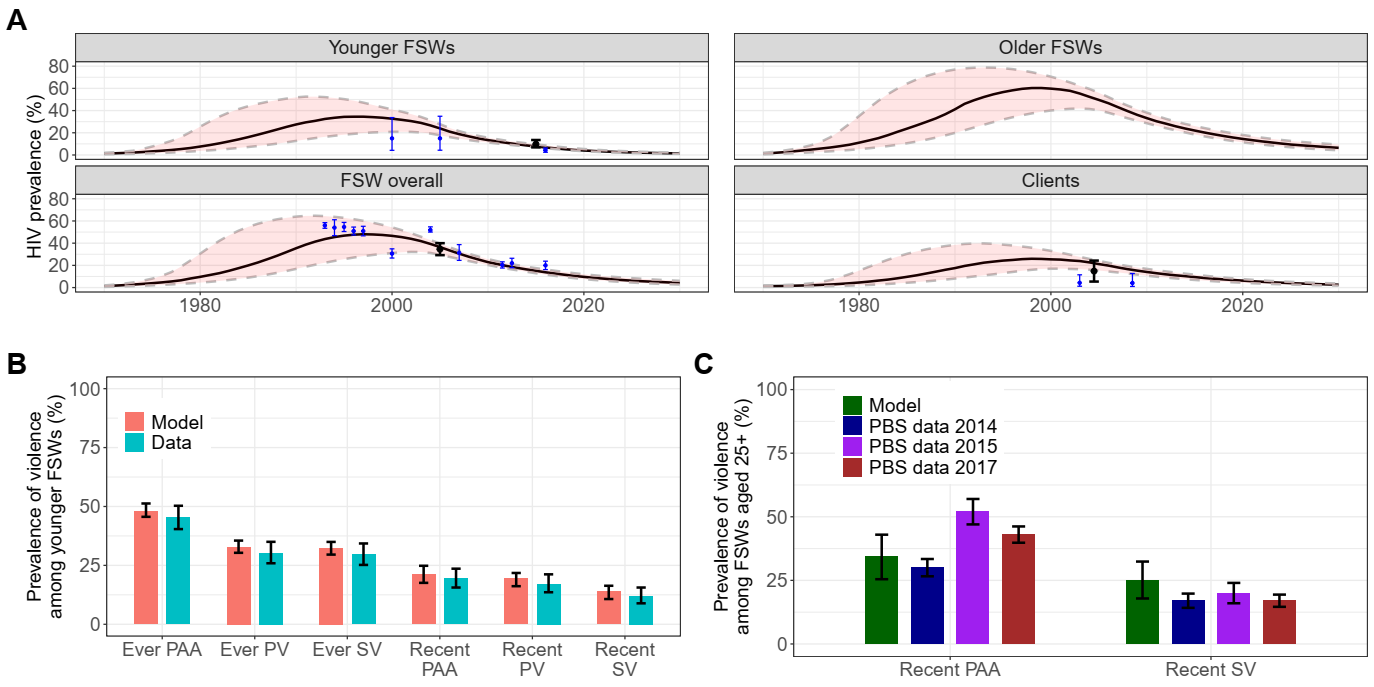


To examine the effects of the assumption of how risk ratios combine, we consider two tPAF scenarios (tPAF from violence on HIV testing, and tPAF from any type of violence) and one intervention impact scenario (Prevent 100% of future violence and mitigate 100% of effects of past violence). Since only HIV testing is affected by violence other than sexual violence, these scenarios are sufficient to show whether results are sensitive to the underlying assumption of how the risk ratios combine.

Figure S20 shows how the impact between the main analysis and sensitivity analysis compare over 10 and 40 years. Overall there is little difference. For the tPAF from any type of violence scenario, and the intervention scenario this is expected, since by far the largest contribution to the tPAF in the main analysis is from the influence of sexual violence on condom use (Main Text Figure 4). As stated in the main text, the influence of violence on the rate of HIV testing (and hence rate of ART initiation among ART-naïve FSWs) is small, even when combined multiplicatively since the individual risk ratios are close to 1 (SV: 0.82-1.01; PV 0.83-1.01; PAA 0.89-1.08 as given in Main Text Table 1), only a small fraction of FSW living with HIV are ART-naïve (median 15.2% in 2023, Figure S9), and increasing ART coverage among FSWs only directly benefits clients.

There is more relative change when looking at the tPAF from violence on HIV testing (the median 10-year tPAF is 1.42 times larger in the sensitivity analysis than in the main analysis) though the absolute change remains small.

Thus in the current setting, where only HIV testing is influenced by multiple forms of violence, and where the effect size of these influences is small, the model is not sensitive to the assumption of how these risk ratios combine.

**Figure S20: Comparison of tPAF and intervention impact between sensitivity analysis and main scenario.** Plots show transmission population-attributable fractions (tPAFs) and in female sex workers (FSWs) and clients combined over 10 (left panel) and 40 (right panel) years from 2023. Bars show median model estimates in original analysis (in green) and sensitivity analysis (yellow), and error bars show 95% credible intervals. Scenario labels are given in Main Text Table 2.


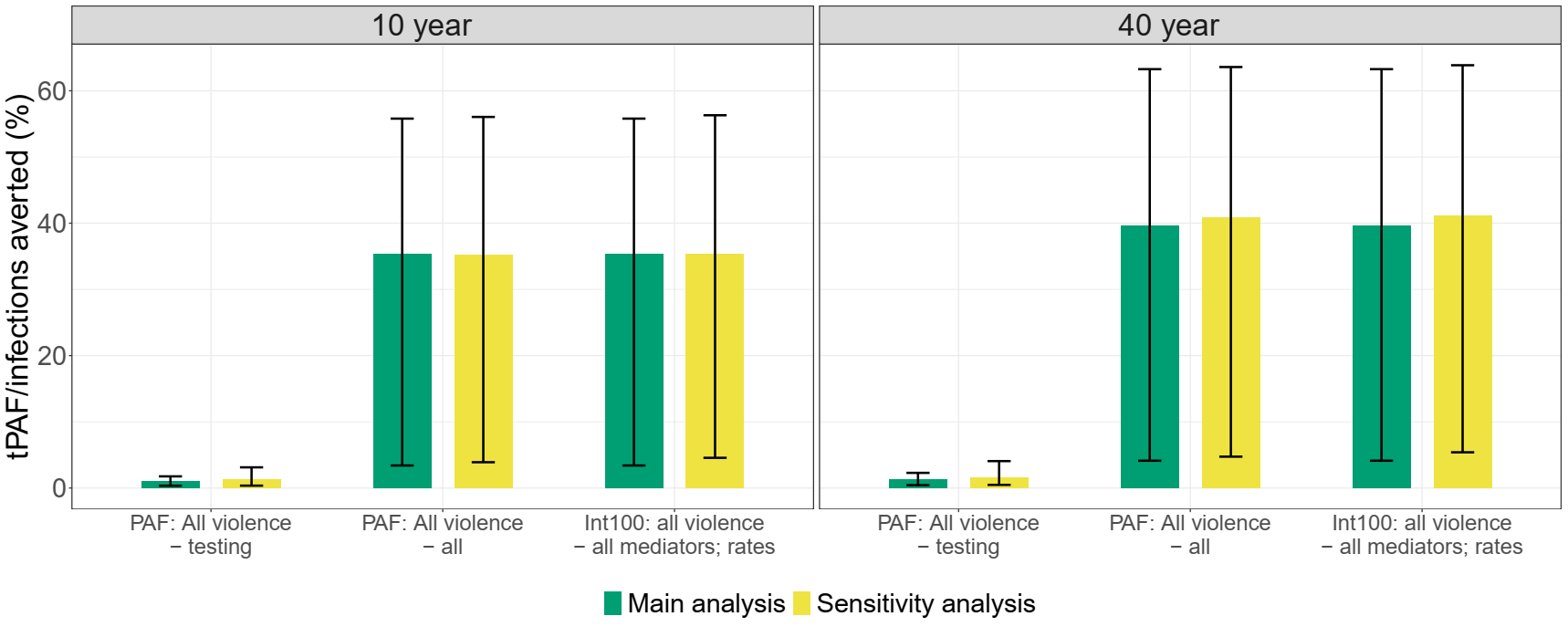


# References

1. Stannah, J., et al., *From conceptualizing to modeling structural factors and interventions: A framework to guide dynamic HIV transmission model development and analysis.* BMC Infect Dis, 2024.

2. Baeten, J.M., et al., *Trends in HIV-1 incidence in a cohort of prostitutes in Kenya: implications for HIV-1 vaccine efficacy trials.* J Acquir Immune Defic Syndr, 2000. **24**(5): p. 458-64.

3. Luchters, S., et al., *Impact of five years of peer-mediated interventions on sexual behavior and sexually transmitted infections among female sex workers in Mombasa, Kenya.* BMC Public Health, 2008. **8**: p. 143.

4. Kavanaugh, B.E., et al., *Prevalence and correlates of genital warts in Kenyan female sex workers.* Sex Transm Dis, 2012. **39**(11): p. 902-5.

5. van der Elst, E.M., et al., *Is audio computer-assisted self-interview (ACASI) useful in risk behaviour assessment of female and male sex workers, Mombasa, Kenya?* PLoS One, 2009. **4**(5): p. e5340.

6. Bengtson, A.M., et al., *Levels of alcohol use and history of HIV testing among female sex workers in Mombasa, Kenya.* AIDS Care, 2014. **26**(12): p. 1619-24.

7. Lafort, Y., et al., *HIV prevention and care-seeking behaviour among female sex workers in four cities in India, Kenya, Mozambique and South Africa.* Trop Med Int Health, 2016. **21**(10): p. 1293-1303.

8. Manguro, G.O., et al., *HIV infections among female sex workers in Mombasa, Kenya: current prevalence and trends over 25 years.* Int J STD AIDS, 2020. **31**(14): p. 1389-1397.

9. Central Bureau of Statistics (CBS) [Kenya], M.o.H.M.K., and ORC Macro,, *Kenya Demographic and Health Survey 2003*, M. CBS, and ORC Macro, Editor. 2004: Calverton, Maryland.

10. Kenya National Bureau of Statistics (KNBS) and ICF Macro, *Kenya Demographic and Health Survey 2008-09*, KNBS and ICF Macro, Editor. 2010: Calverton, Maryland.

11. National AIDS & STI Control Programme, M.o.H., *Third National Behavioural Assessment of Key Populations in Kenya: Polling Booth Survey Report.* 2018, NASCOP: Nairobi, Kenya.

12. National AIDS and STI Control Programme (NASCOP), *Kenya HIV Estimates 2018*. 2018.

13. UNAIDS, *UNAIDS DATA 2022*, Joint United Nations Programme on HIV/AIDS, Editor. 2022: Geneva.

14. National AIDS and STI Control Programme (NASCOP), *Preliminary KENPHIA 2018 Report*, NASCOP, Editor. 2020.

15. Tegang, S.P., et al., *Concurrent sexual and substance-use risk behaviours among female sex workers in Kenya's Coast Province: findings from a behavioural monitoring survey.* SAHARA J, 2010. **7**(4): p. 10-6.

16. Parcesepe, A.M., et al., *Early sex work initiation and condom use among alcohol-using female sex workers in Mombasa, Kenya: a cross-sectional analysis.* Sex Transm Infect, 2016. **92**(8): p. 593-598.

17. National STI/AIDS Control Programme, M.o.H., Kenya, *National Behavioral Assessment of Key Populations in Kenya Polling Booth Survey Report*. 2014, NASCOP: Nairobi, Kenya.

18. National AIDS & STI Control Programme, M.o.H., Kenya,, *Second National Behavioural Assessment of Key Populations in Kenya: Polling Booth Survey Report*. 2016, NASCOP: Nairobi, Kenya.

19. Mountain, E., *HIV risk and prevention among sex workers: a focus on structural determinants and interventions*, in *School of Public Health*. 2017, Imperial College London.

20. UNAIDS, *UNAIDS DATA 2017*, Joint United Nations Programme on HIV/AIDS, Editor. 2017: Geneva.

21. UNAIDS, *UNAIDS DATA 2018*, Joint United Nations Programme on HIV/AIDS, Editor. 2018: Geneva.

22. UNAIDS, *UNAIDS DATA 2019*, Joint United Nations Programme on HIV/AIDS, Editor. 2019: Geneva.

23. UNAIDS, *UNAIDS DATA 2020*, Joint United Nations Programme on HIV/AIDS, Editor. 2020: Geneva.

24. UNAIDS, *UNAIDS DATA 2021*, Joint United Nations Programme on HIV/AIDS, Editor. 2021: Geneva.

25. Stevens, O., et al., *Key population size, HIV prevalence, and ART coverage in sub-Saharan Africa: systematic collation and synthesis of survey data.* medRxiv, 2022: p. 2022.07.27.22278071.
